# An Improved Helferich Method for the α/β-Stereoselective Synthesis of 4-Methylumbelliferyl Glycosides for the Detection of Microorganisms

**DOI:** 10.3390/molecules201219789

**Published:** 2015-12-04

**Authors:** Xianhu Wei, Yanxia Ma, Qingping Wu, Jumei Zhang, Zhihe Cai, Mianfei Lu

**Affiliations:** 1 Guangzhou Institute of Chemistry, Chinese Academy of Sciences, Guangzhou 510650, China; wxhu7508@163.com (X.W.); mayanxia880626@163.com (Y.M.); 2 State Key Laboratory of Applied Microbiology Southern China, Guangdong Institute of Microbiology, Guangzhou 510070, China; zhangjm926@126.com; 3 Guangdong Open Laboratory of Applied Microbiology, Guangdong Institute of Microbiology, Guangzhou 510070, China; 4 Guangdong Provincial Key Laboratory of Microbial Culture Collection and Application, Guangdong Institute of Microbiology, Guangzhou 510070, China; 5 University of Chinese Academy of Sciences, Beijing 100039, China; 6 Guangdong Huankai Microbial Sci. & Tech. Co., Ltd., Guangzhou 510663, China; caizhihe1971@163.com (Z.C.); lumianf@163.com (M.L.)

**Keywords:** fluorogenic substrates, 4-methylumbelliferyl glycosides, stereoselective glycosylation, glycosyl acetates, peracetyl sugars

## Abstract

An improved Helferich method is presented. It involves the glycosylation of 4-methyl-umbelliferone with glycosyl acetates in the presence of boron trifluoride etherate combined with triethylamine, pyridine, or 4-dimethylaminopyridine under mild conditions, followed by deprotection to give fluorogenic 4-methylumbelliferyl glycoside substrates. Due to the use of base, the glycosylation reaction proceeds more easily, is uncommonly α- or β-stereoselective, and affords the corresponding products in moderate to excellent yields (51%–94%) under appropriate conditions.

## 1. Introduction

The detection and identification of microorganisms in food, such as prevalent pathogens, e.g., *Salmonella* and enterohemorrhagic *Escherichia coli*, is essential for realizing and managing microbiological risks to ensure food safety, a worldwide public health concern [1,2,3]. New techniques that are faster and simpler, based on synthetic enzymatic substrates, have been developed and drawn wide attention in recent decades. Synthetic enzymatic substrates are powerful tools in biochemistry that can produce an easily measured output, such as variation of absorbance or fluorescence, to facilitate the detection of enzymatic activities [4,5,6,7,8,9]. For example, 4-methylumbelliferyl glycosides have been widely exploited in diagnostic microbiology [4,5,6,9], in newborn screening of lysosomal storage disorders (LSDs) [10,11,12,13], for the prediction of glycan structures and potential bioactivities of bovine milk [14], characterizing and identifying vegetables [15], and investigating the molecular mechanisms involved in sperm-oocyte binding and gamete-oviductal epithelium interactions [16], by monitoring the specific cellular glycosidases activities to the substrates. These compounds have low toxicity, are stable under physiological conditions, and are easily hydrolyzed by corresponding glycosidase catalysis, which induces strong fluorescence generated by the released 4-methylumbelliferone (4-MU; 7-hydroxy-4-methylcoumarin). As such, they are also considered as ideal molecular probes for glycobiological studies in which a glycosidase activity needs to be assessed *in vitro* or *in situ* research settings [17,18].

Although many such kinds of fluorogenic glycosidase substrates are commercially available, their syntheses are not necessarily straightforward or highly efficient. According to the literature, they are mainly produced by *O*-glycosylation followed by *O*-deacetylation. The methods of *O*-glycosylation primarily include: (1) Michael-type glycosylation with acetylated glycosyl halides [19,20,21,22,23,24,25,26,27]; (2) Helferich glycosylation with glycosyl acetates via a fusion procedure, in boiling xylene in the presence of zinc chloride, or using a stannic chloride catalyst, as in the *O*-trimethylsilylation of 4-MU [27,28]; (3) Koenigs-Knorr glycosylation with glycosyl halides [18,29,30,31]. As well as the methods mentioned above, 4-methylumbelliferyl glucuronides can be produced via direct oxidation of the primary hydroxyl group of 4-methylumbelliferyl glucopyranoside [27,32], indirect oxidation of the primary hydroxyl group of 4-methylumbelliferyl-2,3,4-tri-*O*-acetyl-glucopyranoside followed by deprotection [33], and Schmidt glycosylation with trichloroacetimidate [34,35]. However, these methods suffer from drawbacks that limit their wider or practical application. For example, Michael-type glycosylations in acetone/water often afford low yields [20,21,22,23]; and although relatively higher yields can be obtained under biphasic conditions, as shown in our previous study on the synthesis of 4-methylumbelliferyl β-d-galactopyranoside [24], it is incompatible with glycosyl halides which easily transform into glycals by hydrogen halide elimination under basic conditions, e.g., acetobromomethylglucuronate [26,36]. Silver compounds used as catalysts in Koenigs-Knorr glycosylation are relatively expensive. Compared with other methods especially oxidation and Schmidt glycosylation, glycosyl acetates as glycosyl donors or precursors in the Helferich procedure are more easily available. Therefore, an improved Helferich procedure comprising other advantages, such as stereoselectivity, relatively high yield and easy operation, would be extremely pertinent.

**Scheme 1:**
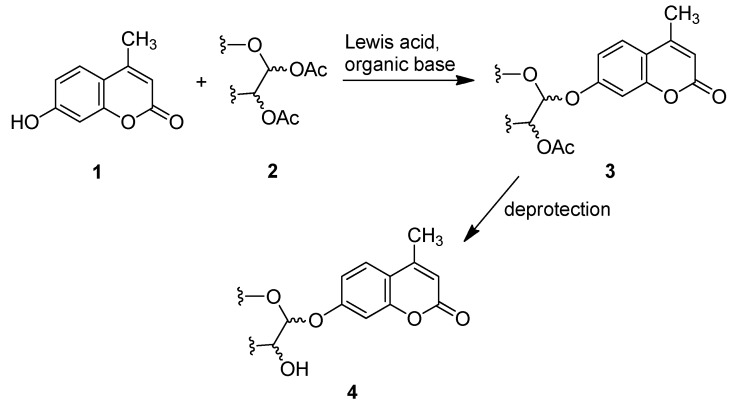
Synthesis of 4-methylumbelliferyl glycosides.

Lee *et al.* reported that the glycosylation of phenol and several substituted phenols with penta-*O*-acetyl-β-d-glucose in the presence of boron trifluoride etherate (BF_3_·OEt_2_) and organic base afforded the products in both high yield and β-stereoselectivity [37]. Herein, we report a modified Helferich procedure for the successful glycosylation of 4-MU with methyl tetra-*O*-acetyl-β-d-glucopyranuronate and several other peracetylated sugars to afford the corresponding protected glycosides with high stereoselectivity under mild conditions, followed by deprotection to produce 4-methylumberlliferyl glycosides products (Scheme 1).

## 2. Results and Discussion

Glucopyranuronides are usually more difficult to prepare than glucopyranosides. There is, in fact, a glycosidation “league table” drawn up by Schmidt *et al.*, ranking in increasing ease of glycosyl donation: glycuronates < aldoses < deoxy sugars < ketoses < 3-deoxy-2-glyculosonates [36]. Of all common sugars, glycuronates therefore require the highest activation for a given aglycone. We envisioned that successful application of the Helferich procedure to the synthesis of 4-methyl-umbelliferyl glucopyranuronide, which has not been previously reported to the best of our knowledge, could be more widely applied to this class of molecules.

### 2.1. Synthesis of the Protected β-d-Glucopyranuronide ***3a***

**Scheme 2:**
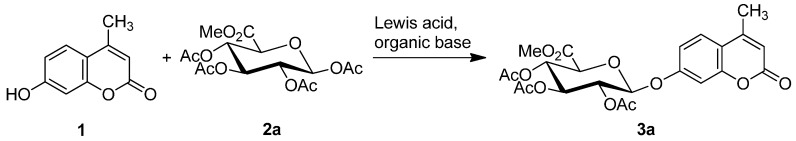
Synthesis of the protected β-d-glucopyranuronide.

In view of this, we started our investigation by using methyl tetra-*O*-acetyl-β-d-glucopyranuronate (**2a**) [38] as a model substrate in the glycosylation of 4-MU (Scheme 2).

As summarized in Table 1, glycosylation of 4-MU (**1**) with methyl tetra-*O*-acetyl-β-d-gluco-pyranuronate (**2a**) under multiple conditions were investigated, and most of reactions gave mainly the protected β-d-glucopyranuronide (**3a**). In the presence of BF_3_·OEt_2_ and TEA, glycosylation at room temperature (25–29 °C) and heating (60 °C) gave mainly the desired product **3a** in the same yield, but the latter case took a much shorter time (entries 1–2). However, **3a** was not detected by thin layer chromatography (TLC) in the absence of TEA (entry 4 *vs.* entry 3). Compared to the report by Lee *et al*. [37] that a TEA to BF_3_·OEt_2_ ratio of more than 0.2 decreased the glycosylation rate and yield, the relatively higher ratio of TEA to BF_3_·OEt_2_ did not decreased the yield, which should be closely related to the poor dissolution of 4-MU in dichloromethane and 1,2-dichloroethane (entry 2 *vs.* entry 5). An excess of 4-MU and the glycosyl donor increased the yield, which was relatively higher in the latter case (entries 5–7). A 4 Å molecular sieve (4 Å MS) is often applied to remove water in glycosylation reactions [39,40,41]. However, its presence here decreased the yield (entry 8). Other organic bases, such as *N*,*N*-diisopropylethyl amine (DIPEA), pyridine, 4-dimethylaminopyridine (DMAP), and 1,1,3,3-tetramethylurea (TMU) were also investigated. Under otherwise identical conditions, glycosylation in the presence of pyridine or TEA afforded the protected β-d-glucopyranuronide in 23% yield, and in the cases of a hindered base (DIPEA) and a very weak base (TMU), the product was obtained in slight lower and significantly lower yields, respectively (entries 7, 9–11). However, a higher yield was obtained upon the use of DMAP, a nucleophilic base (28%, entry 12). In an attempt to achieve complete conversion, we extended the reaction time from 5 to 10 h and increased the amounts of base and Lewis acid. Unfortunately, this resulted in a lower yield of 19% (entry 13 *vs.* entry 10). Changing the solvent from 1,2-dichloroethane to tetrahydrofuran (THF) and CH_3_CN was also unsuccessful, giving no desired product and **3a** in just 2% yield, respectively (entries 14 and 15). Other Lewis acids, such as trimethylsilyl triflate (TMSOTf) and SnCl_4_, were investigated, but afforded **3a** in less than 1% yield and not at all, respectively (entries 16 and 17).

Based on the investigations mentioned above, further optimization was performed to improve the yield of **3a** by using BF_3_·OEt_2_/DMAP/ClCH_2_CH_2_Cl. As summarized in Table 2, further but appropriate increase of the additive amount of **2a**, DMAP and BF_3_·OEt_2_ could gave **3a** in higher yield. When the amount of **2a**, DMAP and BF_3_·OEt_2_ was increased to 2.0 equivalents, 4.0 equivalents and 12.5 equivalents, respectively, the yield of **3a** increased remarkably to 51% (entry 9). Further but much greater excess of DMAP or BF_3_·OEt_2_ caused a remarkable decrease of the yield of **3a**, with obvious increase of the recovery yield of **1**, or yield of by-product **5**, respectively (entries 3, 4, 7, and 10). Besides, a higher temperature (75 °C) also caused a remarkable decrease of the yield of **3a**, with a corresponding obvious increase of the yield of by-product **5** (entry 11).

**Table 1:** Glycosylation of 4-MU (**1**) with methyl tetra-*O*-acetyl-β-d-glucopyranuronate (**2a**).

Entry	1 (Equiv.)	2a (Equiv.)	Organic Base (Equiv.)	Lewis Acid (Equiv.)	Reaction Conditions	Yield ^a^ of 3a	The Main Anomer ^b^
1	1.5	1.0	TEA (3.75)	BF_3_·OEt_2_ (10.0)	CH_2_Cl_2_, 25–29 °C, 72 h	17%	β
2	1.5	1.0	TEA (3.75)	BF_3_·OEt_2_ (10.0)	ClCH_2_CH_2_Cl, 60 °C, 5 h	17%	β
3	1.5	1.0	TEA (3.75)	BF_3_·OEt_2_ (15.0)	ClCH_2_CH_2_Cl, 60 °C, 5 h	19%	β
4	1.5	1.0	— ^c^	BF_3_·OEt_2_ (15.0)	ClCH_2_CH_2_Cl, 60 °C, 10 h	—	—
5	1.5	1.0	TEA (2.0)	BF_3_·OEt_2_ (10.0)	ClCH_2_CH_2_Cl, 60 °C, 5 h	16%	β
6	2.0	1.0	TEA (2.0)	BF_3_·OEt_2_ (10.0)	ClCH_2_CH_2_Cl, 60 °C, 5 h	18%	β
7	1.0	1.5	TEA (2.0)	BF_3_·OEt_2_ (10.0)	ClCH_2_CH_2_Cl, 60 °C, 5 h	23%	β
8	1.0	1.5	TEA (2.0)	BF_3_·OEt_2_ (10.0)	ClCH_2_CH_2_Cl, 4A MS, 60 °C, 5 h	13%	β
9	1.0	1.5	DIPEA (2.0)	BF_3_·OEt_2_ (10.0)	ClCH_2_CH_2_Cl, 60 °C, 5 h	20%	β
10	1.0	1.5	Pyridine (2.0)	BF_3_·OEt_2_ (10.0)	ClCH_2_CH_2_Cl, 60 °C, 5 h	23%	β
11	1.0	1.5	TMU (2.0)	BF_3_·OEt_2_ (10.0)	ClCH_2_CH_2_Cl, 60 °C, 5 h	6%	β
12	1.0	1.5	DMAP (1.5)	BF_3_·OEt_2_ (10.0)	ClCH_2_CH_2_Cl, 60 °C, 5 h	28%	β
13	1.0	1.5	Pyridine (3.0)	BF_3_·OEt_2_ (15.0)	ClCH_2_CH_2_Cl, 60 °C, 10 h	19%	β
14	1.0	1.5	Pyridine (3.0)	BF_3_·OEt_2_ (15.0)	THF, 60 °C, 10 h	—	—
15	1.0	1.5	Pyridine (3.0)	BF_3_·OEt_2_ (15.0)	CH_3_CN, 60 °C, 10 h	2%	β
16	1.0	1.5	Pyridine (2.0)	TMSOTf (4.0)	ClCH_2_CH_2_Cl, 60 °C, 16 h	<1%	β
17	1.5	1.0	DIPEA (3.0)	SnCl_4_ (5.0)	CH_2_Cl_2_, 25–29 °C, 48 h	—	—

^a^ Isolated yield; ^b^ The main anomer was determined by TLC analysis and isolation; ^c^ None.

**Figure 1:**
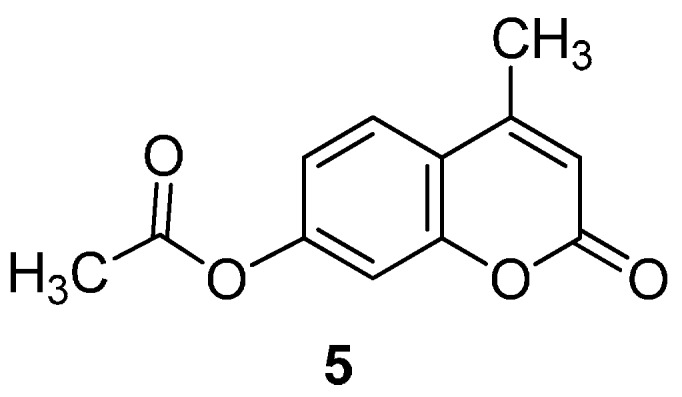
4-methylumbelliferyl acetate (by-product **5**).

Table 2 also suggests that other products were generated in the reactions, as indicated by the fact that the total combined yield of **3a**, yield of recovered **1** and yield of by-product **5** (Figure 1) ranged from 65% to 75%. Herein, lots of small equivalent tests were performed in order to more conveniently and more quickly know the main glycosylation product and its yield and investigate multiple but different factors to improve the yield. Considering that the original Helferich procedure with methyl tetra-*O*-acetyl-β-d-glucopyranuronate was stereochemically reliable, giving only β-d-glucuronide (**3a**) [36], we did not search for the other anomer glycosylation product, perhaps generated but in a low yield below the limits of our detection, and other by-products.

**Table 2:** Further optimization of conditions for synthesis of the protected β-d-glucopyranuronide (**3a**).

Entry ^a^	1 (mmol)	2a (mmol)	DMAP (mmol)	BF_3_·OEt_2_ (mmol)	Yield of Recovered 1 ^b^	Yield ^c^		Main Anomer ^d^
						3a	5	
1	1.0	1.5	3.0	10.0	21%	30%	14%	β
2	1.0	1.5	4.5	10.0	28%	32%	11%	β
3	1.0	1.5	6.0	10.0	44%	17%	9%	β
4	1.0	1.5	5.0	10.0	37%	23%	10%	β
5	1.0	1.5	4.0	10.0	24%	34%	12%	β
6	1.0	1.5	3.5	10.0	26%	33%	13%	β
7	1.0	3.0	8.0	20.0	22%	36%	14%	β
8	1.0	2.0	5.0	12.5	11%	49%	12%	β
9	1.0	2.0	4.0	12.5	9%	51%	15%	β
10	1.0	2.0	4.0	15.0	10%	41%	17%	β
11	1.0	2.0	4.0	12.5	15%	26%	26%	β

^a^ All the reactions were performed in 1,2-dichloroethane at 60 °C (except for entry 11 at 75 °C) for 5 h, then the cooled reaction mixtures were directly separated by gradient-flash column chromatography (silica gel, 200–300 mesh, PE/EtOAc, 7/2, 3/1, 5/2); ^b,c^ The yield of recovered **1** and the yields of **3a** and **5** refer to isolated products or determined by isolation and HPLC analysis; ^d^ The main anomer was determined by TLC analysis and isolation.

### 2.2. Synthesis of Other Protected Glycosides ***3b***–***3f***

**Scheme 3:**
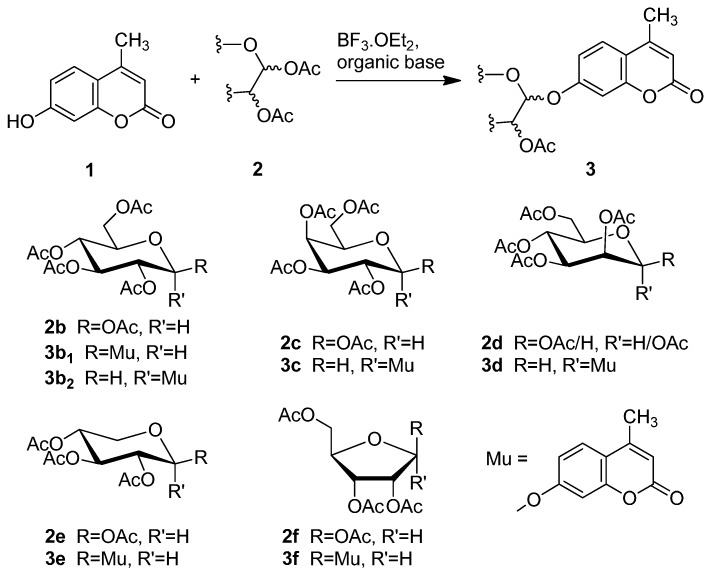
Synthesis of other protected glycosides.

Based on the abovementioned success, the glycosylation of 4-MU with some other peracetyl sugar donors: peracetyl glucopyranose (**2b**), peracetyl galactopyranose (**2c**), peracetyl mannopyranose (**2d**), peracetyl xylopyranose (**2e**) [42] and peracetyl ribofuranose (**2f**), was also investigated (Scheme 3). In view of the differences between the different peracetyl sugars in aspects such as reactivity and stability, we speculated that the abovementioned optimal conditions may not be optimal for other peracetyl sugars. Hence multiple conditions including a TEA or pyridine to BF_3_·OEt_2_ ratio of 0.2 referred to Lee *et al*.’s method [37] were used to study the glycosylation as follows.

As summarized in Table 3, all reactions proceeded smoothly and gave the glycoside products in moderate to excellent yields. In the presence of a relatively lower amount of BF_3_·OEt_2_ and DMAP, the glycosidation of **1** with **2b** afforded mainly the protected β-d anomer product (**3b_1_**) in 36% yield, and the use of a higher amount of pyridine gave **3b_1_** in 42% yield (entries 1 and 2).

When a relatively high amount of BF_3_·OEt_2_ and DMAP was used, the reaction gave **3b_1_** in 45% yield; In addition, the protected α-d anomer product (**3b_2_**) was unexpectedly obtained in a low yield of 7% (entry 3). The glycosidation of **1** with **2b** in the absence of base afforded no products by TLC analysis (entry 4). We expected the glycosylation procedure to be β-stereoselective for the peracetyl sugars, but this was changed by the results obtained with **2c**. Compound **2c** afforded mainly the protected α-d-galactopyranoside product (**3c**) in 54% yield at 60 °C, and 17% yield at 0 °C in the presence of TEA, and 56% yield at 60 °C in the presence of DMAP, respectively (entries 5–7). In view of the facts that an excess of peracetyl sugar donors causes purification difficulties and an excess of 4-MU could be removed easily by aqueous sodium hydroxide solution, and that **2d** should have relatively high reactivity, we investigated the glycosylation of excess of 4-MU with **2d** as a mixture of α- and β-d anomers in the presence of pyridine. This case gave mainly the protected α-d-mannopyranoside product **3d** in 59% yield that was very pure after three recrystallizations (entry 8). Using an excess of **2d** in the presence of DMAP gave **3d** in a slight higher yield of 62% (entry 9). these two 4-methylumbelliferyl α-d-pyranosides were also obtained by the early Helferich method: in 1970 Vervoot *et al.* reported that the condensation of 4-MU with α-d-mannose pentaacetate by a method using fusion treatment and a zinc chloride catalyst under diminished pressure gave the protected α-d-mannopyranoside (**3d**) in 37% yield [28]. In 1978 Courtin-Duchateau *et al.* reported that the condensation of 4-MU with α-d-galactose pentaacetate (**2c**) in boiling xylene in the presence of zinc chloride gave a mixture of protected α-d-galactopyranoside, protected β-d-galactofuranoside and protected β-d-galactopyranoside in a ratio of 3:5:7 in 30% total yield; and condensation of the *O*-trimethylsilyl derivative of 4-MU with β-d-galactose pentaacetate (**2c**) in the presence of stannic chloride gave a mixture of α and β-d-galactopyranoside in a ratio of 15:4 in low total yield of 19%. The protected α-d-mannopyranoside **3d** and α-d-galactopyranoside **3c** could also be obtained stereoselectively by condensation of the sodium salt of 4-MU with the corresponding *O*-acetylated glycosyl chlorides in hexamethylphosphoric triamide, but the reaction time was long (a few days) and the products were obtained in only 30% and 47% yields, respectively [27]. Compared with the reported methods mentioned above, the improved method for **3d** and **3c** here shows higher efficiency, higher yields and high stereoselectivity. 4-Methylumbelliferyl β-d-xylopyranoside (**4e**) was prepared in 1965 by De Bruyne *et al.*, who reported a 35% yield by the Michael condensation of acetobromoxylose with 4-MU in acetone/water [43]. Here the improved Helferich condensation of β-d-xylose tetraacetate (**2e**) with 4-MU at room temperature (20–27 °C) gave mainly the protected β-d-xylopyranoside (**3e**) in 73% and 69% yields in the presence of TEA and DMAP, respectively, which is more than about twice as high as De Bruyne *et al.*’s method (entries 10 and 11); it should also be noted that the reaction gave a very low yield at 50–60 °C by comparative TLC analysis. 4-Methyl-umbelliferyl β-d-ribofuranoside (**4f**) was prepared in 1997 by Schramm *et al.*, who reported just a 25% yield using a variant of the Koenigs-Knorr condensation of *O*-benzoylated ribofuranosyl chloride with the silver salt of 4-MU in toluene under reflux [44]. In this study, the condensation of β-d-ribofuranose tetraacetate (**2f**) with 4-MU at room temperature (20–27 °C) afforded mainly the protected β-d-ribofuranoside **3f** in much higher yields of 94% and 77% in the presence of TEA and DMAP, respectively (entries 12 and 13). Moreover, the method used here was far simpler. The use of DMAP gave lower yields than of TEA for glycosylation with **3e** and **3f**, which suggested that optimal conditions of glycosylation of 4-MU with different glycosyl acetate donors should be different. And here comparison between multiple conditions used was necessary and helpful.

An additional noteworthy detail was the following: the cooled reaction mixtures were directly quenched and separated by column chromatography (silica gel, 200–300 mesh) with a view to preventing 4-MU from dissolving in water or aqueous alkaline solution to determine its recovery yield. This reaction post-processing was relative simpler, however, it may be affected by different silica gels of different brands, due to the fact that the single use of Macklin silica gel unexpectedly gave a remarkable lower yield of **3a** compared with any of numerous examples using Haiyang silica gel, all other conditions being basically equal.

An explanation for the above stereoselective glycosylation is presented as follows: it is reported that base can abstract the proton quickly from the adduct intermediate cation generated via phenol attack on the acetyloxonium ion intermediate in the glucosidation and prevent anomerization of the β-anomer to the α-anomer [45]. Here base should also have a role in preventing anomerization in the glycosylation of 4-MU with glycosyl acetates. Besides, one of the most powerful principles of the enforced 1,2-*trans* glycosylation is neighboring group participation by the acyl group at C-2 (generation of the intermediate acyloxonium ion) [46,47] that can be applied to further explain the selective formation of the protected glycosides **3a**, **3b_1_**, **3d**, **3e**, and **3f**. However, how can the selective formation of the protected α-d-galactopyranoside **3c** that is a 1,2-*cis* glycoside be explained? It is reported that the acyl group at C-4 of a galactosyl donor with a non-participating substitute at C-2 can effect a remote participation effect that is beneficial for the formation of 1,2-*cis* galactosidic bonds [46,48,49]. Therefore, the selective formation of **3c** may be attributed to the participation of the acetyl group at C-2 followed by participation of the one at C-4 due to the fact the reactivity of 4-MU may be weaker compared to the acetyl groups at C-2 and C-4 of β-d-galactose pentaacetate, beside its role as base.

### 2.3. Deprotection of the Protected Glycosides ***3a***–***3f***

**Scheme 4:**
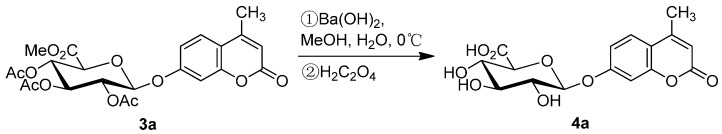
Deprotection of the protected β-d-glucopyranuronide **3a**.

The deprotection step for glucuronides is different from that of other glycosides. In particular, there are more problems that need to be considered in order to obtain the free glucuronic acid. Due to the possibility of elimination as a side reaction, generating Δ^4,5^-(dehydro) glucuronide, and opening of the umbelliferone lactone, many deprotection methods including chemical and enzymatic treatments have been used, as well as the hydrolysis of Na_2_CO_3_ in aqueous MeOH, followed by desalting using a cation exchange resin or acidification followed by reverse-phase column chromatography treatment [34,36,50,51,52,53,54]. Here, a modified method for the deprotection of **3a** was used (Scheme 4). Namely, using excess of Ba(OH)_2_ hydrate in aqueous MeOH in an ice water bath, the barium salt of **4a** was obtained, that was insoluble in MeOH. This was then acidified using H_2_C_2_O_4_ hydrate in fresh MeOH, which can improve the practicality of the procedure by avoiding the difficulty of evaporating the product solution containing a lot of water.

**Table 3:** Glycosylation of 4-MU (**1**) with other peracetyl sugars (**2b**–**2f**).

Entry ^a^	1 (Equiv.)	2 (Equiv.)	Organic Base (Equiv.)	BF_3_·OEt_2_ (Equiv.)	Reaction Conditions	Yield ^b^	The Main Anomer ^c^
1	1.0	**2b** (2.0)	DMAP (1.5)	10.0	ClCH_2_CH_2_Cl, 60 °C, 5 h	**3b_1_** (36%)	β
2	1.0	**2b** (2.0)	Pyridine (4.5)	12.5	ClCH_2_CH_2_Cl, 60 °C, 5 h	**3b_1_** (42%)	β
3 ^d^	1.0	**2b** (2.0)	DMAP (4.0)	12.5	ClCH_2_CH_2_Cl, 60 °C, 5 h	**3b_1_** (45%)	β
4	1.0	**2b** (2.0)	— ^e^	12.5	ClCH_2_CH_2_Cl, 60–70 °C, 10 h	—	—
5	1.0	**2c** (2.0)	TEA (2.5)	12.5	ClCH_2_CH_2_Cl, 60 °C, 5 h	**3c** (54%)	α
6	1.0	**2c** (2.0)	TEA (2.5)	12.5	ClCH_2_CH_2_Cl, 0 °C, 48 h	**3c** (17%)	α
7 ^f^	1.0	**2c** (2.0)	DMAP (4.0)	12.5	ClCH_2_CH_2_Cl, 60 °C, 5 h	**3c** (56%)	α
8	2.0	**2d** (1.0)	Pyridine (3.0)	15.0	ClCH_2_CH_2_Cl, 60 °C, 5 h	**3d** (59%)	α
9 ^g^	1.0	**2d** (2.0)	DMAP (4.0)	12.5	ClCH_2_CH_2_Cl, 60 °C, 5 h	**3d** (62%)	α
10	1.0	**2e** (2.0)	TEA (2.5)	12.5	CH_2_Cl_2_, 20–21 °C, 5 h	**3e** (73%)	β
11 ^h^	1.0	**2e** (2.0)	DMAP (4.0)	12.5	ClCH_2_CH_2_Cl, 26–27 °C, 5 h	**3e** (69%)	β
12	1.0	**2f** (2.0)	TEA (2.5)	12.5	CH_2_Cl_2_, 20–21 °C, 3 h	**3f** (94%)	β
13 ^i^	1.0	**2f** (2.0)	DMAP (4.0)	12.5	ClCH_2_CH_2_Cl, 26–27 °C, 5 h	**3f** (77%)	β

^a^ The entries 3, 7, 9, 11 and 13 were performed according to the procedure in Table 2, and the recovery yield of **1** and the yield of by-product **5** were determined by isolation and HPLC analysis; ^b^ Isolated yield; ^c^ The main anomer was determined by TLC analysis and isolation; ^d^ The reaction gave α-d anomer product (**3b_2_**) and by-product **5** in 7% and 16% yields, respectively, and the recovery yield of **1** was 8%; ^e^ None; ^f^ The reaction gave by-product **5** in 15% yield, and the recovery yield of **1** was 9%; ^g^ The reaction gave by-product **5** in 12% yield, and the recovery yield of **1** was 8%; ^h^ The reaction gave by-product **5** in 3% yield, and the recovery yield of **1** was 13%; ^i^ The reaction gave no by-product **5**, and the recovery yield of **1** was 20%.

**Scheme 5:**
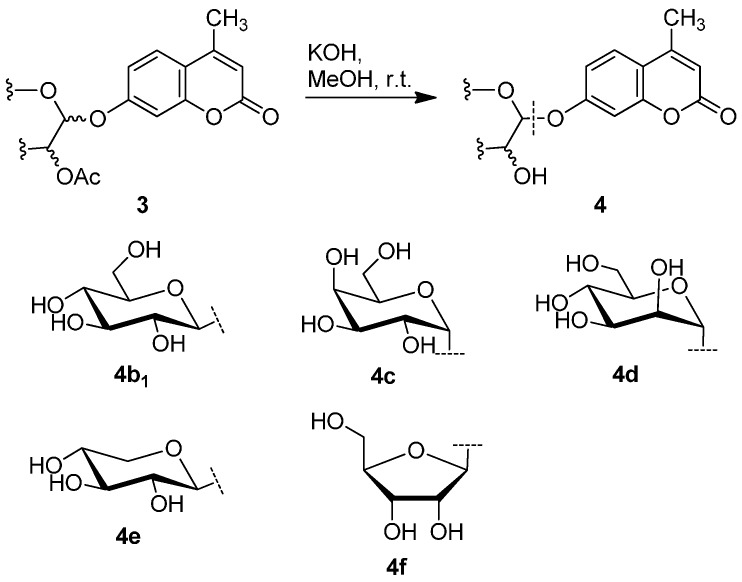
Deprotection of the other protected glycosides **3b_1_**–**3f**.

Using this unoptimized method, **4a** was obtained in 47% yield. The deprotection of other glycosides **3b_1_**, **3c**, **3d**, **3e**, and **3f** via methanolysis catalyzed by KOH at room temperature (r.t., 20–25 °C; Scheme 5), afforded the desired products **4b_1_**, **4c**, **4d**, **4e**, and **4f** in 83%, 80%, 80%, 79%, and 79% yields, respectively. Due to little amount obtained, **3b_2_** was not deprotected.

## 3. Experimental Section

### 3.1. General Information

Methyl tetra-*O*-acetyl-β-d-glucopyranuronate (**2a**), and β-d-xylopyranose tetraacetate (**2e**) were prepared according to the references [38,42], respectively. α/β-d-Mannopyranose pentaacetate (**2d**) was prepared as specified in this section. Other reagents and all organic solvents were purchased from commercial sources and were of analytical reagent grade or contained the desired chemical in a purity of more than 97%. Those that were used as reaction solvents were dried prior to use. Petroleum ether (PE) refers to the fraction boiling in the 60–90 °C range. TLC was performed using silica gel GF-254 plates (purchased from Qingdao Haiyang Chemical Co., Ltd., Qingdao, China) with detection by iodine fumigation or 15% H_2_SO_4_–EtOH/heating or UV (254 nm and 365 nm) (Shanghai JiaPeng Technology Co., Ltd., Shanghai, China), or charring with 20% H_2_SO_4_ in EtOH. Column chromatography was performed on silica gel (200–300 mesh, purchased from Qingdao Haiyang Chemical Co., Ltd., Qingdao, China) using a PE-EtOAc system as eluent. Organic solutions were distilled on a rotary evaporator at 35–40 °C or 40–45 °C. ^1^H- and ^13^C-NMR spectra were recorded on 300 and 500 MHz NMR spectrometers (Bruker (Beijing) Scientific Technology Co. Ltd., Beijing, China). ^1^H-NMR spectra were recorded at 300 or 500 MHz in CDCl_3_ or DMSO-*d*_6_ solvent. ^13^C-NMR spectra were recorded at 75 MHz in CDCl_3_ or DMSO-*d*_6_ solvent. HRMS spectra were recorded on an ultrahigh-resolution quadrupole time-of-flight (UHR-Q-TOF) mass spectrometer (Bruker (Beijing) Scientific Technology Co. Ltd., Beijing, China) equipped with an ESI source. Specific rotations were measured with an automatic digital polarimeter (Sinoinstrument Co. Ltd., Guangzhou, China) at 19–21 °C. Melting points were measured with a microscope melting point apparatus (Shanghai Precision & Scientific Instrument Co., Ltd., Shanghai, China).

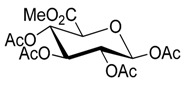


*Methyl tetra-O-acetyl-β-d-glucopyranuronate* (**2a**). This compound was prepared according to a literature procedure [38]; white powder; mp: 176–178 °C; {lit. [38] mp: 174.2–175.1 °C}. ^1^H-NMR (300 MHz, CDCl_3_): δ = 5.78 (d, *J*_1,2_ = 7.7 Hz, 1H, H-1), 5.37–5.13 (m, 3H, H-2, H-3, H-4), 4.20 (d, *J* = 9.4 Hz, 1H, H-5), 3.77 (s, 3H, Me), 2.14 (s, 3H, OAc), 2.06, 2.06, 2.05 (3× s, 9H, 3× OAc). ^13^C-NMR (75 MHz, CDCl_3_): δ = 169.88, 169.40, 169.16, 168.81, 166.79, 91.31 (C-1), 72.93, 71.77, 70.11, 68.88, 53.00, 20.75, 20.54, 20.51, 20.44. ^1^H- and ^13^C-NMR spectrograms are seen in the Supplement Materials.

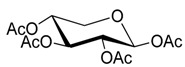


*β-d-Xylopyranose tetraacetate* (**2e**) This compound was prepared according to a literature procedure [42]; white acicular crystals; mp: 123–125 °C; {lit. [42] mp: 122–124 °C}. ^1^H-NMR (300 MHz, CDCl_3_): δ = 5.73 (d, *J*_1,2_ = 6.9 Hz, 1H, H-1), 5.22 (t, *J* = 8.3 Hz, 1H, H-4), 5.10–4.96 (m, 2H, H-2, H-3), 4.17 (dd, *J* = 12.0, 5.0 Hz, 1H, H-5a), 3.54 (dd, *J* = 12.0, 8.4 Hz, 1H, H-5b), 2.13 (s, 3H, OAc), 2.08, 2.07, 2.07 (3× s, 9H, 3× OAc). ^13^C-NMR (75 MHz, CDCl_3_): δ = 169.76, 169.73, 169.23, 168.96, 91.96 (C-1), 70.90, 69.41, 68.24, 62.70, 20.73, 20.65, 20.59, 20.53. ^1^H- and ^13^C-NMR spectrograms are seen in the Supplement Materials.

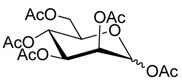


*α/β-d-Mannopyranose pentaacetate* (**2d**) Acetic anhydride (25 mL) and pyridine (20 mL) were added successively under magnetic stirring to d-(+)-mannose (5.00 g, 27.75 mmol) in an ice-water bath. After approximately 4 h, a clear and colorless liquid was obtained and stirring in an ice-water bath was continued for 20 h. Distilled water (55 mL) was then added. After stirring for approximately 5 h, the mixture was left to stand at 4 °C in a refrigerator for 24 h, then was extracted with CH_2_Cl_2_ (45 mL). The organic phase was washed successively with dilute HCl (1 M), saturated aqueous NaHCO_3_, distilled water and saturated aqueous NaCl, dried with anhydrous Na_2_SO_4_. After removing the solvent under reduced pressure at 35–40 °C, a crude product was obtained and further dried in a vacuum desiccator. The constant weight crude product was a clear, colorless and sticky syrup (10.51 g, 97%), and it was used directly for glycosylation.

### 3.2. General Procedure for the Glycosylation Step

To a mixture of 4-MU (1.0–6.0 mmol) and glycosyl acetate (1.0–4.0 mmol) under an argon atmosphere, dry solvent was added successively, followed by the corresponding molar equivalents of base and Lewis acid. The mixture was stirred for a set amount of time at a certain temperature (as shown in Table 1, Table 2 and Table 3). Then, an equal volume of CH_2_Cl_2_ was added to dilute the reaction mixture, and the reaction was quenched with saturated aqueous NaHCO_3_. The organic phase was washed with diluted aqueous NaOH (1 M) until the aqueous phase was a light brownish-yellow or almost colorless, then washed successively with distilled water, saturated aqueous NaCl, dried with anhydrous Na_2_SO_4_, and the solvent was removed under reduced pressure. The crude product was purified by flash column chromatography (silica gel, 200–300 mesh, PE/EtOAc, 5/2), then the desired product was crystallized from anhydrous ethyl ether and dried, or the crude product was purified by several recrystallizations from ethanol.

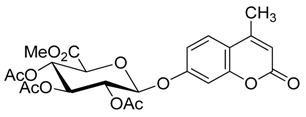


*Methyl (4′-methylumbelliferyl 2,3,4-tri-O-acetyl-β-d-glucopyranosid)urinate* (**3a**) Compound **3a** was prepared according to the general procedure for the glycosylation step using 4-MU (176–352 mg, 1.0–2.0 mmol), glycosyl acetate (376–564 mg, 1.0–1.5 mmol), dry solvent (3 mL), corresponding molar equivalents of base and Lewis acid. The mixture was stirred for a set amount of time at a certain temperature (as shown in Table 1 and Table 2). The crude product was purified by flash column chromatography. White powder; mp: 187–190 °C; ${\left[\alpha \right]}_{D}^{19}$ −98° (*c* = 0.40, CHCl_3_); *R_f_* = 0.26 (PE–AcOEt, 3:2); {lit. [29] mp: 189–190 °C, ${\left[\alpha \right]}_{D}^{20}$ −45° (*c* = 1, CHCl_3_); lit. [35] *R_f_* = 0.2 (hexanes–EtOAc, 2:3)}. ^1^H-NMR (300 MHz, CDCl_3_): δ = 7.55 (d, *J*_5′,6′_ = 9.4 Hz, 1H, H-5′), 6.97–6.93 (m, 2H, H-6′, H-8′), 6.22 (s, 1H, H-3′), 5.43–5.24 (m, 4H, H-1, H-2, H-3, H-4), 4.29–4.22 (m, 1H, H-5), 3.76 (s, 3H, CO_2_Me), 2.43 (s, 3H, Me′), 2.09, 2.08, 2.09 (3× s, 9H, 3× OAc). ^13^C-NMR (75 MHz, CDCl_3_): δ = 170.02, 169.37, 169.19 (3× OAc), 166.62 (C-6), 160.79 (C-2′), 159.02 (C-7′), 154.74, 152.22, 125.80, 115.65, 113.89, 113.22 (C-3′, C-4′, C-5′, C-6′, C-4a′, C-8a′), 104.15 (C-8′), 98.26 (C-1), 72.56 (C-5), 71.61, 70.79, 68.88 (C-2, C-3, C-4), 53.09 (CO_2_Me), 20.60, 20.60, 20.50 (3× OAc), 18.69 (C-Me′). HRMS (ESI): *m*/*z* [M + Na]^+^ calcd for C_23_H_24_NaO_12_: 515.1160; found: 515.1164. ^1^H- and ^13^C-NMR, and HRMS spectrograms are seen in the Supplement Materials.

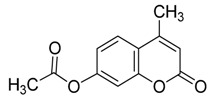


*4-Methylumbelliferyl acetate* (**5**) Yellowish white powder; mp: 148–150 °C; *R_f_* = 0.35 (PE–AcOEt, 3:2). ^1^H-NMR (300 MHz, CDCl_3_): δ = 7.54 (d, *J*_5,6_ = 8.7 Hz, 1H, H-5), 7.04 (d, *J*_6,8_ = 2.2 Hz, 1H, H-8), 7.01 (dd, *J*_5,6_ = 8.7, *J*_6,8_ = 2.2 Hz, 1H, H-6), 6.20 (d, *J*_3,Me_ = 1.2 Hz, 1H, H-3), 2.36 (d, *J*_3,Me_ = 1.2 Hz, 3H, H-Me), 2.27 (s, 3H, OAc). ^13^C-NMR (75 MHz, CDCl_3_): δ = 168.78 (OAc), 160.51 (C-2), 154.16 (C-7), 153.04, 151.95, 125.41, 118.12, 117.85, 114.52, 110.47 (C-3, C-4, C-5, C-6, C-4a, C-8a), 21.13 (C-Me), 18.74 (C-Me). HRMS (ESI): *m*/*z* [M + Na]^+^ calcd for C_12_H_10_NaO_4_: 241.0471; found: 241.0477. ^1^H- and ^13^C-NMR, and HRMS spectrograms are seen in the Supplement Materials.

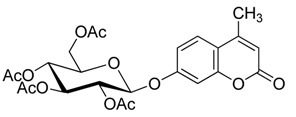


*4′-Methylumbelliferyl 2,3,4,6-tetra-O-acetyl-β-d-glucopyranoside* (**3b_1_**) Compound **3b_1_** was prepared according to the general procedure for the glycosylation step using 4-MU (176 mg, 1.0 mmol), glycosyl acetate (781 mg, 2.0 mmol, 2.0 equiv.), dry ClCH_2_CH_2_Cl (3 mL), corresponding molar equivalents of pyridine and BF_3_·OEt_2_. The mixture was stirred for 5 h at 60 °C (as shown in Table 3). The crude product was purified by flash column chromatography. White powder; mp: 142–143 °C; ${\left[\alpha \right]}_{D}^{19}$ −35° (*c* = 0.40, CHCl_3_); *R_f_* = 0.27 (PE–AcOEt, 3:2); {lit. [21] mp: 144 °C, ${\left[\alpha \right]}_{D}^{20}$ −40 ± 2° (*c* = 0.5, CHCl_3_); lit. [25] mp: 144–145 °C, ${\left[\alpha \right]}_{D}^{20}$ −36° (*c* = 1, CHCl_3_), *R_f_* = 0.26 (hexanes–AcOEt, 1:1); lit. [27] mp: 143–144 °C, ${\left[\alpha \right]}_{D}^{20}$ −39° (*c* = 0.635, CHCl_3_), *R_f_* = 0.18 (CHCl_3_–acetone, 19:1)}. ^1^H-NMR (300 MHz, CDCl_3_): δ = 7.54 (d, *J*_5′,6′_ = 8.7 Hz, 1H, H-5′), 6.98–6.93 (m, 2H, H-6′, H-8′), 6.22 (d, *J*_3′,Me′_ = 0.9 Hz, 1H, H-3′), 5.38–5.30 (m, 2H, H-1, H-3), 5.22–5.16 (m, 2H, H-2, H-4), 4.32 (dd, *J*_6a,6b_ = 12.1, *J*_5,6a_ = 5.5 Hz, 1H, H-6a), 4.20 (dd, *J*_6a,6b_ = 12.1, *J*_5,6b_ = 1.7 Hz, 1H, H-6b), 3.93 (ddd, *J*_4,5_ = 7.4, *J*_5,6a_ = 5.5, *J*_5,6b_ = 1.7 Hz, 1H, H-5), 2.43 (d, *J*_3′Me′_ = 0.9 Hz, 3H, Me′), 2.14, 2.09, 2.09, 2.06 (4× s, 12H, 4× OAc). ^13^C-NMR (75 MHz, CDCl_3_): δ = 170.61, 170.17, 169.41, 169.26 (4× OAc), 160.74 (C-2′), 159.17 (C-7′), 154.82, 152.19, 125.74, 115.51, 113.98, 113.19 (C-3′, C-4′, C-5′, C-6′, C-4a′, C-8a′), 103.93 (C-8′), 98.33 (C-1), 72.55, 72.40, 70.92, 68.06, 61.80 (C-2, C-3, C-4, C-5, C-6), 20.71, 20.62, 20.59, 20.59 (4× OAc), 18.69 (C-Me′). HRMS (ESI): *m*/*z* [M + Na]^+^ calcd for C_24_H_26_NaO_12_: 529.1316; found: 529.1323. ^1^H- and ^13^C-NMR, and HRMS spectrograms are seen in the Supplement Materials.

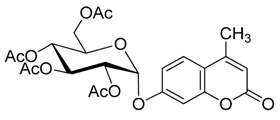


*4′-Methylumbelliferyl 2,3,4,6-tetra-O-acetyl-α-d-glucopyranoside* (**3b_2_**) White powder; mp: 132–133 °C; ${\left[\alpha \right]}_{D}^{22}$ +202° (*c* = 0.30, CHCl_3_); *R_f_* = 0.31 (PE–AcOEt, 3:2); {lit. [27] mp: 131–132 °C, ${\left[\alpha \right]}_{D}^{20}$ +200° (*c* = 0.5, CHCl_3_), *R_f_* = 0.27 (CHCl_3_–acetone, 19:1)}. ^1^H-NMR (300 MHz, CDCl_3_): δ = 7.56 (d, *J*_5′,6′_ = 8.7 Hz, 1H, H-5′), 7.11 (d, *J*_6′,8′_ = 2.3 Hz, 1H, H-8′), 7.06 (dd, *J*_5′,6′_ = 8.7, *J*_6′,8′_ = 2.4 Hz, 1H, H-6′), 6.22 (s, 1H, H-3′), 5.82 (d, *J*_1,2_ = 3.3 Hz, 1H, H-1), 5.71 (t, *J*_3,4_ = 9.9 Hz, 1H, H-3), 5.18 (t, *J*_3,4_ = 9.9 Hz, 1H, H-4), 5.08 (dd, *J*_2,3_ = 10.2, *J*_1,2_ = 3.6 Hz, 1H, H-2), 4.28 (dd, *J* = 12.6, 4.8 Hz, 1H, H-6a), 4.13–4.01 (m, 2H, H-5, H-6b), 2.43 (s, 3H, Me′), 2.08 (4s, 12H, 4× OAc). ^13^C-NMR (75 MHz, CDCl_3_): δ = 170.51, 170.15, 170.11, 169.52 (4× OAc), 160.75 (C-2′), 158.61 (C-7′), 154.84, 152.10, 125.85, 115.53, 113.32, 113.24 (C-3′, C-4′, C-5′, C-6′, C-4a′, C-8a′), 104.58 (C-8′), 94.37 (C-1), 70.18, 69.79, 68.40, 68.09, 61.45 (C-2, C-3, C-4, C-5, C-6), 20.68, 20.62, 20.61, 20.57 (4× OAc), 18.67 (C-Me′). HRMS (ESI): *m*/*z* [M + Na]^+^ calcd for C_24_H_26_NaO_12_: 529.1316; found: 529.1321. ^1^H- and ^13^C-NMR, and HRMS spectrograms are seen in the Supplement Materials.

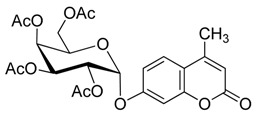


*4′-Methylumbelliferyl 2,3,4,6-tetra-O-acetyl-α-d-galactopyranoside* (**3c**) Compound **3c** was prepared according to the general procedure for the glycosylation step using 4-MU (352 mg, 2.0 mmol), β-d-galactose pentaacetate (1561 mg, 4.0 mmol, 2.0 equiv.), dry ClCH_2_CH_2_Cl (6 mL), TEA (700 μL, 5.0 mmol, 2.5 equiv.) and BF_3_·OEt_2_ (3218 μL, 25 mmol, 12.5 equiv.). The mixture was stirred for 5 h at 60 °C (as shown in Table 3). The crude product was purified by flash column chromatography. White powder; yield: 548 mg (54%); mp: 176–180 °C; ${\left[\alpha \right]}_{D}^{19}$ +209° (*c* = 0.40, CHCl_3_); *R_f_* = 0.32 (PE–AcOEt, 3:2); {lit. [27] mp: 186.5–188.5 °C, ${\left[\alpha \right]}_{D}^{20}$ +210° (*c* = 0.281, CHCl_3_), *R_f_* = 0.43 (CHCl_3_–acetone, 9:1)}. ^1^H-NMR (500 MHz, CDCl_3_): δ = 7.53 (d, *J*_5′,6′_ = 8.7 Hz, 1H, H-5′), 7.06 (s, 1H, H-8′), 7.00 (d, *J* = 8.7 Hz, 1H, H-6′), 6.18 (s, 1H, H-3′), 5.83 (d, *J*_1,2_ = 3.0 Hz, 1H, H-1), 5.55 (dd, *J*_2,3_ = 11.0 Hz, *J*_1,2_ = 3.0 Hz, 1H, H-2), 5.52 (d, *J*_3,4_ = 3.2 Hz, 1H, H-4), 5.29 (dd, *J*_2,3_ = 10.8 Hz, *J*_3,4_ = 3.2 Hz, 1H, H-3), 4.28 (t, *J*_5,6a_ = 6.3 Hz, 1H, H-5), 4.14–4.01 (m, 2H, H-6a, H-6b), 2.40 (s, 3H, Me′), 2.17 (s, 3H, OAc), 2.07 (s, 3H, OAc), 2.02 (s, 3H, OAc), 1.94 (s, 3H, OAc). ^13^C-NMR (75 MHz, CDCl_3_): δ = 170.36, 170.31, 170.14, 170.06 (4× OAc), 160.81 (C-2′), 158.90 (C-7′), 154.86, 152.13, 125.79, 115.45, 113.54, 113.20 (C-3′, C-4′, C-5′, C-6′, C-4a′, C-8a′), 104.59 (C-8′), 95.04 (C-1), 67.60, 67.52, 67.51, 67.32, 61.30 (C-2, C-3, C-4, C-5, C-6), 20.73, 20.68, 20.64, 20.57 (4× OAc), 18.70 (C-Me′). HRMS (ESI): *m*/*z* [M + Na]^+^ calcd for C_24_H_26_NaO_12_: 529.1316; found: 529.1323. ^1^H- and ^13^C-NMR, and HRMS spectrograms are seen in the Supplement Materials.

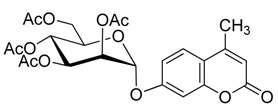


*4′-Methylumbelliferyl 2,3,4,6-tetra-O-acetyl-α-d-mannopyranoside* (**3d**) Compound **3d** was prepared according to the general procedure for the glycosylation step using 4-MU (1057 mg, 6.0 mmol), β-d-mannose pentaacetate (1171 mg, 3.0 mmol, 0.5 equiv.), dry ClCH_2_CH_2_Cl (9 mL), pyridine (728 μL, 9.0 mmol, 1.5 equiv.) and BF_3_·OEt_2_ (5792 μL, 45 mmol, 7.5 equiv.). The mixture was stirred for 5 h at 60 °C (as shown in Table 3). The crude product was purified by three recrystallizations in EtOH. White and slightly yellow acicular crystals; yield: 899 mg (59%); mp: 161.5–163.5 °C; ${\left[\alpha \right]}_{D}^{20}$ +104° (*c* = 0.35, CHCl3); *R_f_* = 0.28 (PE–AcOEt, 3:2); {lit. [27] mp 161–163 °C, ${\left[\alpha \right]}_{D}^{22}$ +106.0° (*c* = 0.321, CHCl_3_), *R_f_* = 0.54 (CH_2_Cl_2_–acetone, 23:2); lit. [28] mp: 160–161 °C, ${\left[\alpha \right]}_{D}^{22}$ +136.0° (c = 2, CHCl_3_)}. ^1^H-NMR (300 MHz, CDCl_3_): δ = 7.56 (d, *J*_5′,6′_ = 8.7 Hz, 1H, H-5′), 7.13 (d, *J*_6′,8′_ = 2.1 Hz, 1H, H-8′), 7.04 (dd, *J*_5′,6′_ = 8.8, *J*_6′,8′_ = 2.4 Hz, 1H, H-6′), 6.21 (s, 1H, H-3′), 5.60–5.36 (m, 4H, H-1, H-2, H-3, H-4), 4.30 (dd, *J* = 12.6, 6.0 Hz, 1H, H-6a), 4.09–4.03 (m, 2H, H-6b, H-5), 2.43 (s, 3H, Me′), 2.24 (s, 3H, OAc), 2.07, 2.06, 2.06 (3× s, 9H, 3× OAc). ^13^C-NMR (75 MHz, CDCl_3_): δ = 170.48, 169.94, 169.93, 169.63 (4× OAc), 160.73 (C-2′), 158.10 (C-7′), 154.81, 152.14, 125.81, 115.44, 113.40, 113.19 (C-3′, C-4′, C-5′, C-6′, C-4a′, C-8a′), 104.29 (C-8′), 95.84 (C-1), 69.49, 68.99, 68.62, 65.68, 61.98 (C-2, C-3, C-4, C-5, C-6), 20.85, 20.67, 20.65, 20.62 (4× OAc), 18.66 (C-Me′). HRMS (ESI): *m*/*z* [M + Na]^+^ calcd for C_24_H_26_NaO_12_: 529.1316; found: 529.1322. ^1^H- and ^13^C-NMR, and HRMS spectrograms are seen in the Supplement Materials.

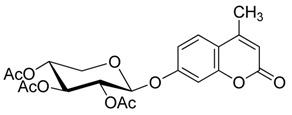


*4′-Methylumbelliferyl 2,3,4-tri-O-acetyl-β-D-xylopyranoside* (**3e**) Compound **3e** was prepared according to the general procedure for the glycosylation step using 4-MU (352 mg, 2.0 mmol), β-d-xylose tetraacetate (1273 mg, 4.0 mmol, 2.0 equiv.), dry CH_2_Cl_2_ (6 mL), TEA (700 μL, 5.0 mmol, 2.5 equiv.) and BF_3_·OEt_2_(3218 μL, 25.0 mmol, 12.5 equiv.). The mixture was stirred for 5 h at 20–21 °C (as shown in Table 3). The crude product was purified by flash column chromatography. White powder; yield: 633 mg (73%); mp: 148–153 °C; ${\left[\alpha \right]}_{D}^{20}$ −49° (c = 1.00, CHCl_3_). *R_f_* = 0.33 (PE–AcOEt, 3:2); {lit. [43] mp: 158 °C, ${\left[\alpha \right]}_{D}^{22}$ −45° (*c* = 2, CHCl_3_), *R_f_* = 0.65 (AcOEt–benzene, 3:7)}. ^1^H-NMR (300 MHz, CDCl_3_): δ = 7.53 (d, *J*_5′,6′_ = 8.7 Hz, 1H, H-5′), 6.97–6.92 (m, 2H, H-6′, H-8′), 6.19 (d, *J*_3″,Me′_ = 1.2 Hz, 1H, H-3′), 5.31–5.17 (m, 3H, H-1, H-2, H-3), 5.02 (td, *J*_4,5b_ = 7.1, *J*_4,5a_ = 4.5 Hz, 1H, H-4), 4.23 (dd, *J*_5a,5b_ = 12.3, *J*_4,5a_ = 4.5 Hz, 1H, H-5a), 3.60 (dd, *J*_5a,5b_ = 12.3, *J*_4,5b_ = 7.2 Hz, 1H, H-5b), 2.41 (d, *J*_3″,Me′_ = 1.2 Hz, 3H, Me′), 2.11, 2.11, 2.11 (3× s, 9H, 3× OAc). ^13^C-NMR (75 MHz, CDCl_3_): δ = 169.84, 169.83, 169.36 (3× OAc), 160.88 (C-2′), 158.99 (C-7′), 154.83, 152.22, 125.76, 115.36, 113.59, 113.08 (C-3′, C-4′, C-5′, C-6v, C-4a′, C-8a′), 104.18 (C-8′), 97.77 (C-1), 70.15, 69.59, 68.09, 61.81 (C-2, C-3, C-4, C-5), 20.80, 20.75, 20.71 (3× OAc), 18.70 (C-Me′). HRMS (ESI): *m*/*z* [M + Na]^+^ calcd for C_21_H_22_NaO_10_: 457.1105; found: 457.1112. ^1^H- and ^13^C-NMR, and HRMS spectrograms are seen in the Supplement Materials.
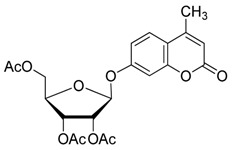


*4′-Methylumbelliferyl 2,3,5-tri-O-acetyl-β-d-ribofuranoside* (**3f**) Compound **3f** was prepared according to the general procedure for the glycosylation step using 4-MU (352 mg, 2.0 mmol), β-d-ribose tetraacetate (1273 mg, 4.0 mmol, 2.0 equiv), dry CH_2_Cl_2_ (6 mL), TEA (700 μL, 5.0 mmol, 2.5 equiv.) and BF_3_·OEt_2_ (3218 μL, 25 mmol, 12.5 equiv.). The mixture was stirred for 3 h at 20–21 °C (as shown in Table 3). The crude product was purified by flash column chromatography. Colorless syrup; yield: 817 mg (94%); *R_f_* = 0.31 (PE–AcOEt, 3:2). Little purer colorless syrup obtained by HPLC separation was analyzed by NMR spectrometers and HRMS. ^1^H-NMR (300 MHz, CDCl_3_): δ = 7.45 (d, *J* = 8.7 Hz, 1H, H-5′), 6.92–6.85 (m, 2H, H-6′, H-8′), 6.10 (s, 1H, H-3′), 5.63 (s, 1H, H-1), 5.47–5.42 (m, 2H, H-2, H-3), 4.38 (dd, *J* = 9.0, 4.8 Hz, 1H, H-4), 4.31 (dd, *J* = 12.0, 3.6 Hz, 1H, H-5a), 3.98 (dd, *J* = 12.0, 4.7 Hz, 1H, H-5b), 2.33 (s, 3H, Me′), 2.10, 2.04, 1.87 (3× s, 9H, 3× OAc). ^13^C-NMR (75 MHz, CDCl_3_): δ = 170.47, 169.75, 169.57 (3× OAc), 161.04 (C-2′), 158.71 (C-7′), 154.80, 152.36, 125.71, 114.96, 113.27, 112.84 (C-3′, C-4′, C-5′, C-6′, C-4a′, C-8a′), 103.95 (C-8′), 102.90 (C-1), 79.57, 74.78, 70.71, 63.22 (C-2, C-3, C-4, C-5), 20.64, 20.58, 20.51 (3× OAc), 18.67 (C-Me′). HRMS (ESI): *m*/*z* [M + Na]^+^ calcd for C_21_H_22_NaO_10_: 457.1105; found: 457.1111. ^1^H- and ^13^C-NMR, and HRMS spectrograms are seen in the Supplement Materials.

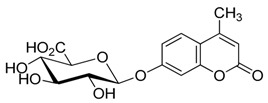


*4-Methylumbelliferyl β-d-glucopyranosiduronic acid* (**4a**) To a stirred suspension of Ba(OH)_2_·H_2_O (142 mg, 0.75mmol, 3 equiv) in MeOH (6 mL) and distilled water (2.4 mL) in an ice-water bath under an argon atmosphere was added compound **3a** (123 mg, 0.25 mmol). The mixture was stirred for 4 h in an ice-water bath, then glacial acetic acid was added carefully into to adjust the pH value to 7.5–8.0. The straw yellow solid precipitation was filtered off, washed with MeOH, then further purified by recrystallization using MeOH, and dried. The obtained barium salt product (105 mg) was added to fresh MeOH (5 mL) in an ice-water bath again, acidified with H_2_CO_4_·2H_2_O (20 mg, 0.16 mmol). After stirring for 0.5 h, the mixture was filtered. The filter residue was washed with 2–3 mL MeOH, and the merged filtrate was evaporated to a syrup under reduced pressure. The final product was crystallized from anhydrous ether, washed with a small account of cold acetone, and dried. White powder; yield: 42 mg (47%); mp: 140–144 °C; ${\left[\alpha \right]}_{D}^{21}$ −114° (*c* = 0.20, H_2_O); {lit. [27] mp: 139–145 °C, ${\left[\alpha \right]}_{D}^{20}$ −108° (*c* = 0.25, pyridine); lit. [29] mp: 139–140 °C, ${\left[\alpha \right]}_{D}^{20}$ −114° (*c* = 0.25, H_2_O); lit. [32] mp: 139–140 °C, ${\left[\alpha \right]}_{D}^{22}$ −119° (*c* = 0.25, H_2_O); lit. [33] mp: 140–143 °C, ${\left[\alpha \right]}_{D}^{20}$ −116° (*c* = 0.25, H_2_O)}. ^1^H-NMR (500 MHz, DMSO-*d*_6_): δ = 7.72 (d, *J*_5′,6′_ = 8.5 Hz, 1H, H-5′), 7.16–7.03 (m, 2H, H-6′, H-8′), 6.26 (s, 1H, H-3v), 5.23 (d, *J*_1,2_ = 6.0 Hz, 1H, H-1), 4.00 (d, *J*_4,5_ = 9.0 Hz, 1H, H-5), 3.39–3.29 (m, 3H, H-2, H-3, H-4), 2.40 (s, 3H, Me). ^13^C-NMR (75 MHz, DMSO-*d*_6_): δ = 170.17 (C-6), 160.01 (C-2′), 159.62 (C-7′), 154.33, 153.26, 126.49, 114.17, 113.18, 111.75 (C-3′, C-4′, C-5′, C-6′, C-4a′, C-8a′), 102.98 (C-8′), 99.25 (C-1), 75.70 (C-5), 75.19, 72.77, 71.26 (C-2, C-3, C-4), 18.09 (C-Me′). HRMS (ESI): *m*/*z* [M + Na]^+^ calcd for C_16_H_16_NaO_9_: 375.0687; found: 375.0692. ^1^H- and ^13^C-NMR, and HRMS spectrograms are seen in the Supplement Materials.

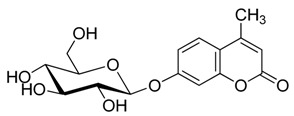


*4-Methylumbelliferyl β-d-glucopyranoside* (**4b_1_**) Compound **3b_1_** (100 mg, 0.20 mmol) was *O*-deacetylated for 1 h at room temperature with KOH (1.0 M in anhydrous MeOH; 40 μL, 0.2 equiv.) in anhydrous MeOH (3 mL). Concentration of the solution gave a crystalline material that was filtered off, washed with ethanol until the washings were no longer fluorescent, and dried. White crystal; yield: 56 mg (83%); mp: 209–211 °C; ${\left[\alpha \right]}_{D}^{20}$ −85° (*c* = 0.30, H_2_O); {lit. [21] mp: 211 °C, ${\left[\alpha \right]}_{D}^{20}$ −89.5° (*c* = 0.5, H_2_O); lit. [27] mp: 211–213 °C, ${\left[\alpha \right]}_{D}^{20}$ −68° (*c* = 0.5, pyridine)}. ^1^H-NMR (300 MHz, DMSO-*d*_6_): δ = 7.71 (d, *J*_5′,6′_ = 9.3 Hz, 1H, H-5′), 7.08–7.00 (m, 2H, H-6′, H-8′), 6.26 (d, *J*_3′,Me′_ = 0.9 Hz, 1H, H-3′), 5.42 (d, *J*_1,2_ = 4.8 Hz, 1H, H-1), 5.16 (d, *J*_1,2_ = 4.5 Hz, 1H, H-2), 5.09–5.03 (m, 2H), 4.61 (t, *J* = 5.4 Hz, 1H), 3.73–3.66 (m, 1H), 3.50–3.40 (m, 2H), 3.31–3.23(m, 3H), 3.20–3.13 (m, 1H), 2.41 (s, 3H, Me′). ^13^C-NMR (75 MHz, DMSO-*d*_6_): δ = 160.10 (C-2′), 160.08 (C-7′), 154.35, 153.30, 126.37, 114.02, 113.34, 111.65 (C-3′, C-4′, C-5′, C-6′, C-4a′, C-8a′), 103.15 (C-8′), 99.93 (C-1), 77.10, 76.44, 73.09, 69.58, 60.60 (C-2, C-3, C-4, C-5, C-6), 18.11 (C-Me′). HRMS (ESI): *m*/*z* [M + Na]^+^ calcd for C_16_H_18_NaO_8_: 361.0894; found: 361.0900. ^1^H- and ^13^C-NMR, and HRMS spectrograms are seen in the Supplement Materials.

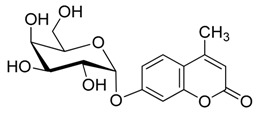


*4-Methylumbelliferyl α-d-galactopyranoside* (**4c**) Compound **3c** (400 mg, 0.79 mmol) was *O*-deacetylated for 15 min at room temperature with KOH (1.0 M in anhydrous MeOH; 237 μL, 0.3 equiv.) in dry tetrahydrofuran (8 mL) and anhydrous MeOH (4 mL). The white solid precipitate was filtered off, washed with ethanol until the washings were no longer fluorescent, and dried. White powder; yield: 214 mg (80%); mp: 222–223 °C; ${\left[\alpha \right]}_{D}^{20}$ +238° (*c* = 0.30, H_2_O); {lit. [27] mp: 212–217 °C; ${\left[\alpha \right]}_{D}^{20}$ +135° (*c* = 1.37, pyridine)}. ^1^H-NMR (500 MHz, DMSO-*d*_6_): δ = 7.70 (d, *J*_5′,6′_ = 9.0 Hz, 1H, H-5′), 7.09–7.07 (m, 2H, H-6′, H-8′), 6.24 (d, *J*_3′,Me′_ = 1.0 Hz, 1H, H-3′), 5.60 (d, *J*_1,2_ = 3.0 Hz, 1H, H-1), 4.99 (d, *J* = 6.5 Hz, 1H), 4.79 (d, *J* = 5.0 Hz, 1H), 4.61 (d, *J* = 4.5 Hz, 1H), 4.53 (t, *J* = 5.7 Hz, 1H), 3.83–3.74 (m, 3H), 3.64 (t, *J* = 5.7 Hz, 1H, H-5), 3.52 (dt, *J* = 11.0, 6.2 Hz, 1H, H-6a), 3.38 (dt, *J* = 11.0, 6.2 Hz, 1H, H-6b), 2.40 (s, 3H, H-Me′). ^13^C-NMR (75 MHz, DMSO-*d*_6_): δ = 160.07 (C-2′), 160.07 (C-7′), 154.30, 153.32, 126.32, 113.92, 113.82, 111.56 (C-3′, C-4′, C-5′, C-6′, C-4a′, C-8a′), 103.65 (C-8′), 98.07 (C-1), 72.68, 69.30, 68.42, 67.71, 60.20 (C-2, C-3, C-4, C-5, C-6), 18.09 (C-Me′). HRMS (ESI): *m*/*z* [M + Na]^+^ calcd for C_16_H_18_NaO_8_: 361.0894; found: 361.0897. ^1^H- and ^13^C-NMR, and HRMS spectrograms are seen in the Supplement Materials.
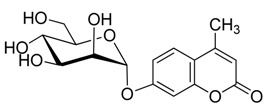


*4-Methylumbelliferyl α-d-mannopyranoside* (**4d**) Compound **3d** (200 mg, 0.39 mmol) was *O*-deacetylated for 45 min at room temperature with KOH (1.0 M in anhydrous MeOH; 119 μL, 0.3 equiv.) in anhydrous MeOH (2 mL). The beige solid precipitate was filtered off, washed with ethanol until the washings were no longer fluorescent, and dried. Beige powder; yield: 106 mg (80%); mp: 213–214 °C; ${\left[\alpha \right]}_{D}^{20}$ +154° (*c* = 0.35, H_2_O); {lit. [27] mp: 220–224 °C, ${\left[\alpha \right]}_{D}^{20}$ +157° (*c* = 0.4, H_2_O); lit. [28] mp: 222–225 °C, ${\left[\alpha \right]}_{D}^{22}$ +178.2° (*c* = 2, MeOH)}. ^1^H-NMR (500 MHz, DMSO-*d*_6_): δ = 7.69 (d, *J*_5′,6′_ = 9.0 Hz, 1H, H-5′), 7.11–7.07 (m, 2H, H-6′, H-8′), 6.24 (s, 1H, H-3′), 5.53 (s, 1H, H-1), 5.13 (d, *J* = 2.0 Hz, 1H), 4.90–4.84 (m, 2H), 4.51 (t, *J* = 5.5 Hz, 1H), 3.85 (s, 1H), 3.68 (t, *J* = 4.0 Hz,1H), 3.60–3.57 (m, 1H), 3.52–3.42 (m, 2H), 3.32 (s, 1H), 2.40 (s, 3H, H-Me′). ^13^C-NMR (75 MHz, DMSO-*d*_6_): δ = 160.04 (C-2′), 159.07 (C-7′), 154.30, 153.29, 126.41, 114.08, 113.64, 111.68 (C-3′, C-4′, C-5′, C-6′, C-4a′, C-8a′), 103.61 (C-8′), 98.72 (C-1), 75.31, 70.51, 69.74, 66.53, 60.91 (C-2, C-3, C-4, C-5, C-6), 18.09 (C-Me′). HRMS (ESI): *m*/*z* [M + Na]^+^ calcd for C_16_H_18_NaO_8_: 361.0894; found: 361.0900. ^1^H- and ^13^C-NMR, and HRMS spectrograms are seen in the Supplement Materials.

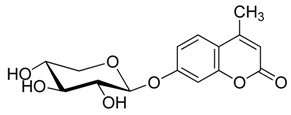


*4-Methylumbelliferyl β-d-xylopyranoside* (**4e**) Compound **3e** (200 mg, 0.46 mmol) was *O*-deacetylated for 25 min at room temperature with KOH (1.0 M in anhydrous MeOH; 138 μL, 0.3 equiv.) in anhydrous MeOH (5 mL). The white solid precipitate was filtered off, washed with ethanol until the washings were no longer fluorescent, and dried. White powder; yield: 112 mg (79%); mp: 217–218 °C; ${\left[\alpha \right]}_{D}^{20}$ −65° (*c* = 0.10, H_2_O); {lit. [43] mp: 213–214 °C, ${\left[\alpha \right]}_{D}^{22}$ −42° (*c* = 0.10, H_2_O)}. ^1^H-NMR (300 MHz, DMSO-*d*_6_): δ = 7.70 (d, *J*_5′,6′_ = 9.3 Hz, 1H, H-5′), 7.04–7.00 (m, 2H, H-6′, H-8′), 6.25 (s, 1H, H-3′), 5.45 (d, *J*_1,2_ = 4.8 Hz, 1H, H-1), 5.19 (t, *J* =2.3 Hz, 1H), 5.12 (d, *J* = 4.2 Hz, 1H), 5.06 (t, *J* = 3.6 Hz, 1H), 3.80–3.71 (m, 1H), 3.43–3.33 (m, 2H), 3.31–3.21 (m, 2H), 2.40 (s, 3H, Me′). ^13^C-NMR (75 MHz, DMSO-*d*_6_): δ = 160.03 (C-2′), 159.83 (C-7′), 154.33, 153.27, 126.44, 114.07, 113.26, 111.71 (C-3′, C-4′, C-5′, C-6′, C-4a′, C-8a′), 103.06 (C-8′), 100.24 (C-1), 76.27, 72.89, 69.21, 65.68 (C-2, C-3, C-4, C-5), 18.09 (C-Me′). HRMS (ESI): *m*/*z* [M + Na]^+^ calcd for C_15_H_16_NaO_7_: 331.0788; found: 331.0793. ^1^H- and ^13^C-NMR, and HRMS spectrograms are seen in the Supplement Materials.

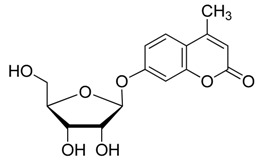


*4-Methylumbelliferyl β-d-ribofuranoside* (**4f**) Compound **3f** (200 mg, 0.46 mmol) was *O*-deacetylated for 25 min at room temperature with KOH (1.0 M in anhydrous MeOH; 138 μL, 0.3 equiv.) in anhydrous MeOH (5 mL). The white solid precipitate was filtered off, washed with ethanol until the washings were no longer fluorescent, and dried. White powder; yield: 112 mg (79%); mp: 167–169 °C; ${\left[\alpha \right]}_{D}^{20}$ −145° (*c* = 0.15, H_2_O). ^1^H-NMR (300 MHz, DMSO-*d*_6_): δ = 7.67 (d, *J*_5′,6′_ = 8.7 Hz, 1H, H-5′), 7.00–6.96 (m, 2H, H-6′, H-8′), 6.22 (d, *J*_3′,Me′_ = 1.2 Hz, 1H, H-3′), 5.60 (s, 1H, H-1), 5.43 (d, *J* = 4.5 Hz, 1H), 5.08 (d, *J* = 6.3 Hz, 1H), 4.71 (t, *J* = 5.4 Hz, 1H), 4.08–4.00 (m, 2H), 3.93 (td, *J* = 6.0, 3.6 Hz, 1H), 3.55 (ddd, *J* = 11.7, 5.4, 3.6 Hz, 1H), 3.38–3.29 (m, 1H), 2.38 (d, *J*_3′,Me′_ = 0.9 Hz, 3H, Me′). ^13^C-NMR (75 MHz, DMSO-*d*_6_): δ = 160.02 (C-2′), 159.31 (C-7′), 154.26, 153.25, 126.41, 113.81, 113.32, 111.54 (C-3′, C-4′, C-5′, C-6′, C-4a′, C-8a′), 105.06 (C-8′), 103.26 (C-1), 84.82, 74.47, 70.41, 62.52 (C-2, C-3, C-4, C-5), 18.07 (C-Me′). HRMS (ESI): *m*/*z* [M + Na]^+^ calcd for C_15_H_16_NaO_7_: 331.0788; found: 331.0791. ^1^H- and ^13^C-NMR, and HRMS spectrograms are seen in the Supplement Materials.

## 4. Conclusions

We have developed a useful method for the synthesis of 4-methylumbelliferyl glycosides. Notable benefits, such as the easy availability of glycosyl acetates, α-/β-stereoselectivity, moderate to excellent yields (51%–94%), and easy operation, make this method a practical approach for obtaining 4-methylumbelliferyl glycosides. These compounds have been often applied to detect the activities of glycosidases from different sources, particularly microorganisms. For example, 4-methy-lumbelliferyl β-d-glucopyranosiduronic acid and 4-methylumbelliferyl β-d-glucopyranoside are widely used for detection of *Escherichia coli* and enterococci, respectively, and 4-methylumbelliferyl α-d-galactopyranoside can be used to help differentiate between streptococci and enterococci [4,18]. The search for novel glycosidase substrates with better performance or new functions is also of continuing interesting for biochemists. Herein we hope that this improved Helferich method will be beneficial for this objective.

## Supplementary Materials

Supplementary materials can be accessed at: http://www.mdpi.com/1420-3049/20/12/19789/s1.

## References

[1] Yang X., Wu Q., Zhang J., Huang J., Chen L., Liu S., Yu S., Cai S. (2015). Prevalence, enumeration, and characterization of Salmonella isolated from aquatic food products from retail markets in China. Food Control.

[2] Zhang S., Zhu X., Wu Q., Zhang J., Xu X., Li H. (2015). Prevalence and characterization of *Escherichia coli* O157 and O157:H7 in retail fresh raw meat in South China. Ann. Microbiol..

[3] Food Safety in Fact Sheets. www.who.int/mediacentre/factsheets/fs399/en/.

[4] Manafi M., Kneifel W., Bascomb S. (1991). Fluorogenic and chromogenic substrates used in bacterial diagnostics. Microbiol. Mol. Biol. Rev..

[5] Manafi M. (1996). Fluorogenic and chromogenic enzyme substrates in culture media and identification tests. Int. J. Food Microbiol..

[6] Manafi M. (2000). New developments in chromogenic and fluorogenic culture media. Int. J. Food Microbiol..

[7] Jokerst J.C., Adkins J.A., Bisha B., Mentele M.M., Goodridge L.D., Henry C.S. (2012). Development of a paper-based analytical device for colorimetric detection of select foodborne pathogens. Anal. Chem..

[8] Wu Q., Wei X., Zhang J., Guo W. (2013). Research progress of syntheses and applications in detecting microorganisms of indoxyl chromogenic substrates. Chemistry.

[9] Orenga S., James A.L., Manafi M., Perry J.D., Pincus D.H. (2009). Enzymatic substrates in microbiology. J. Microbiol. Methods.

[10] Nilssen Ø., Stensland H.M.F.R., Malm D. (2011). Clinical utility gene card for: α-Mannosidosis. Eur. J. Hum. Genet..

[11] Marsden D., Levy H. (2010). Newborn screening of lysosomal storage disorders. Clin. Chem..

[12] Nakamura K., Hattori K., Endo F. (2011). Newborn screening for lysosomal storage disorders. Am. J. Med. Genet. Part C.

[13] Oemardien L.F., Boer A.M., Ruijter G.J., van der Ploeg A.T., de Klerk J.B., Reuser A.J., Verheijen F.W. (2011). Hemoglobin precipitation greatly improves 4-methylumbelliferone-based diagnostic assays for lysosomal storage diseases in dried blood spots. Mol. Genet. Metab..

[14] O'Riordan N., Kane M., Joshi L., Hickey R.M. (2014). Glycosidase activities in bovine milk over lactation. Int. Dairy J..

[15] Oguri S., Nakaoka A., Amano Y., Ito Y. (2012). Application of glycosidase activity as a marker for characterizing and identifying vegetables. J. Food Compos. Anal..

[16] Carrasco L.C., Romar R., Avilés M., Gadea J., Coy P. (2008). Determination of glycosidase activity in porcine oviductal fluid at the different phases of the estrous cycle. Reproduction.

[17] Gold H., Munneke S., Dinkelaar J., Overkleeft H.S., Aerts J.M., Codée J.D., van der Marel G.A. (2011). A practical synthesis of capped 4-methylumbelliferyl hyaluronan disaccharides and tetrasaccharides as potential hyaluronidase substrates. Carbohydr. Res..

[18] Perry J.D., James A.L., Morris K.A., Oliver M., Chilvers K.F., Reed R.H., Gould F.K. (2006). Evaluation of novel fluorogenic substrates for the detection of glycosidases in *Escherichia coli* and enterococci. J. Appl. Microbiol..

[19] Jensen K.J. (2002). *O*-Glycosylations under neutral or basic conditions. J. Chem. Soc. Perkin Trans. 1.

[20] Zhu J., Dong J., Yang Z. (1997). Synthesis and application of Mugal, an enzyme-substrate rapid determination reagent. Huaxue Shiji.

[21] Robinson D. (1956). The fluorimetric determination of β-glucosidase: Its occurrence in the tissues of animals, including insects. Biochem. J..

[22] Leaback D.H., Walker P.G. (1961). Studies on glucosaminidase. 4. The fluorimetric assay of *N*-acetyl-β-glucosaminidase. Biochem. J..

[23] Strachan R., Wood J., Hirschmann R. (1962). Synthesis and properties of 4-methyl-2-*oxo*-1,2-benzopyran-7-yl β-d-galactoside (galactoside of 4-methylumbelliferone). J. Org. Chem..

[24] Ma Y., Wu Q., Zhang J., Guo W., Wei X. (2014). Synthesis and application of Mugal which is the specific fluorescent substrate for detection of coliform. Xiandai Shipin Keji.

[25] Carriere D., Meunier S.J., Tropper F.D., Cao S., Roy R. (2000). Phase transfer catalysis toward the synthesis of *O*-, *S*-, *Se*- and *C*-glycosides. J. Mol. Catal. A Chem..

[26] Walsh J.S., Patanella J.E., Halm K.A., Facchine K.L. (1995). An improved HPLC assay for the assessment of liver slice metabolic viability using 7-ethoxycoumarin. Drug Metab. Dispos..

[27] Courtin-Duchateau M.C., Veyrières A. (1978). Synthesis of 4-methylumbelliferyl 1,2-*cis*-glycosides. Carbohydr. Res..

[28] Vervoort A., de Bruyne C.K. (1970). Synthesis of substituted phenyl α-d-mannopyranosides. Carbohydr. Res..

[29] Woollen J.W., Walker P.G. (1965). The fluorimetric estimation of β-glucuronidase in blood plasma. Clin. Chim. Acta.

[30] Szweda R., Spohr U., Lemieux R.U., Schindler D., Bishop D.F., Desnick R.J. (1989). Synthesis of 4-methylumbelliferyl glycosides for the detection of α- and β-d-galactopyranosaminidases. Can. J. Chem..

[31] Chilvers K.F., Perry J.D., James A.L., Reed R.H. (2001). Synthesis and evaluation of novel fluorogenic substrates for the detection of bacterial beta-galactosidase. J. Appl. Microbiol..

[32] Marsh C.A., Levvy G.A. (1956). Synthesis of 4-methylumbelliferone β-d-glucuronide, a substrate for the fluorimetric assay of β-glucuronidase. Nature.

[33] Lopez-Lopez M.A., Balbuzano-Deus A., Rodriguez-Dominguez J.C., Mesa Hernandez M., Fernandez-Villalobo A., Reyes Y.I., Kirsch G. (2007). A new synthetic route to 4-methylumbelliferyl-β-d-glucopyranosiduronic acid (MUG). Synlett.

[34] Brown R.T., Stachulski A.V. (1997). Intermediates for glucuronide synthesis: 7-Hydroxycoumarin glucuronide. J. Chem. Res. Synop..

[35] Pearson A.G., Kiefel M.J., Ferro V., von Itzstein M. (2005). Towards the synthesis of aryl glucuronides as potential heparanase probes. An interesting outcome in the glycosidation of glucuronic acid with 4-hydroxycinnamic acid. Carbohydr. Res..

[36] Stachulski A.V., Jenkins G.V. (1998). The synthesis of *O*-glucuronides. Nat. Prod. Rep..

[37] Lee Y.S., Rho E.S., Min Y.K., Kim B.T., Kim K.H. (2001). Practical beta-stereoselective *O*-glycosylation of phenols with penta-*O*-acetyl-beta-d-glucopyranose. J. Carbohydr. Chem..

[38] Zhu X.R., Zheng Y., Wang P., Yang S.B., Chen R., Zhu Y.Q. (2010). Synthesis of two metabolites of edaravone. J. Chin. Pharm. Sci..

[39] Toshima K., Tatsuta K. (1993). Recent progress in *O*-glycosylation methods and its application to natural products synthesis. Chem. Rev..

[40] Andersson M., Oscarson S. (1992). Synthesis of *O*-glycopyranosyl-*N*-hydroxysuccinimides of glucose and lactose and their opening by nucleophiles into prespacer glycosides. Glycoconj. J..

[41] Matsumoto T., Hosoya T., Suzuki K. (1990). Improvement in *O→C*-glycoside rearrangement approach to *C*-aryl glycosides: Use of 1-*O*-acetyl sugar as stable but efficient glycosyl donor. Tetrahedron Lett..

[42] Zeng S., Chen L., Liu D., Li H., Wu G., Liao Y. (2013). Synthesis of p-nitrophenyl α-d-xylopyranoside. Huaxue Shiji.

[43] De Bruyne C.K., Loontiens F.G. (1965). A fluorigenic substrate for β-d-xylosidase. Naturwissenschaften.

[44] Schramm V.L., Furneaux R.H., Tyler P.C., Clinch K. (1997). Enzyme Detection/Assay Method and Substrates. Patent Cooperation Treaty International Publication.

[45] Yamaguchi M., Horiguchi A., Fukuda A., Minami T. (1990). Novel synthesis of aryl 2,3,4,6-tetra-*O*-acetyl-d-glucopyranosides. J. Chem. Soc. Perkin Trans. 1.

[46] Demchenko A.V. (2003). 1,2-*cis O*-Glycosylation: Methods, strategies, principles. Curr. Org. Chem..

[47] Zhu X., Schmidt R. (2009). New principles for glycoside-bond formation. Angew. Chem. Int. Ed..

[48] Nakahara Y., Ogawa T. (1987). A highly efficient, practical, and stereoselective approach to the synthesis of α 1→4 linked galactooligosaccharides. Tetrahedron Lett..

[49] Demchenko A.V., Rousson E., Boons G.J. (1999). Stereoselective 1,2-*cis*-galactosylation assisted by remote neighboring group participation and solvent effects. Tetrahedron Lett..

[50] Stachulski A.V., Meng X. (2013). Glucuronides from metabolites to medicines: A survey of the *in vivo* generation, chemical synthesis and properties of glucuronides. Nat. Prod. Rep..

[51] Needs P.W., Williamson G. (2001). Syntheses of daidzein-7-yl beta-d-glucopyranosiduronic acid and daidzein-4′,7-yl di-beta-d-glucopyranosiduronic acid. Carbohydr. Res..

[52] Khan M.K., Rakotomanomana N., Loonis M., Dangles O. (2010). Chemical synthesis of citrus flavanone glucuronides. J. Agric. Food Chem..

[53] Walker J.R., Alshafie G., Nieves N., Ahrens J., Clagett-Dame M., Abou-Issa H., Curley R.W. (2006). Synthesis and preliminary chemotherapeutic evaluation of the fully C-linked glucuronide of *N*-(4-hydroxyphenyl)retinamide. Bioorg. Med. Chem..

[54] Lucas R., Alcantara D., Morales J.C. (2009). A concise synthesis of glucuronide metabolites of urolithin-B, resveratrol, and hydroxytyrosol. Carbohydr. Res..

