# Supplementary material for: An Improved Helferich Method for the α/β-Stereoselective Synthesis of 4-Methylumbelliferyl Glycosides for the Detection of Microorganisms
Source: Molecules. 2015 Dec 4;20(12):21681–99. doi: 10.3390/molecules201219789 (PMC6331929; doi:10.3390/molecules201219789)

# Generic Display Report

**Analysis Info**

| | | | |
|---|---|---|---|
| Analysis Name | D:\Data\201505\150519-02\150519-02-5_P1-A-5_01_5069.d | Acquisition Date | 5/19/2015 4:25:49 PM |
| Method | esi_pos_50-1000_with calibration_for 1min.m | Operator | HSJ |
| Sample Name | 150519-02-5 | Instrument | maXis impact |
| Comment | | | |

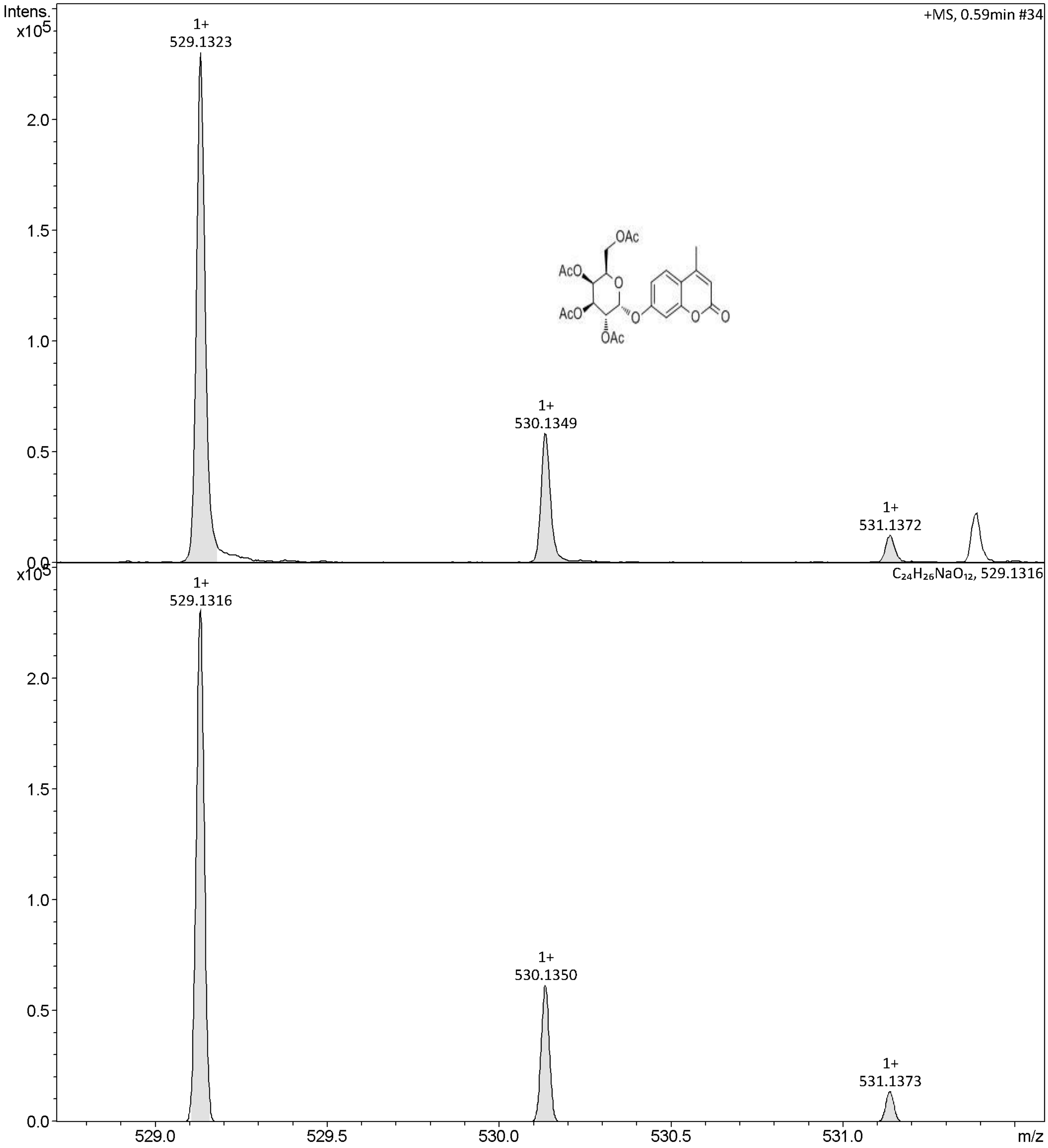

Supplement: Supplementary file 1 [file molecules-20-19789-s001.zip › HRMS data.PDF/HRMS (3c) the protected a┴-D-galactopyranoside.pdf]
