# Supplementary material for: An Improved Helferich Method for the α/β-Stereoselective Synthesis of 4-Methylumbelliferyl Glycosides for the Detection of Microorganisms
Source: Molecules. 2015 Dec 4;20(12):21681–99. doi: 10.3390/molecules201219789 (PMC6331929; doi:10.3390/molecules201219789)

Generic Display Report

Analysis Info

Analysis Name	D:\Data\201505\150519-02\150519-02-11_P1-B-2_01_5075.d	Acquisition Date	5/19/2015 4:35:34 PM
Method	esi_pos_50-1000_with calibration_for 1min.m	Operator	HSJ
Sample Name	150519-02-11	Instrument	maXis impact
Comment			

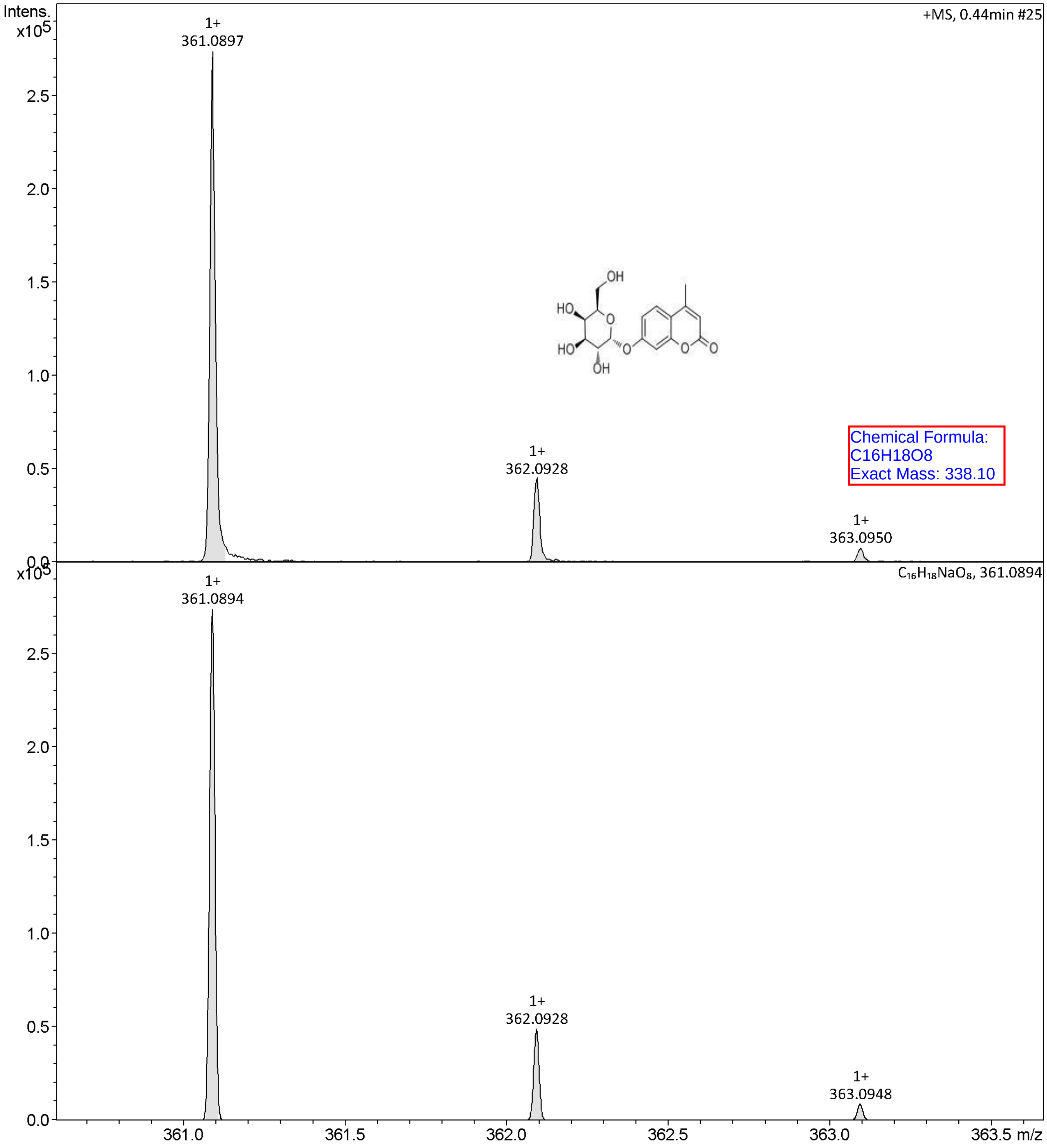

Supplement: Supplementary file 1 [file molecules-20-19789-s001.zip › HRMS data.PDF/HRMS (4c) the a┴-D-galactopyranoside.pdf]
