# Supplementary figures and images for: An Improved Helferich Method for the α/β-Stereoselective Synthesis of 4-Methylumbelliferyl Glycosides for the Detection of Microorganisms
Source: Molecules. 2015 Dec 4;20(12):21681–99. doi: 10.3390/molecules201219789 (PMC6331929; doi:10.3390/molecules201219789)

—7.281

5.797  
5.771

5.332

5.302

5.292

5.290

5.260

5.230

5.191

5.165

5.163

5.135

4.213

—3.765

2.138

2.060

2.060

2.051

—1.613

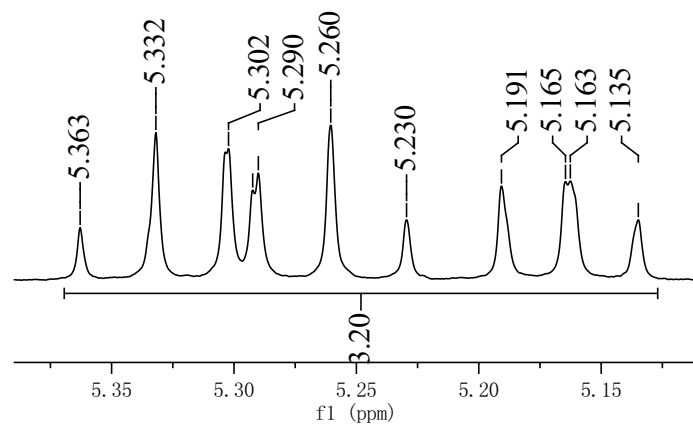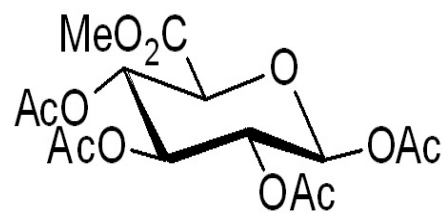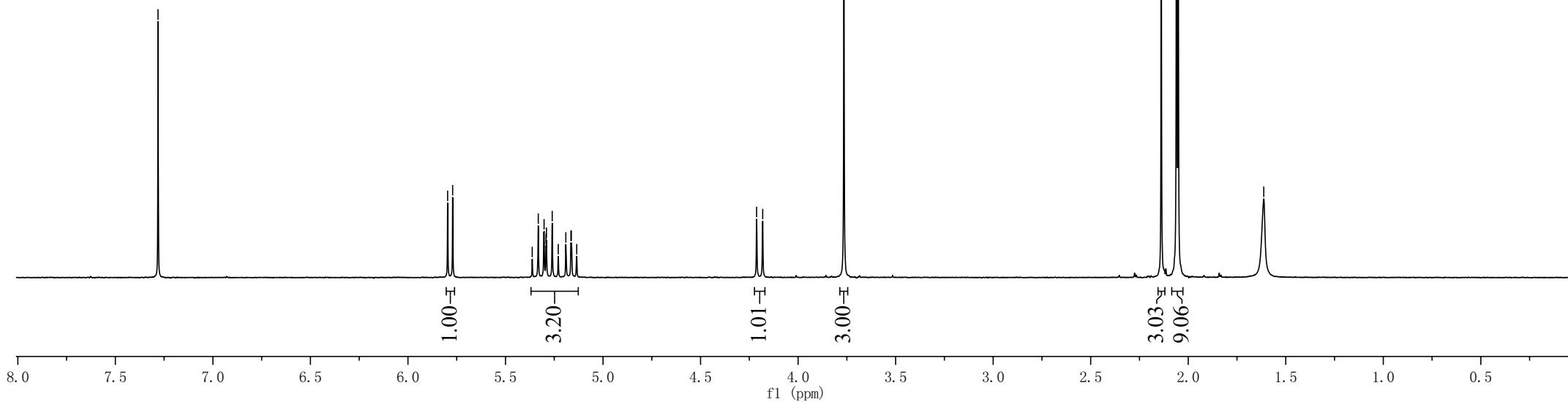

169.88  
169.40  
169.16  
168.81  
166.79

91.31

77.49

77.27

77.07

76.64

72.93

71.77

70.11

68.88

53.00

20.75

20.54

20.51

20.44

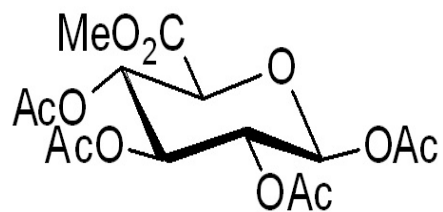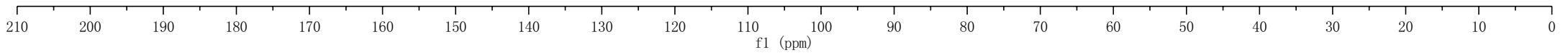

Supplement: Supplementary file 1 [file molecules-20-19789-s001.zip › NMR data.PDF/NMR (2a) Methyl tetra-O-acetyl-a┬-D-glucopyranuronate.pdf]

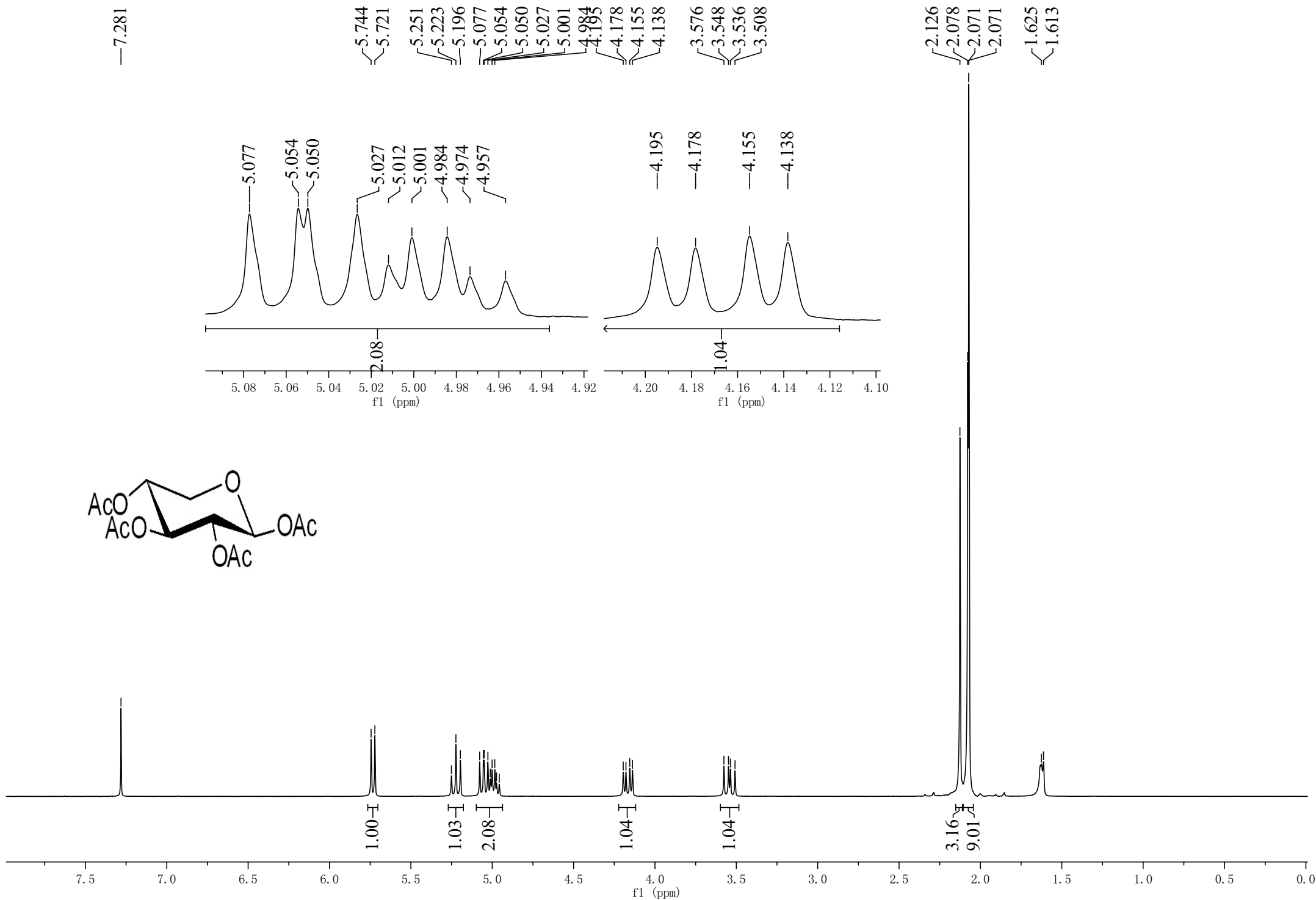

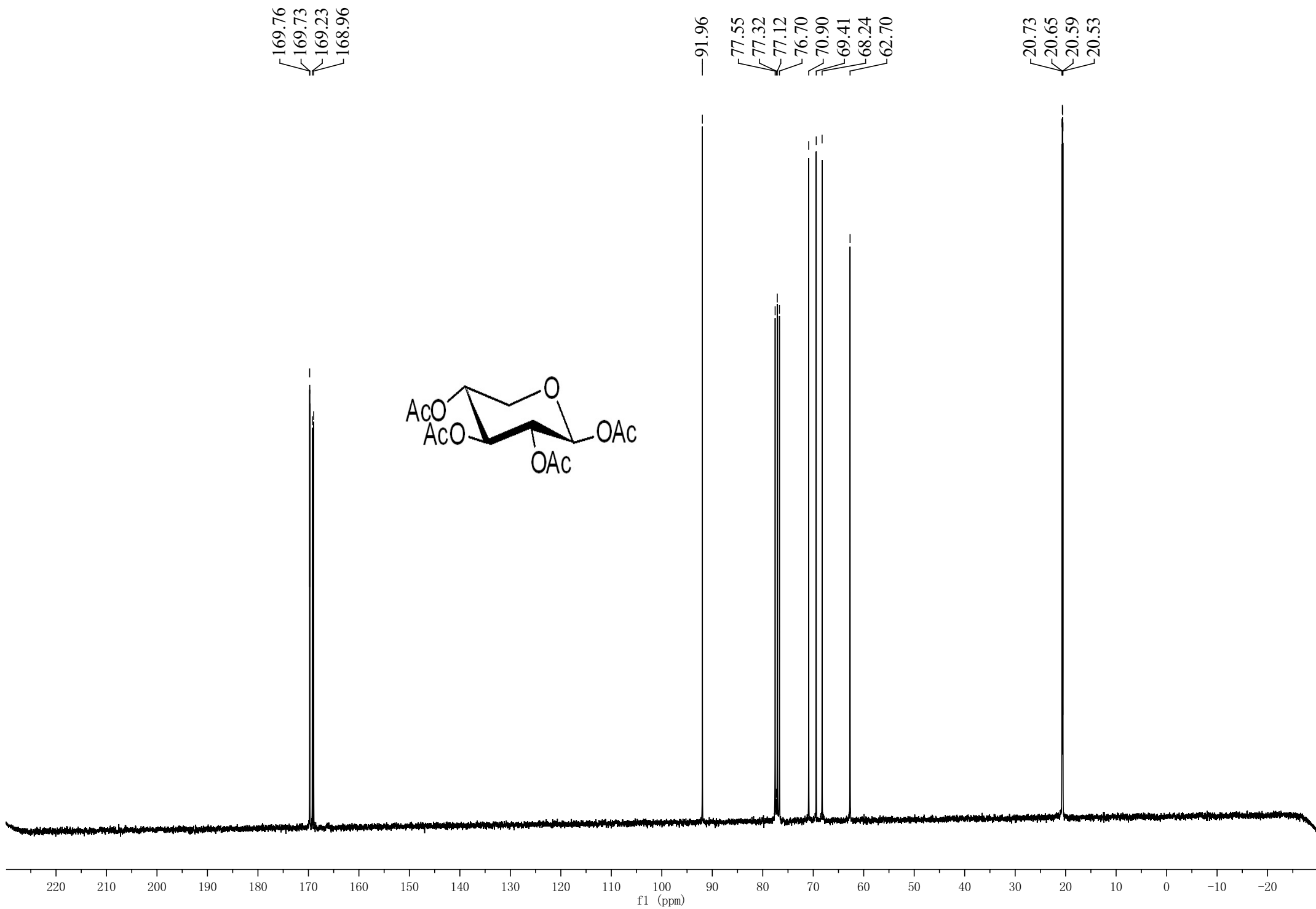

Supplement: Supplementary file 1 [file molecules-20-19789-s001.zip › NMR data.PDF/NMR (2e) a┬-D-xylopyranose tetraacetate.pdf]

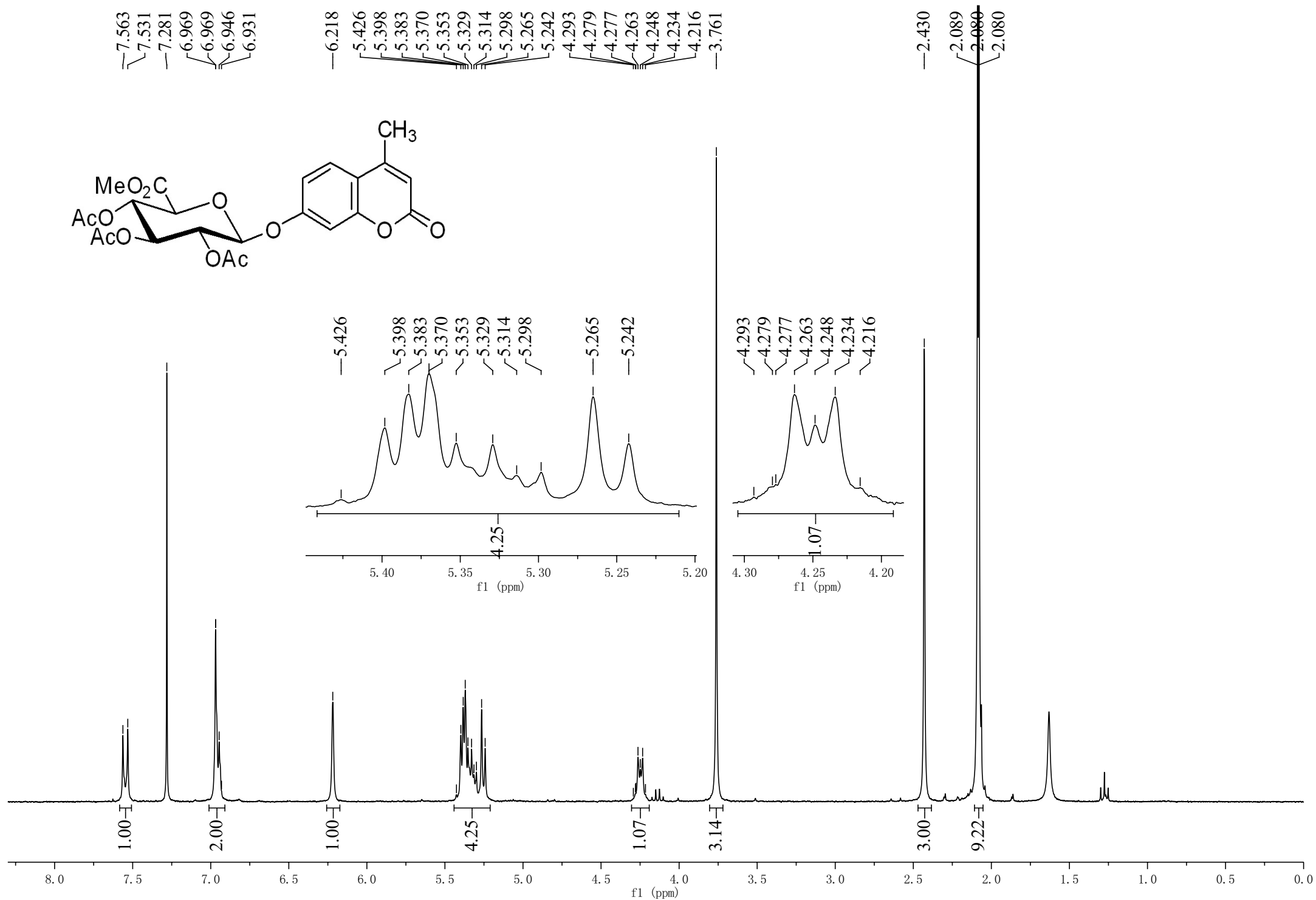

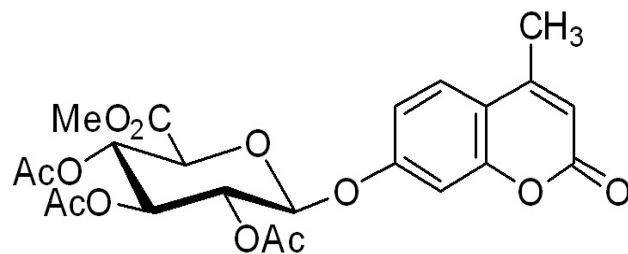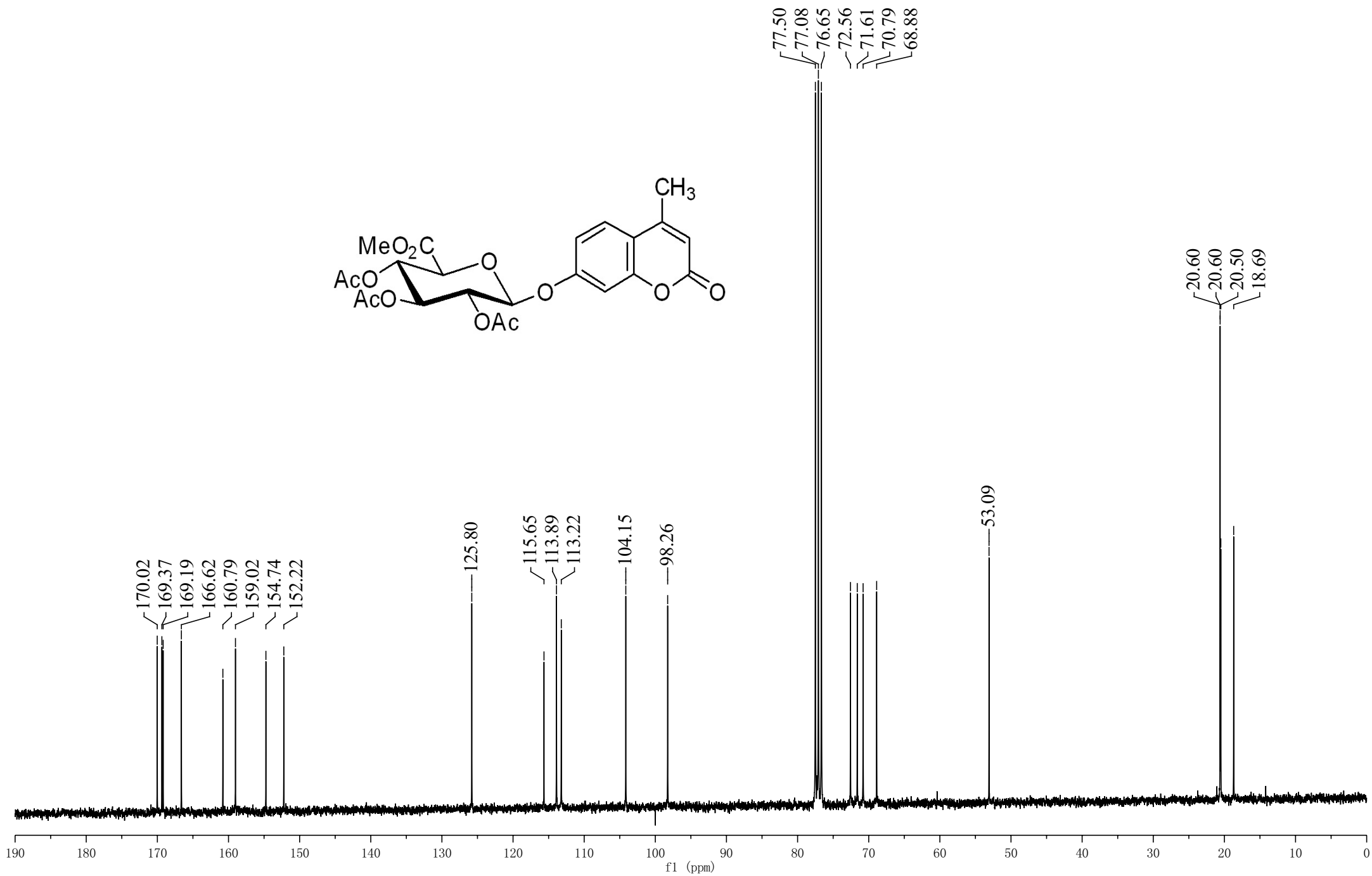

Supplement: Supplementary file 1 [file molecules-20-19789-s001.zip › NMR data.PDF/NMR (3a) the protected a┬-D-glucopyranuronide.pdf]

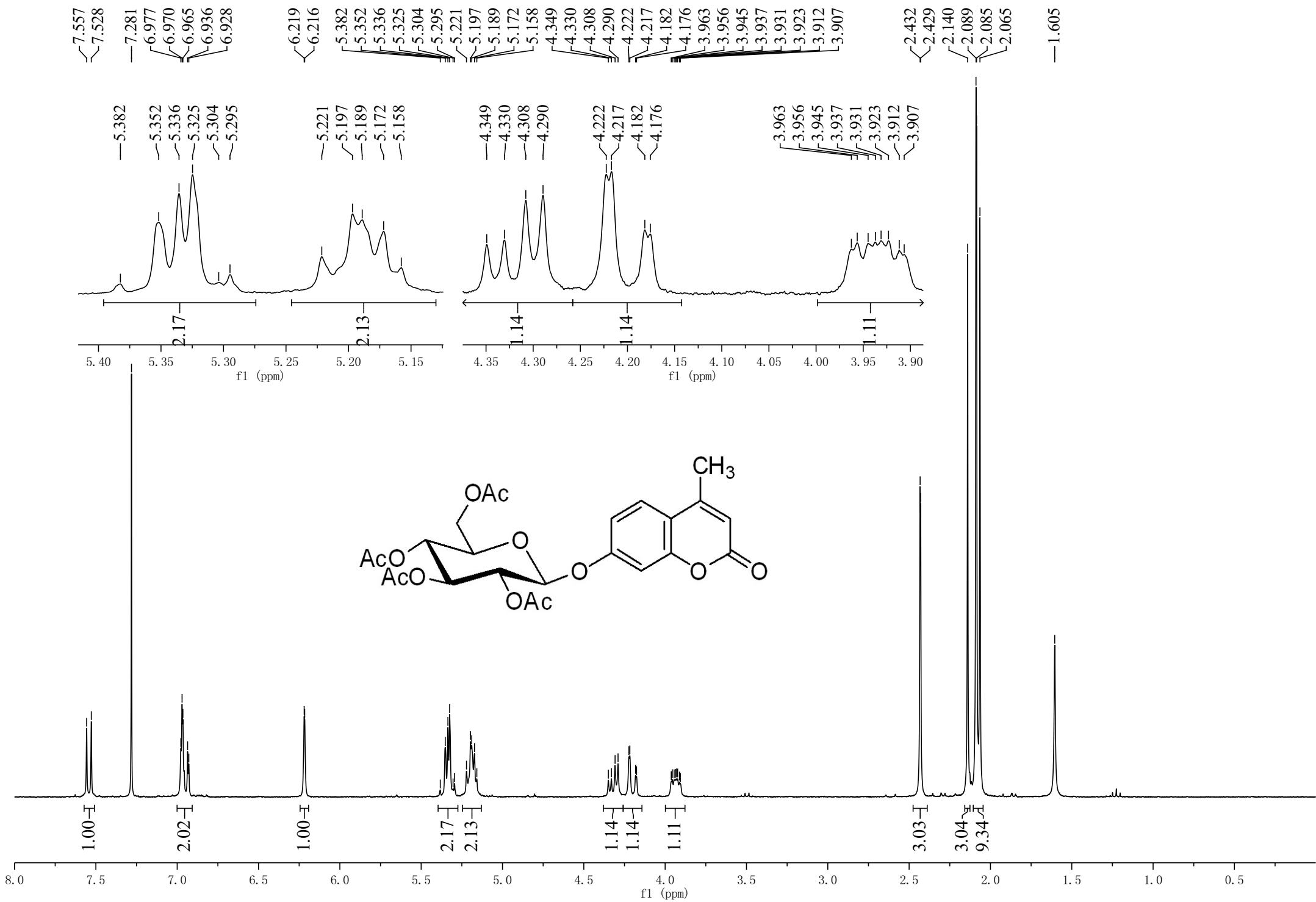

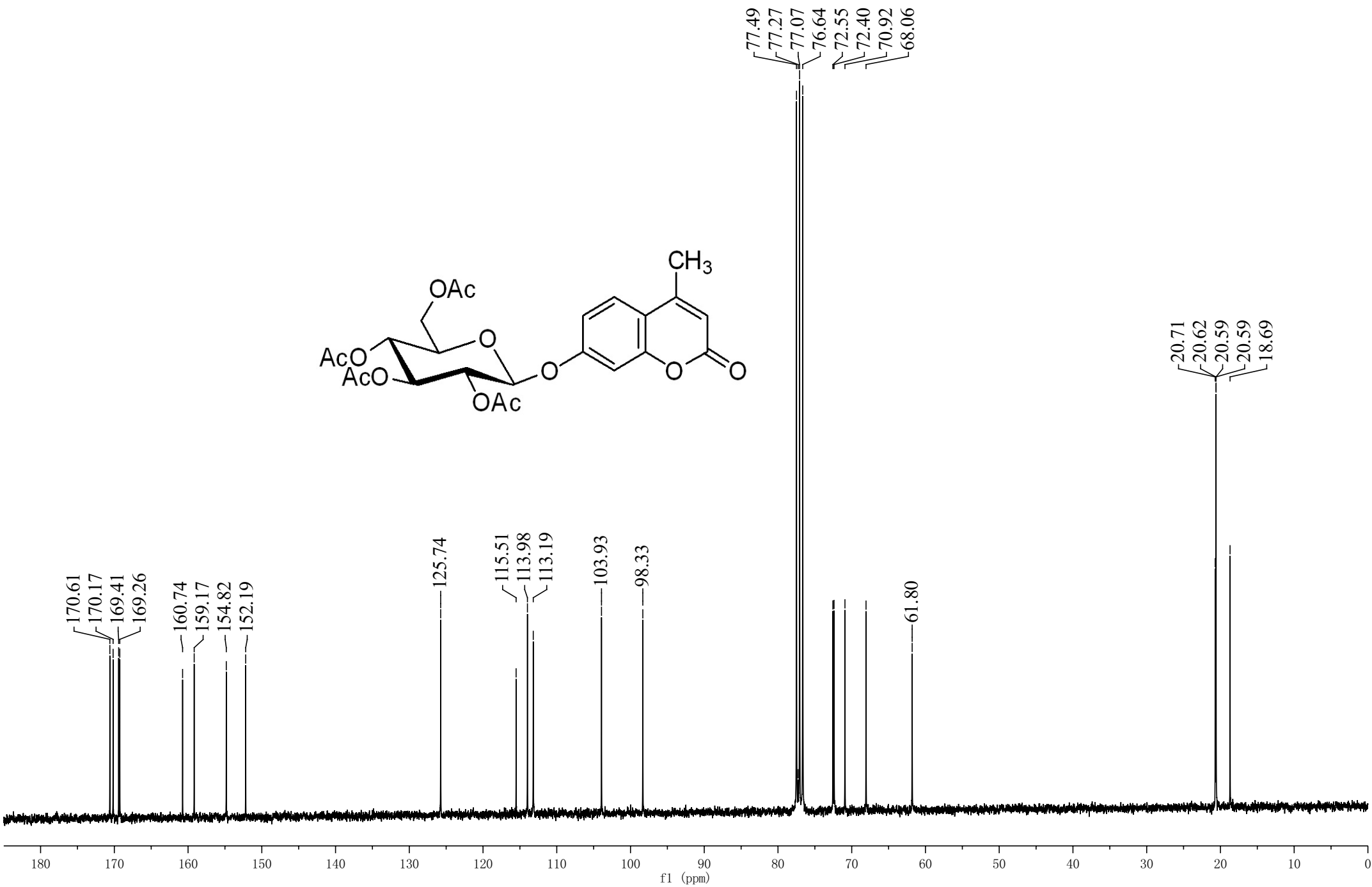

Supplement: Supplementary file 1 [file molecules-20-19789-s001.zip › NMR data.PDF/NMR (3b1) the protected a┬-D-glucopyranoside.pdf]

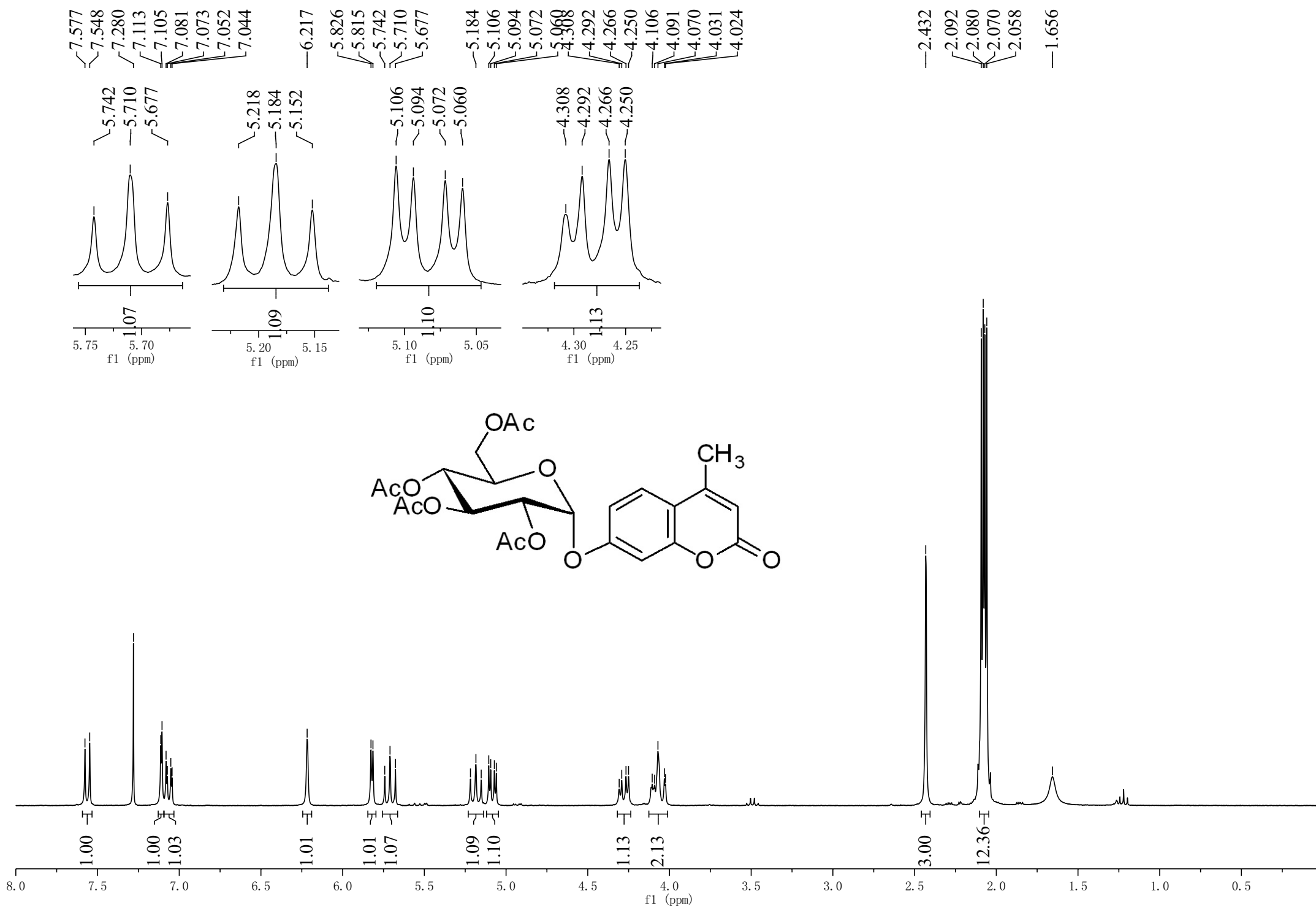

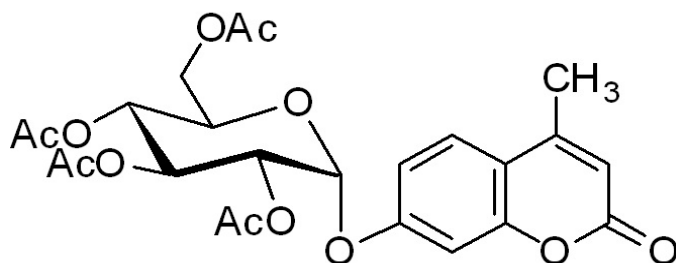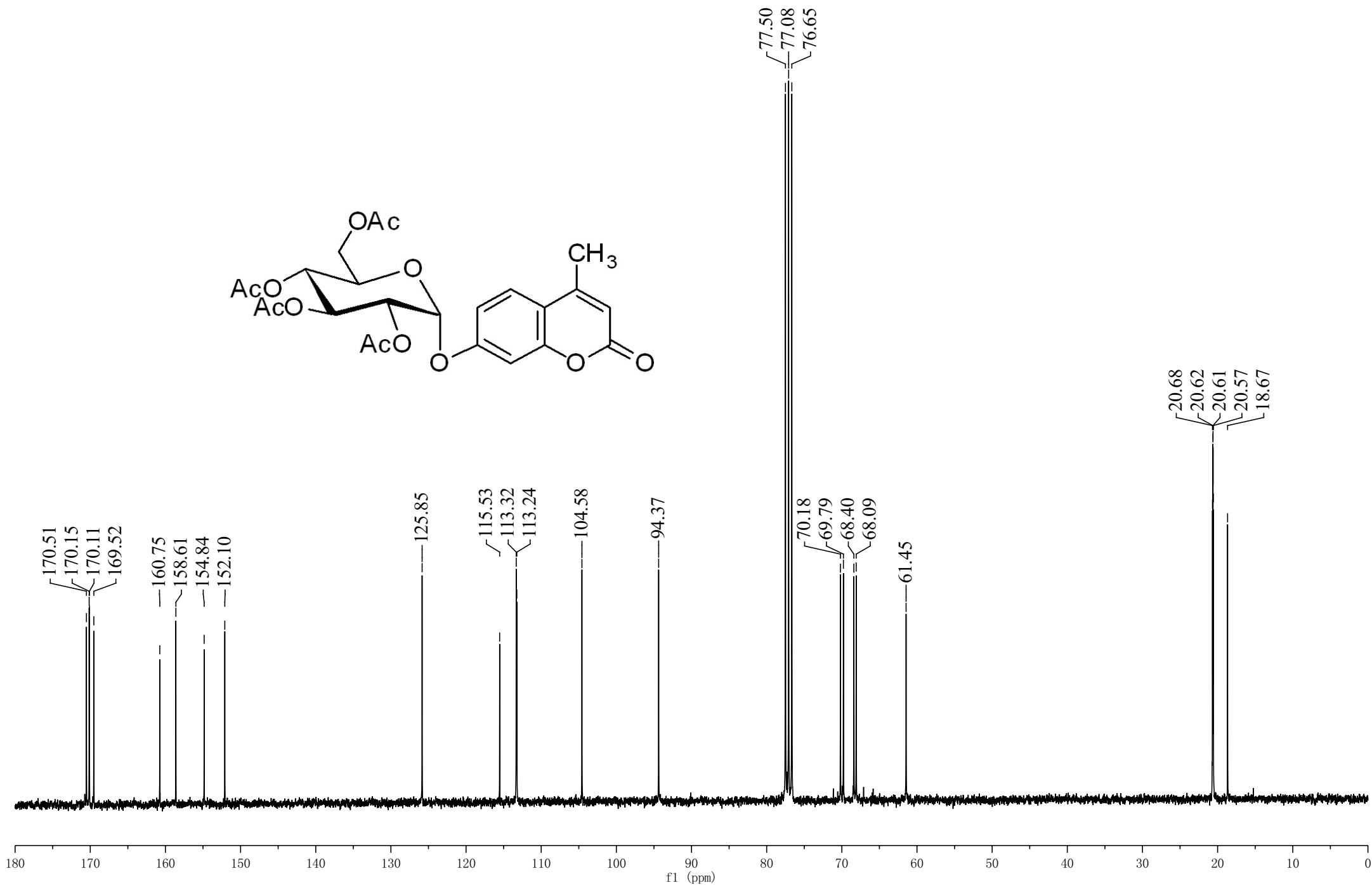

Supplement: Supplementary file 1 [file molecules-20-19789-s001.zip › NMR data.PDF/NMR (3b2) the protected a┴-D-glucopyranoside.pdf]

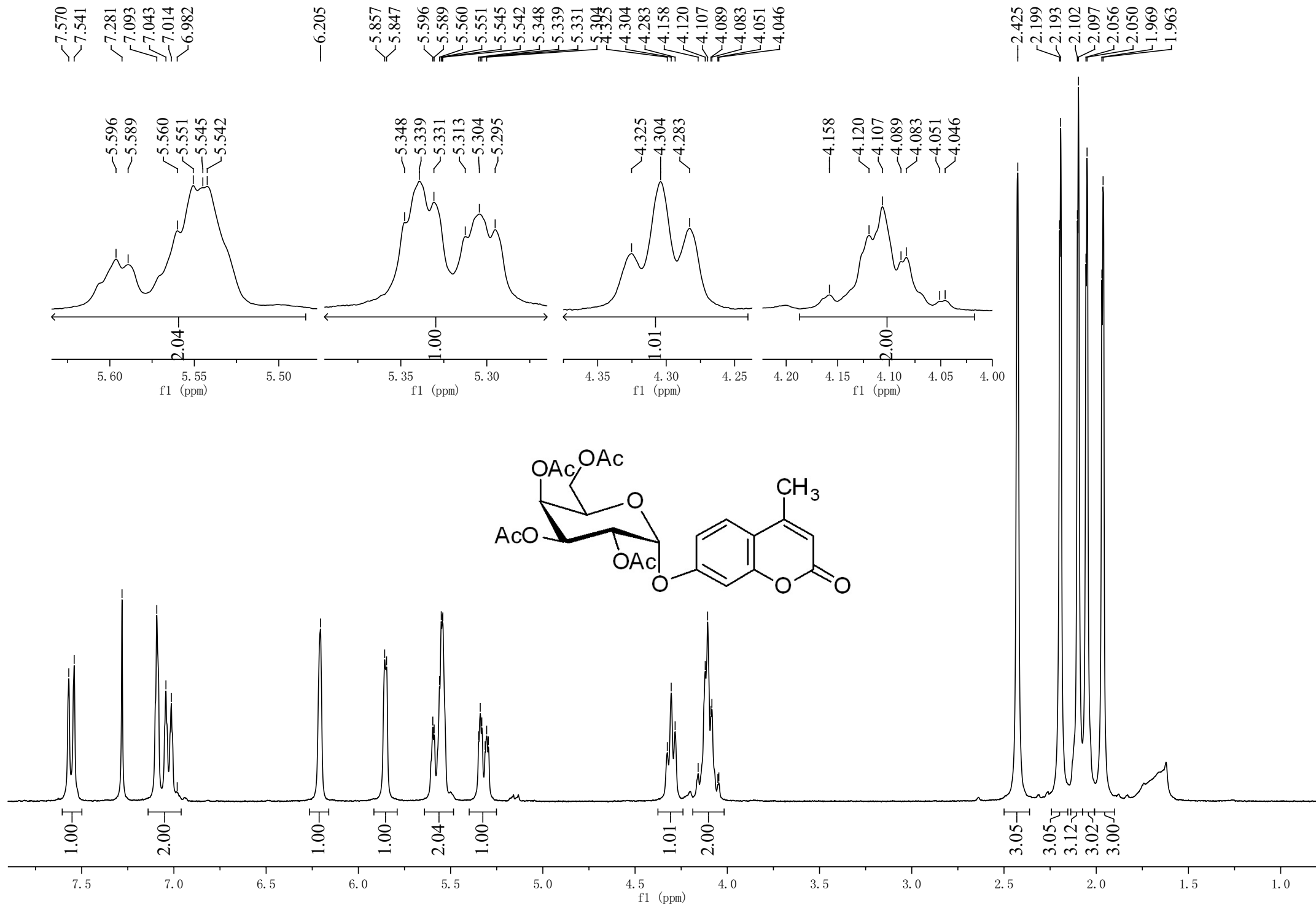

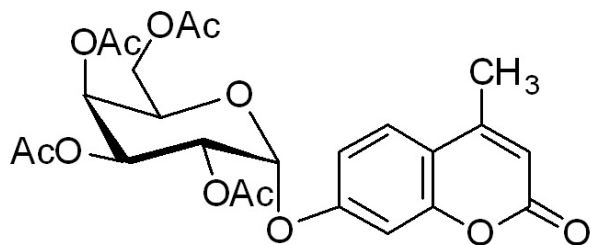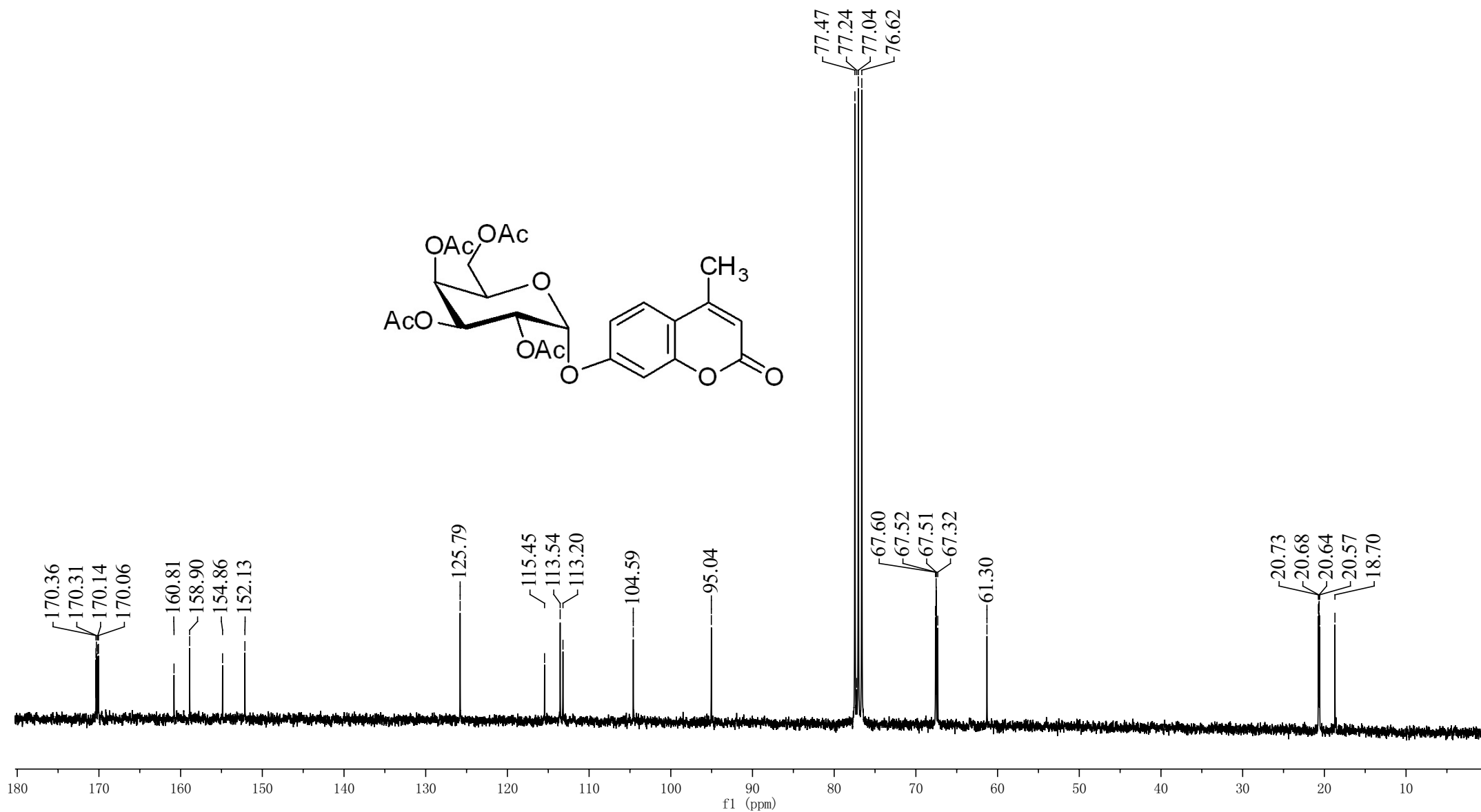

Supplement: Supplementary file 1 [file molecules-20-19789-s001.zip › NMR data.PDF/NMR (3c) the protected a┴-D-galactopyranoside.pdf]

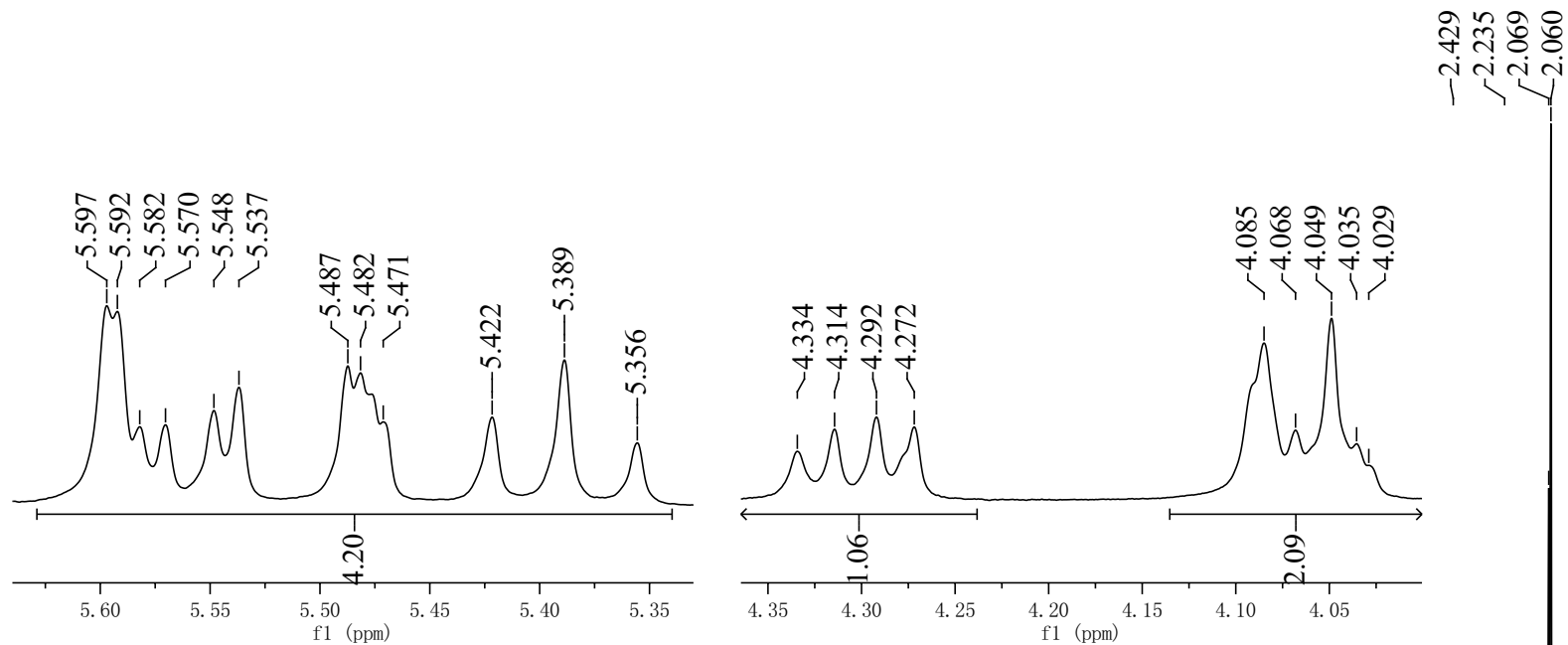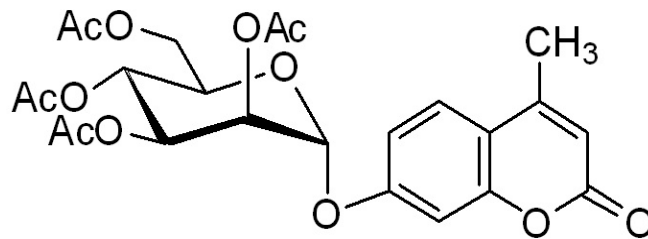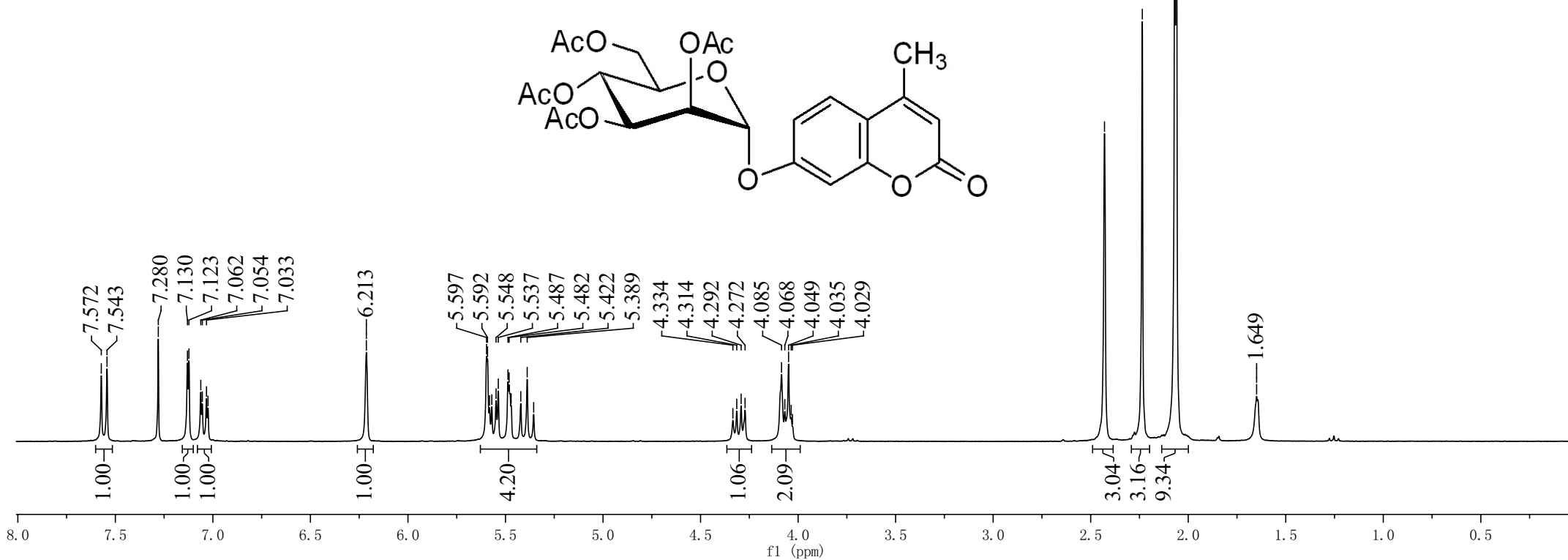

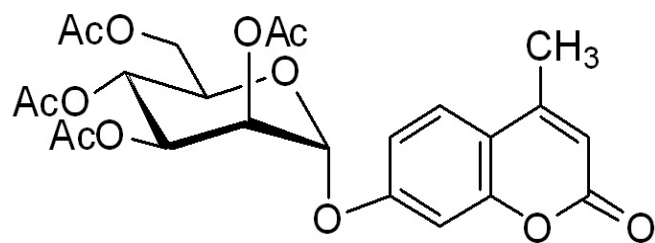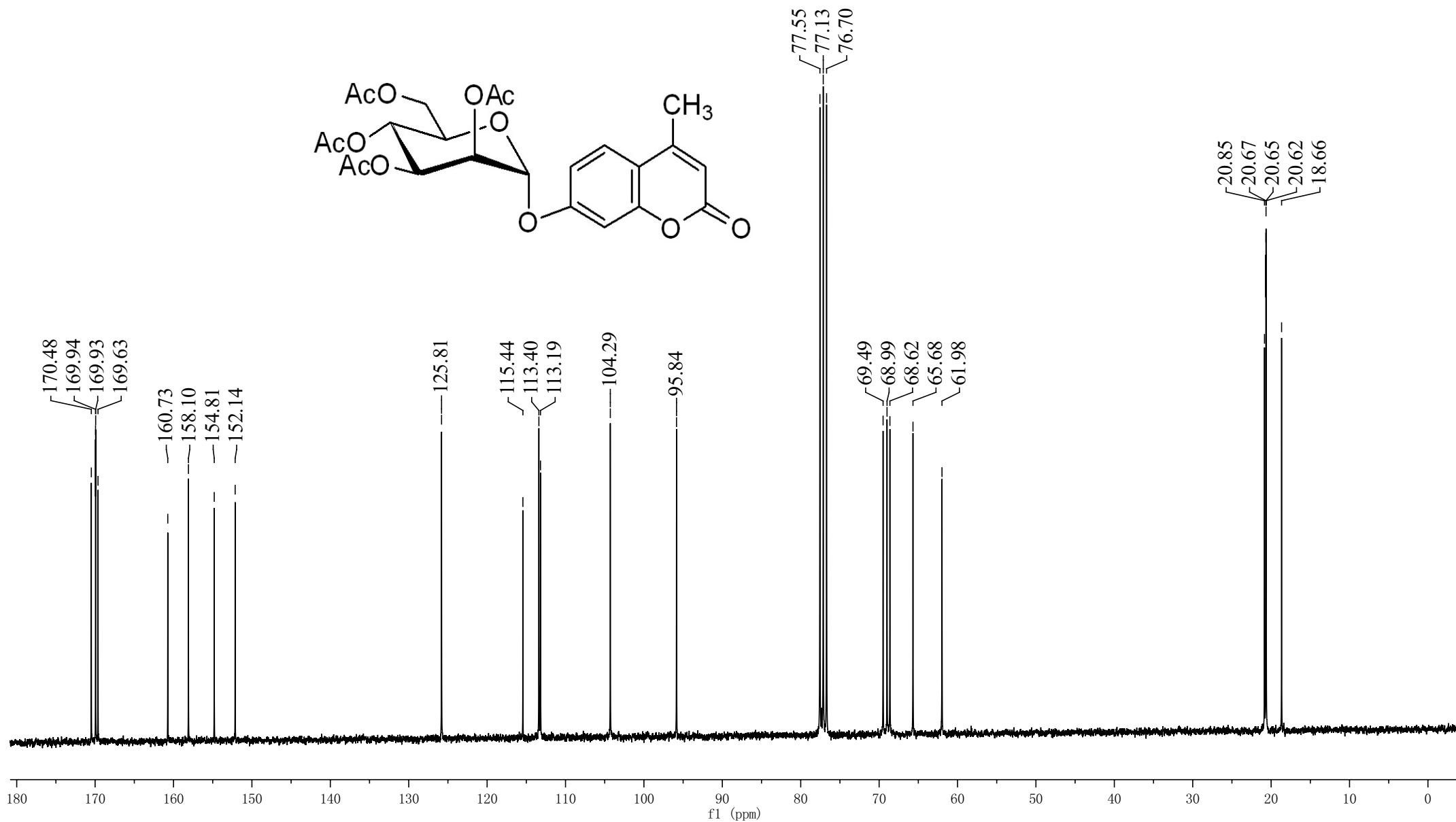

Supplement: Supplementary file 1 [file molecules-20-19789-s001.zip › NMR data.PDF/NMR (3d) the protected a┴-D-mannopyranoside.pdf]

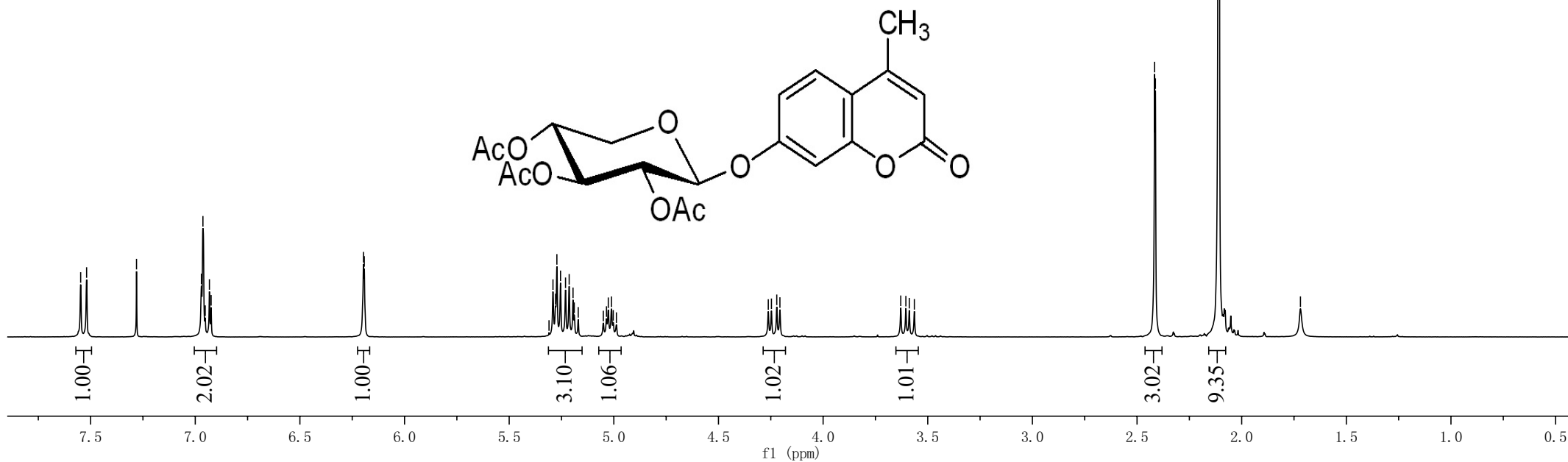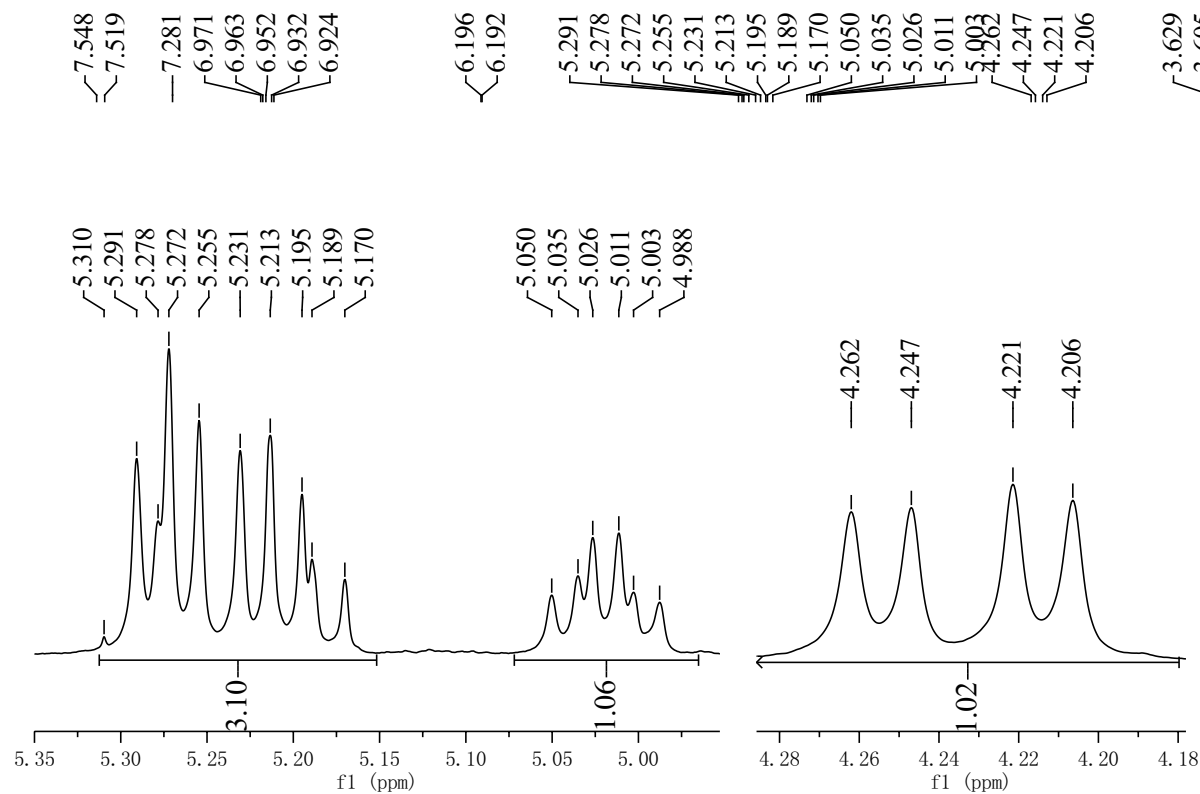

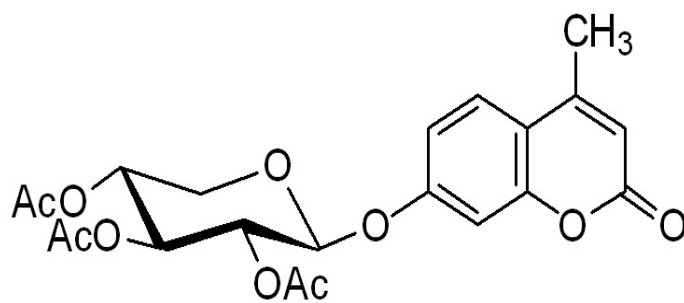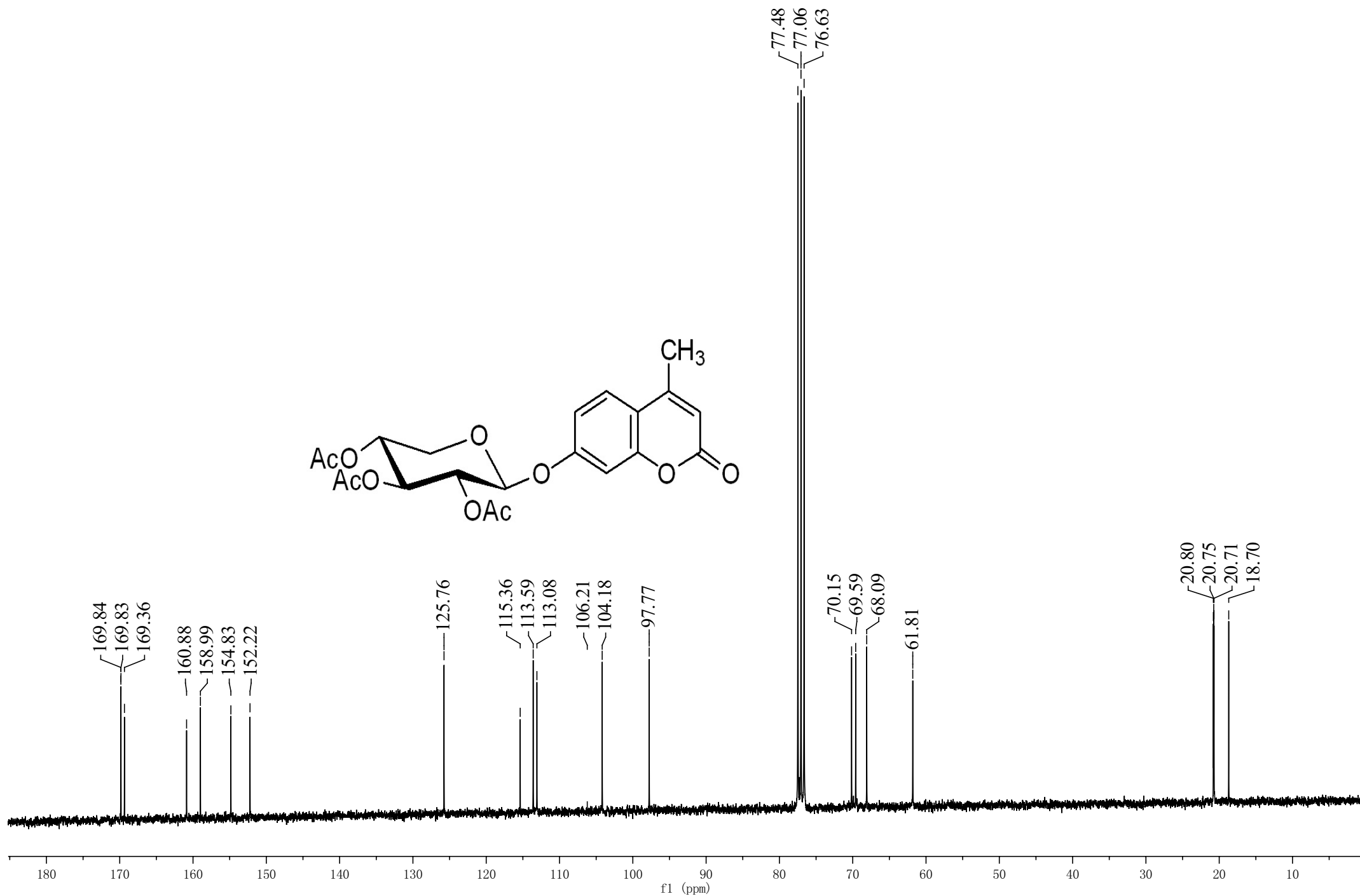

Supplement: Supplementary file 1 [file molecules-20-19789-s001.zip › NMR data.PDF/NMR (3e) the protected a┬-D-xylopyranoside.pdf]

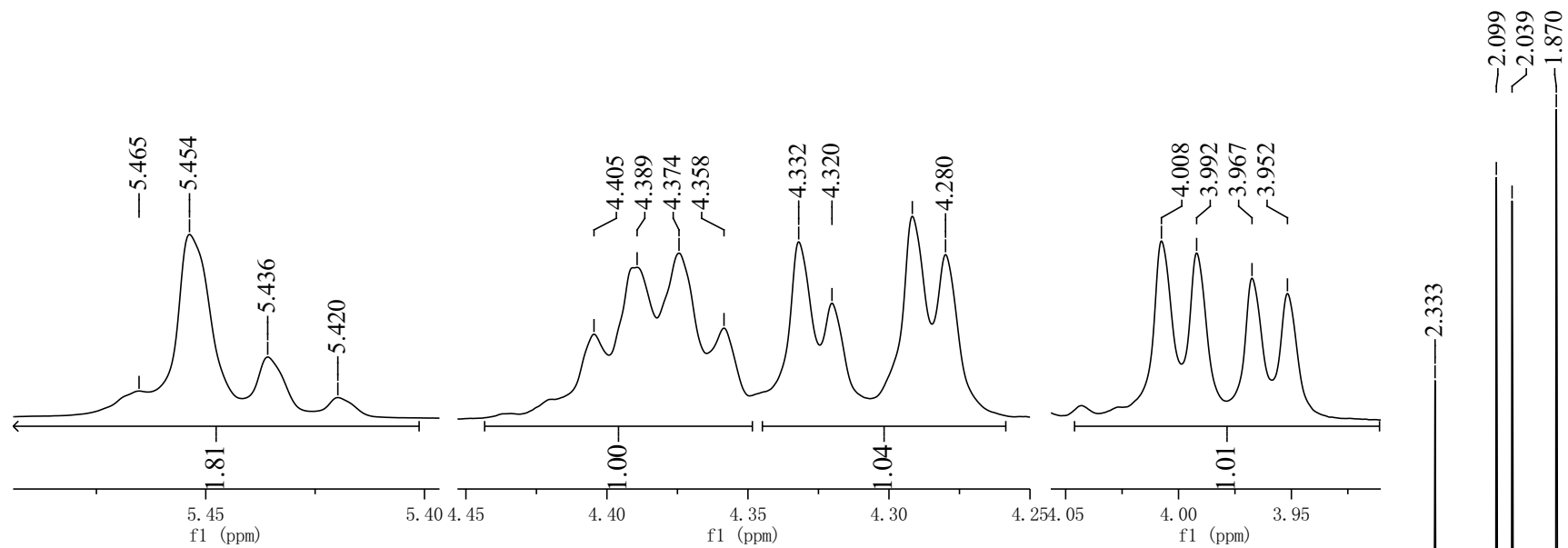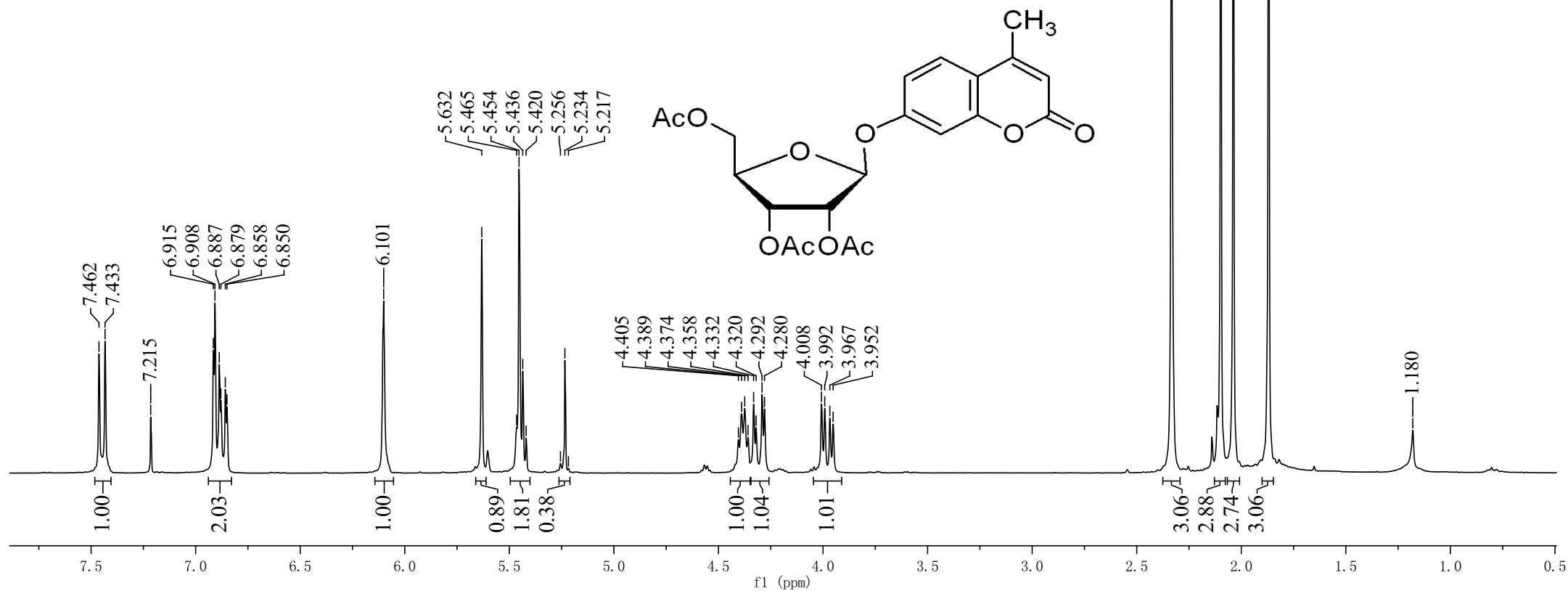

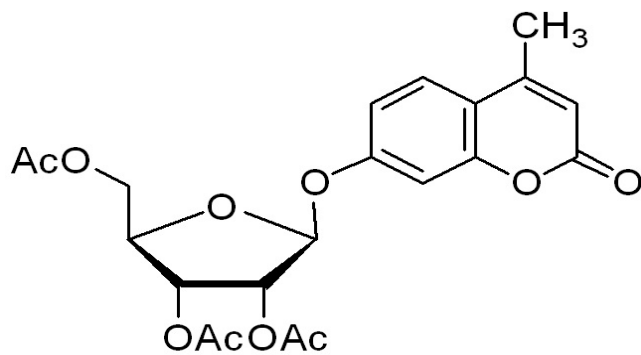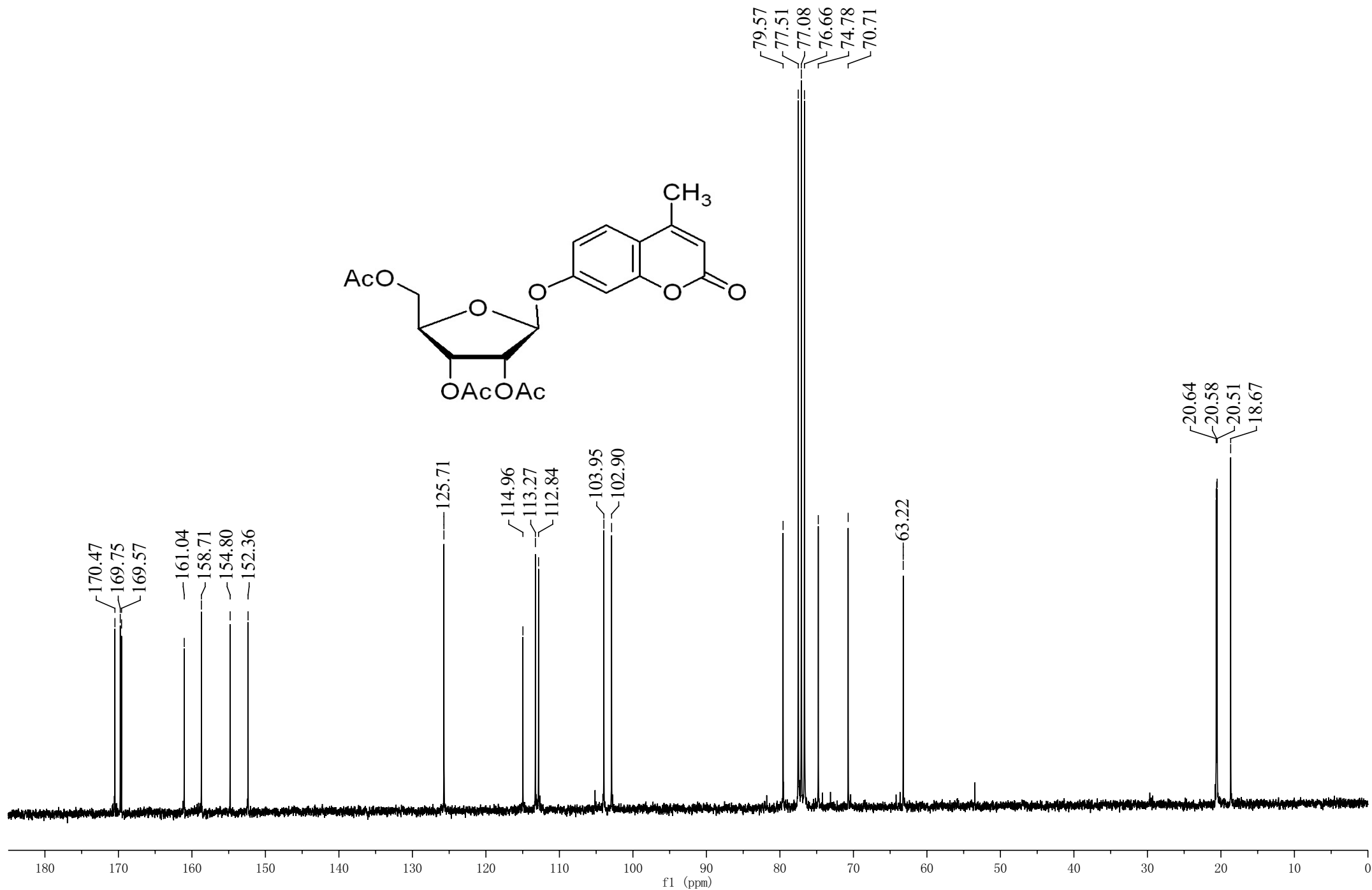

Supplement: Supplementary file 1 [file molecules-20-19789-s001.zip › NMR data.PDF/NMR (3f) the protected a┬-D-ribofuranoside.pdf]

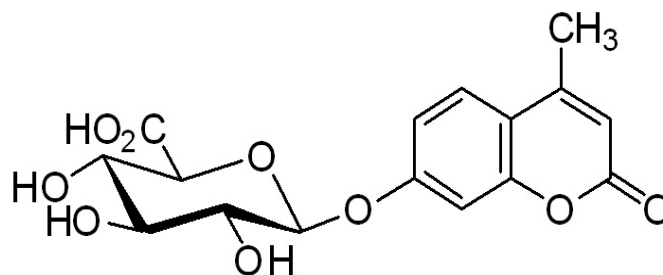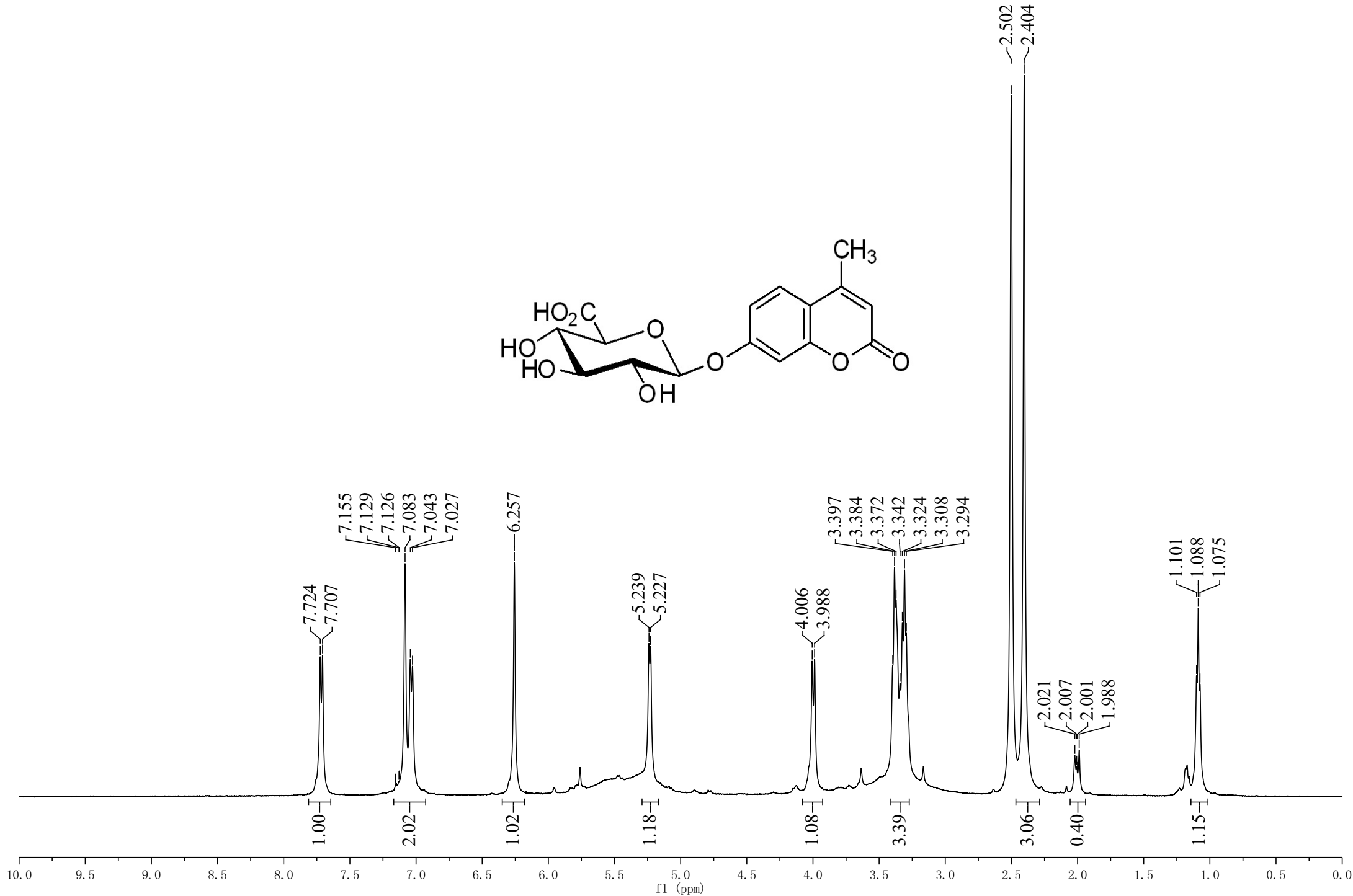

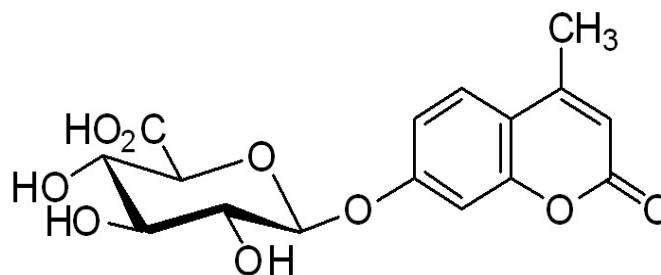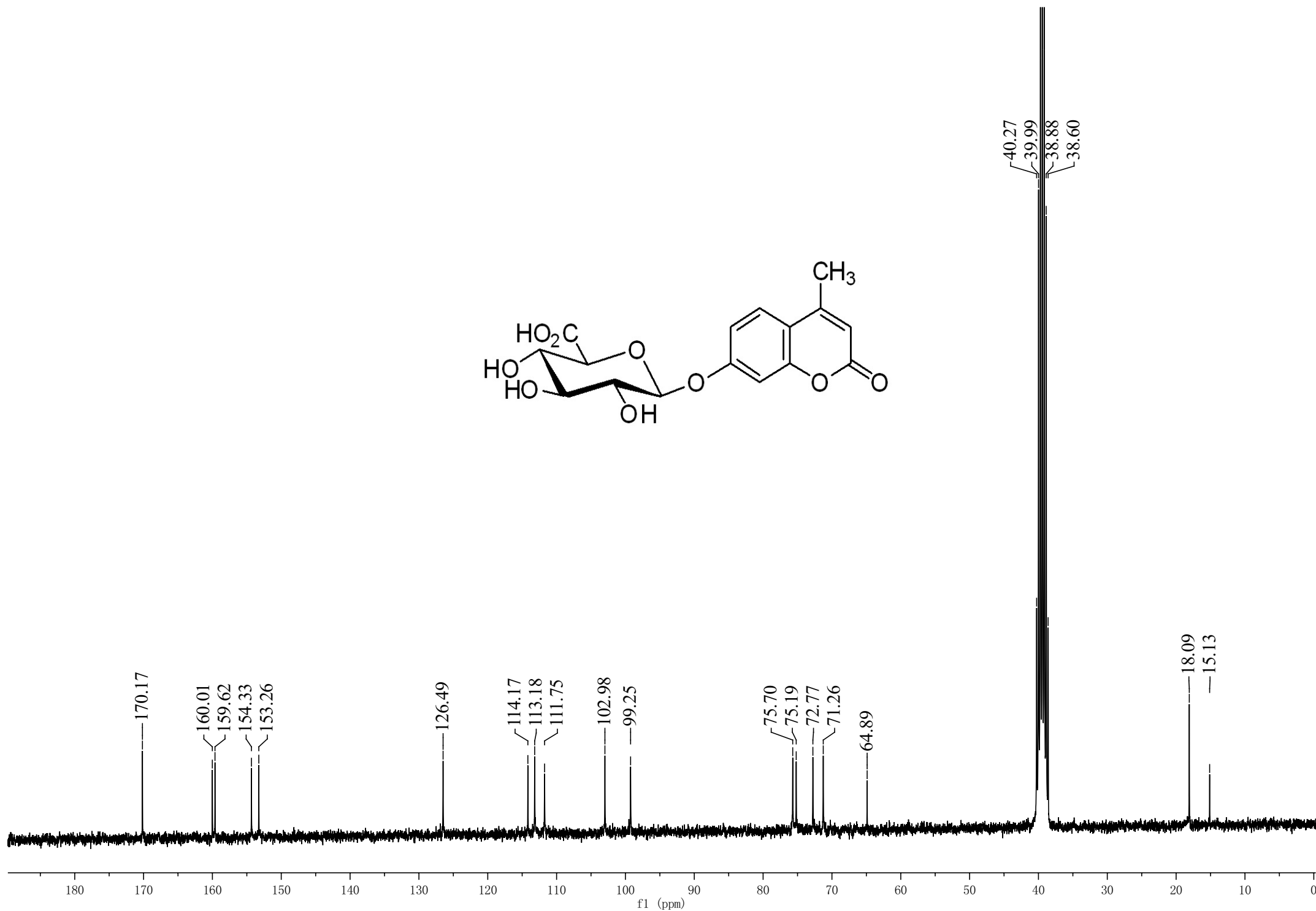

Supplement: Supplementary file 1 [file molecules-20-19789-s001.zip › NMR data.PDF/NMR (4a) the a┬-D-glucopyranosiduronic acid.pdf]

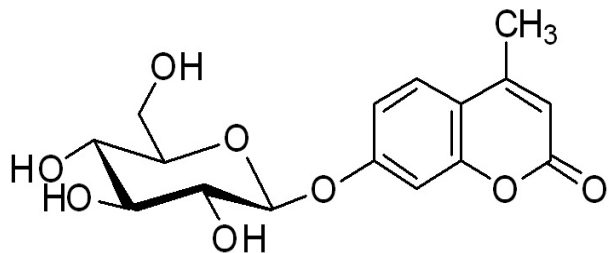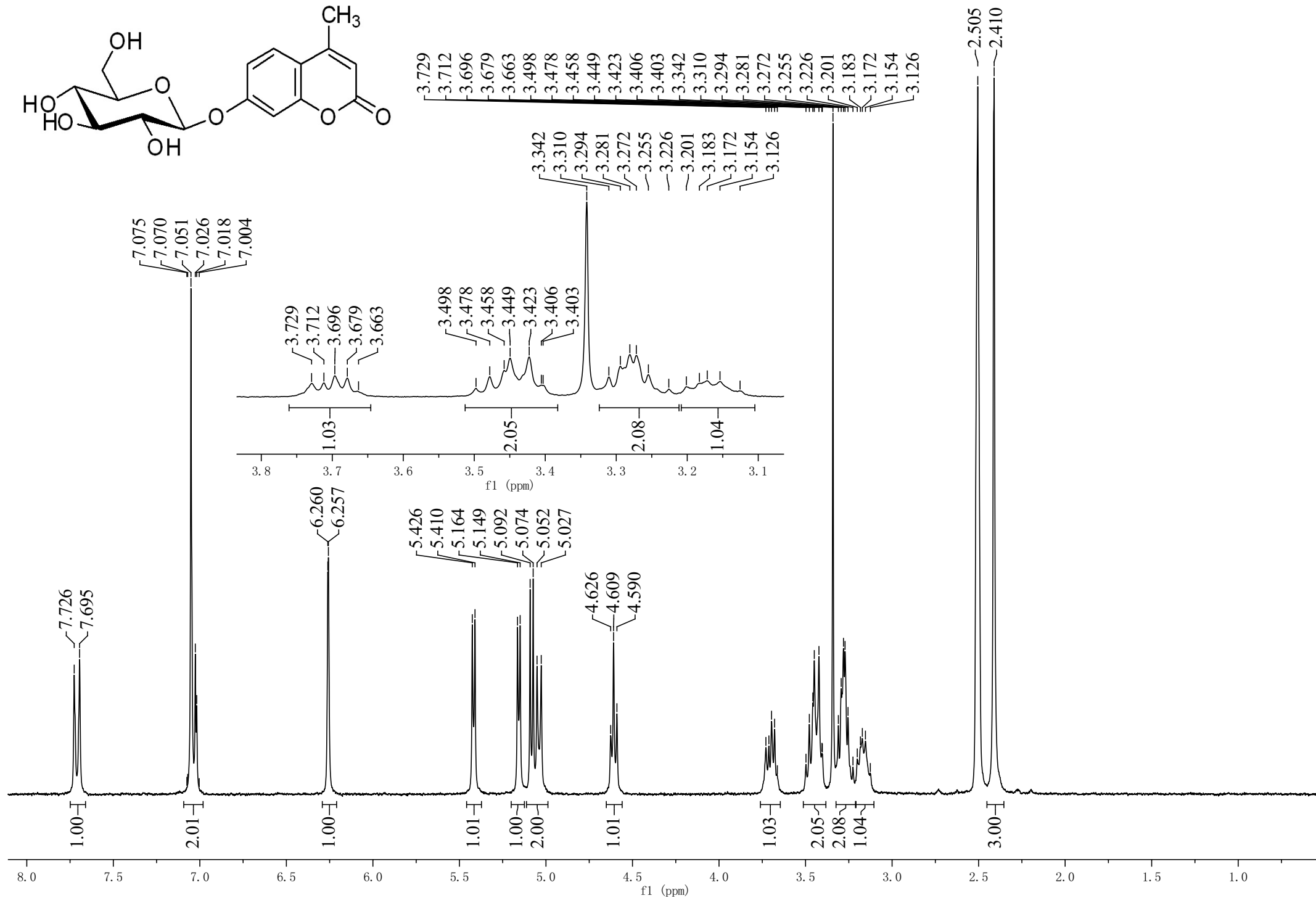

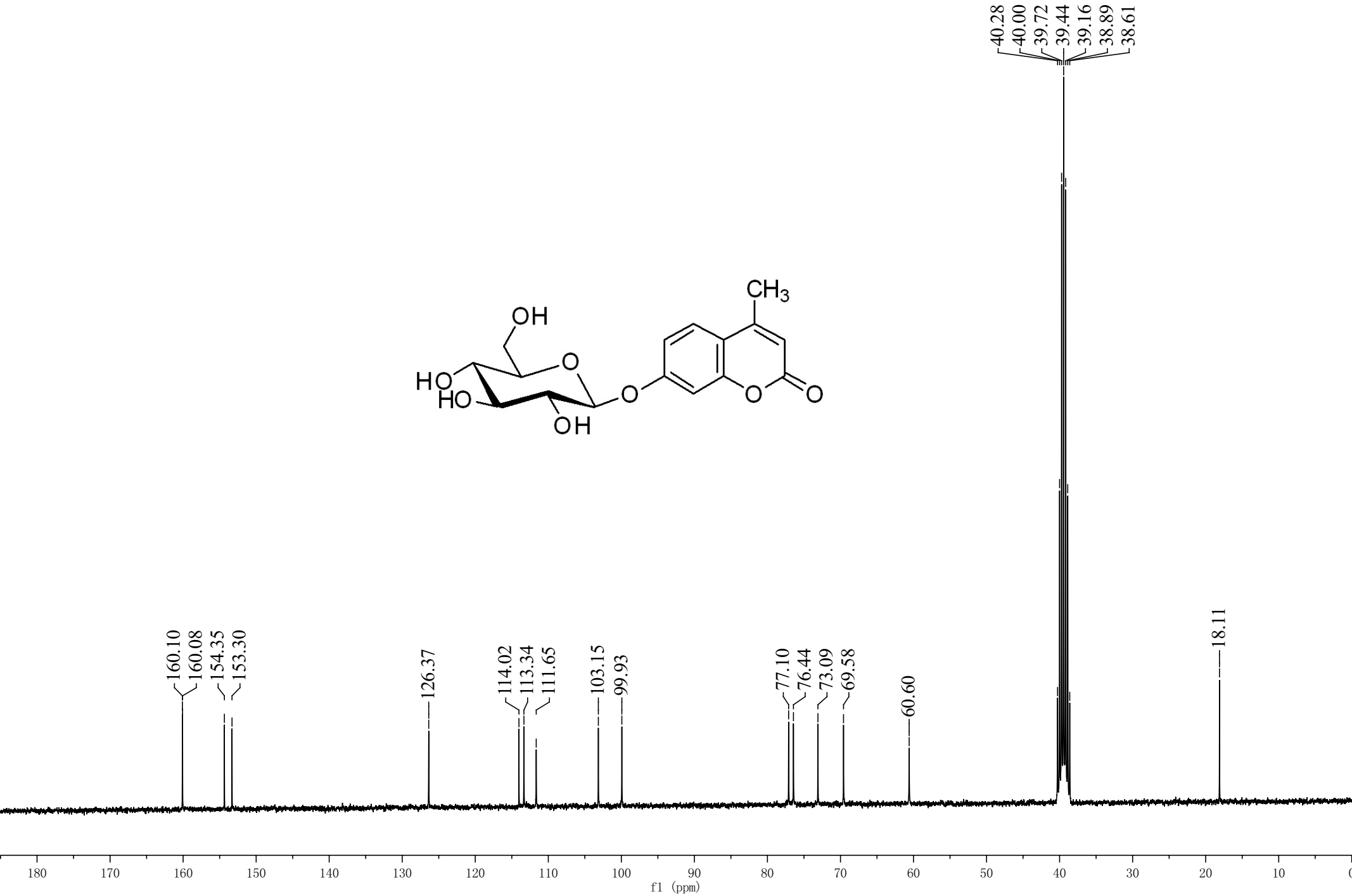

Supplement: Supplementary file 1 [file molecules-20-19789-s001.zip › NMR data.PDF/NMR (4b1) the a┬-D-glucopyranoside.pdf]

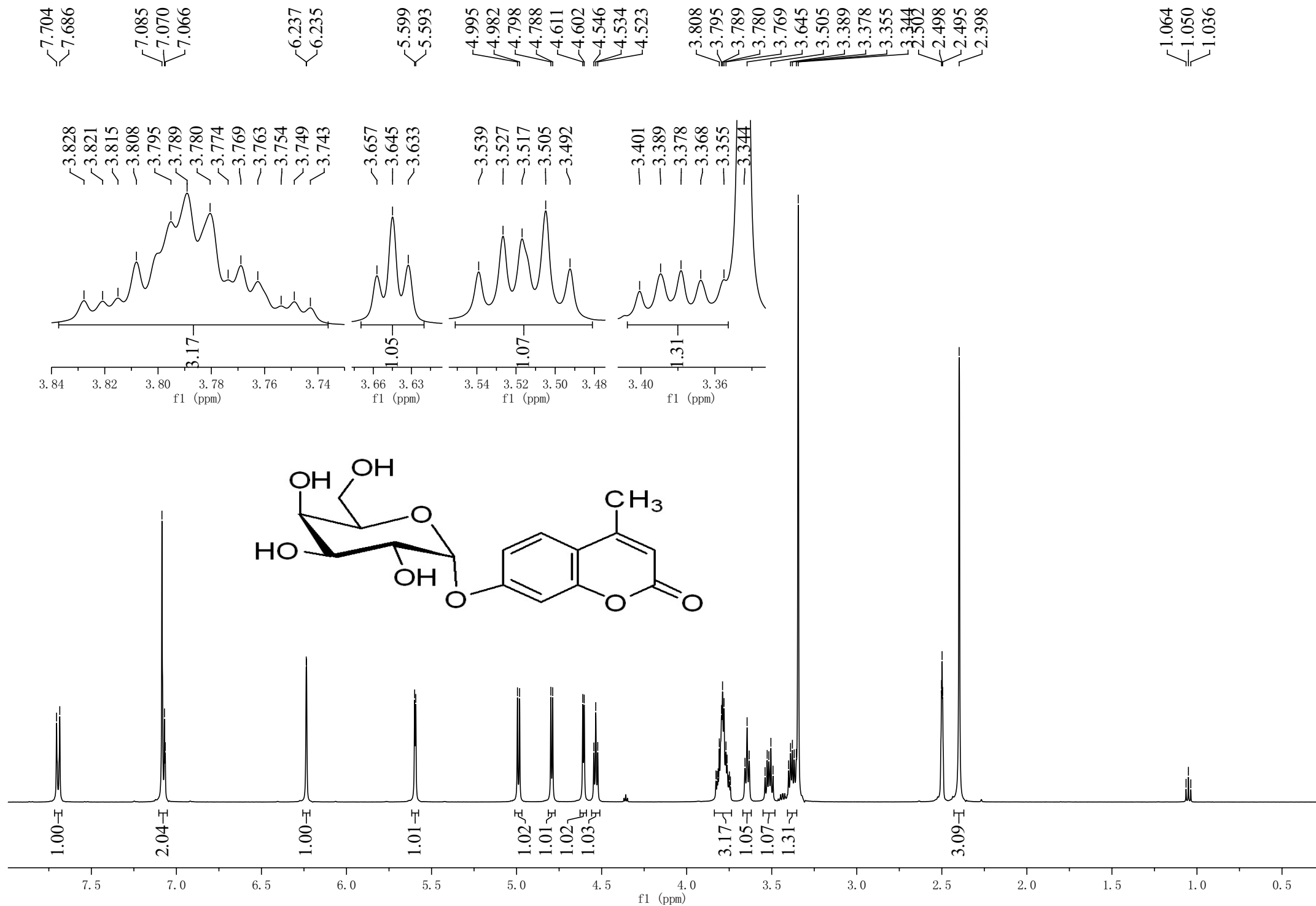

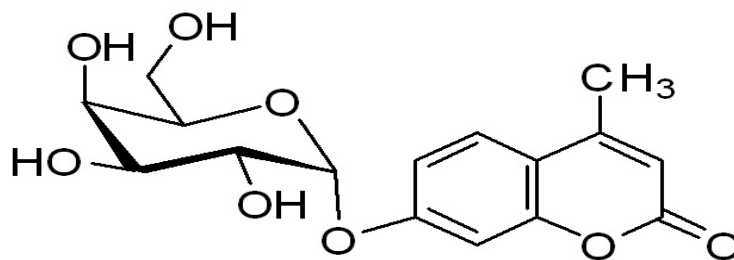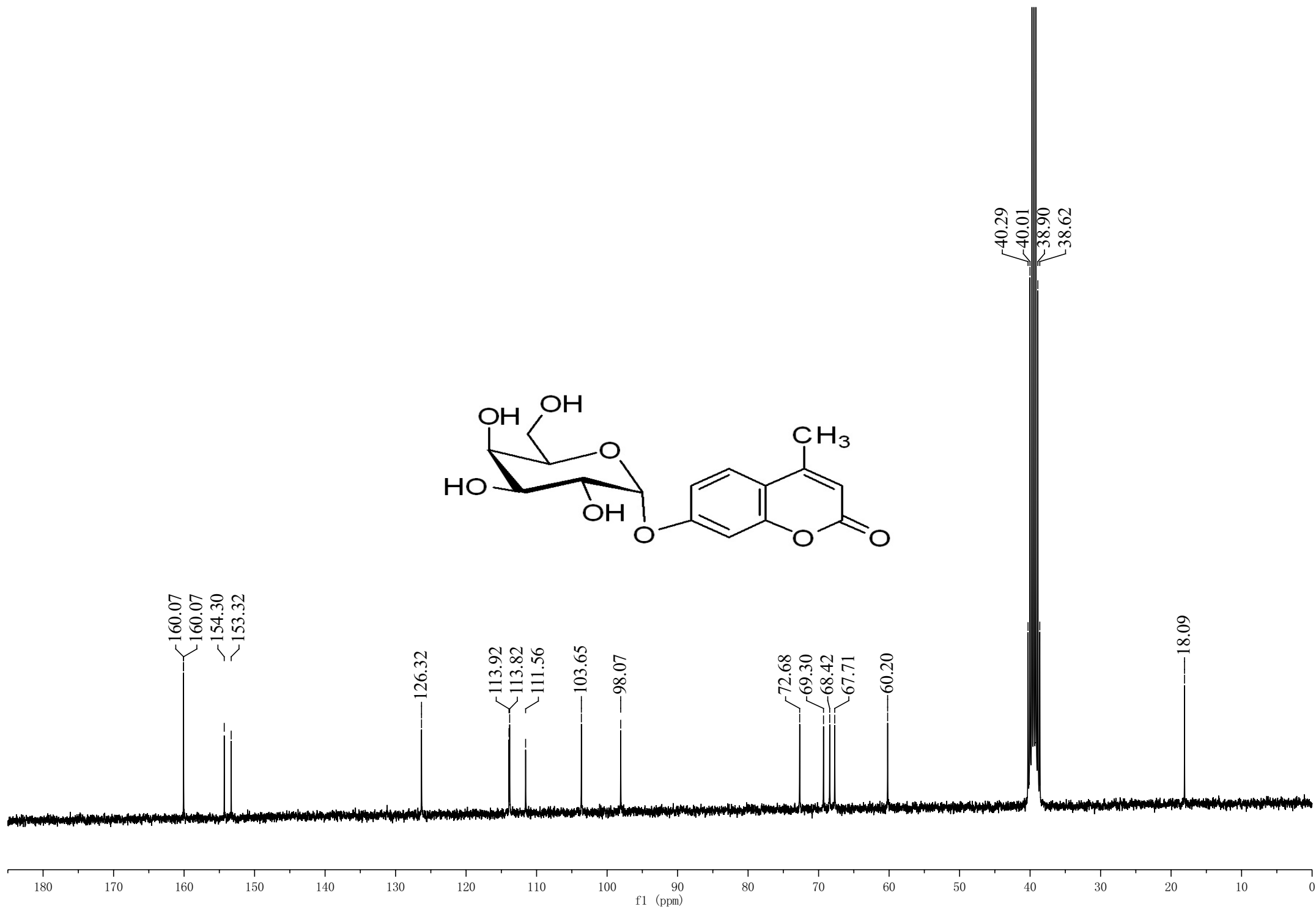

Supplement: Supplementary file 1 [file molecules-20-19789-s001.zip › NMR data.PDF/NMR (4c) the a┴-D-galactopyranoside.pdf]

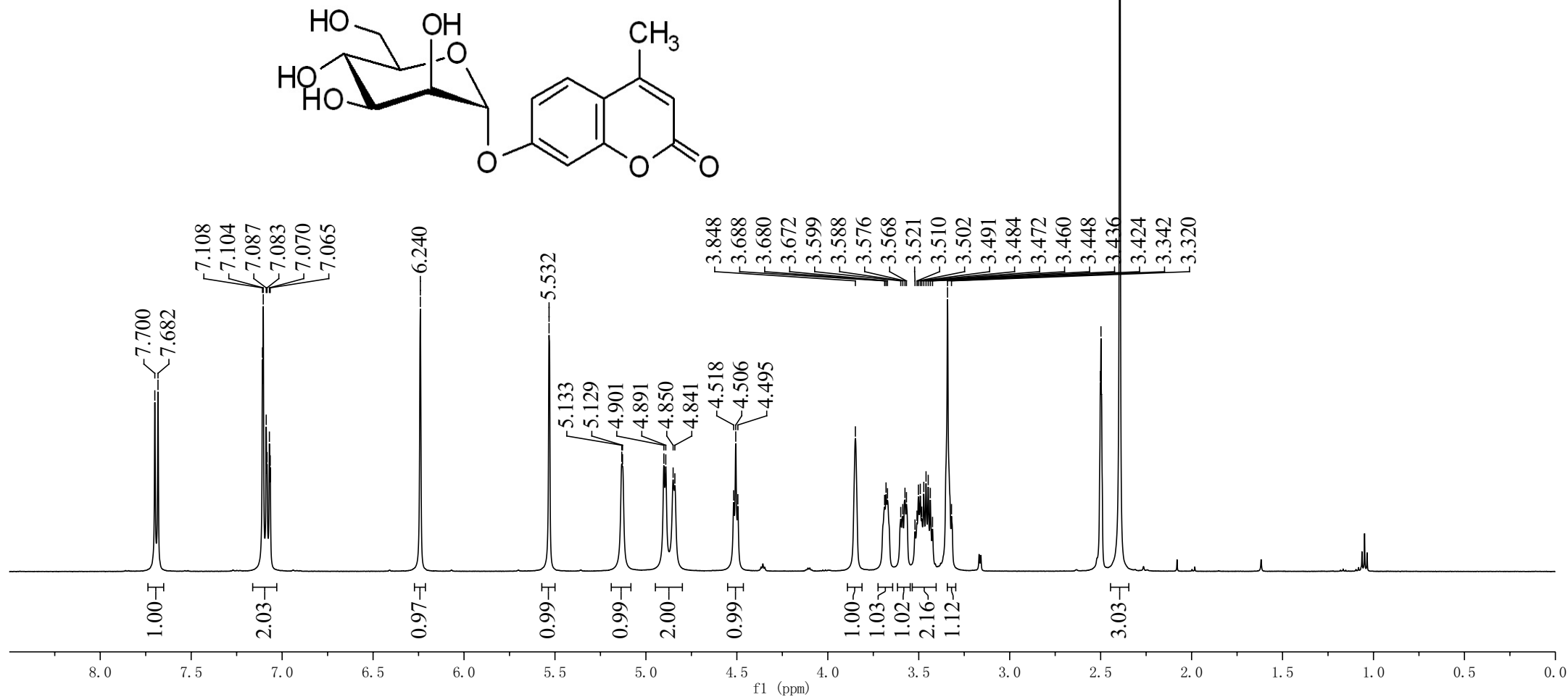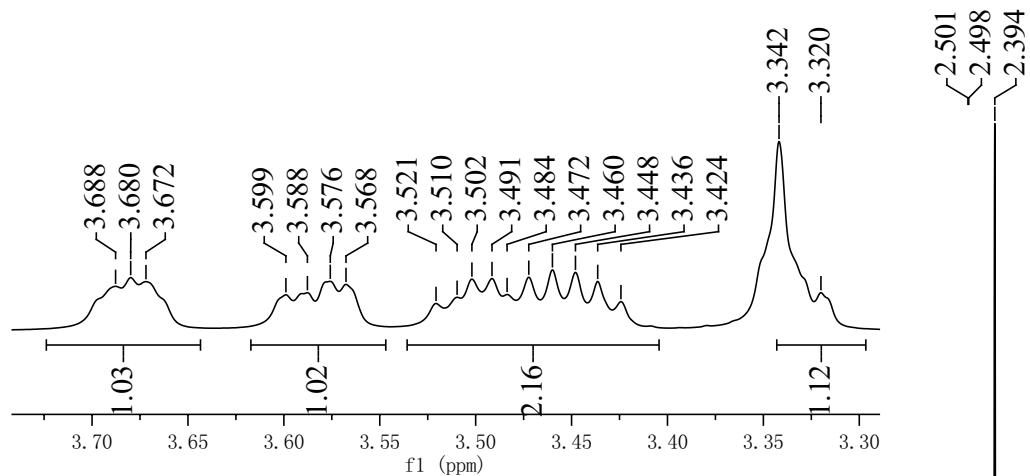

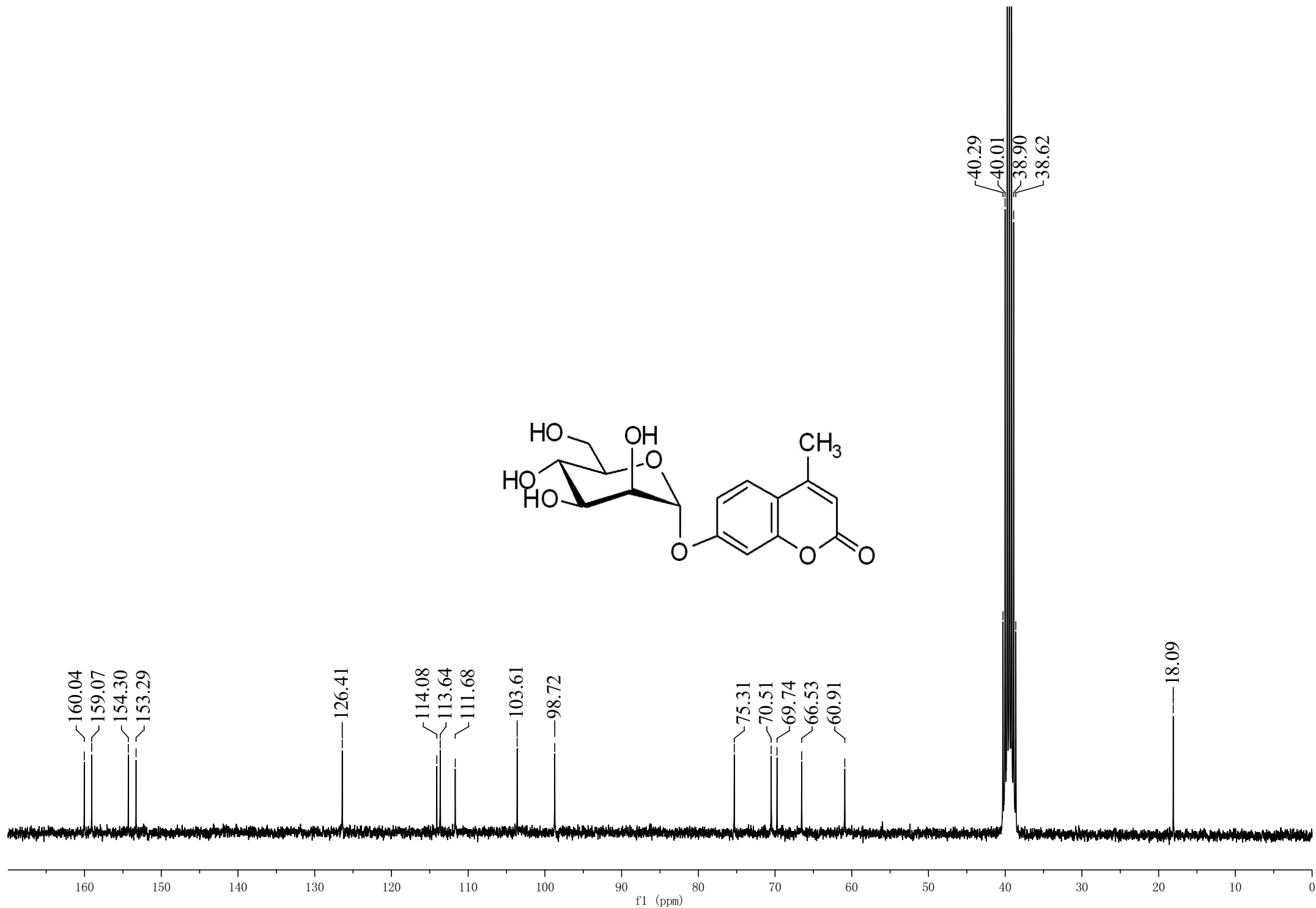

Supplement: Supplementary file 1 [file molecules-20-19789-s001.zip › NMR data.PDF/NMR (4d) the a┴-D-mannopyranoside.pdf]

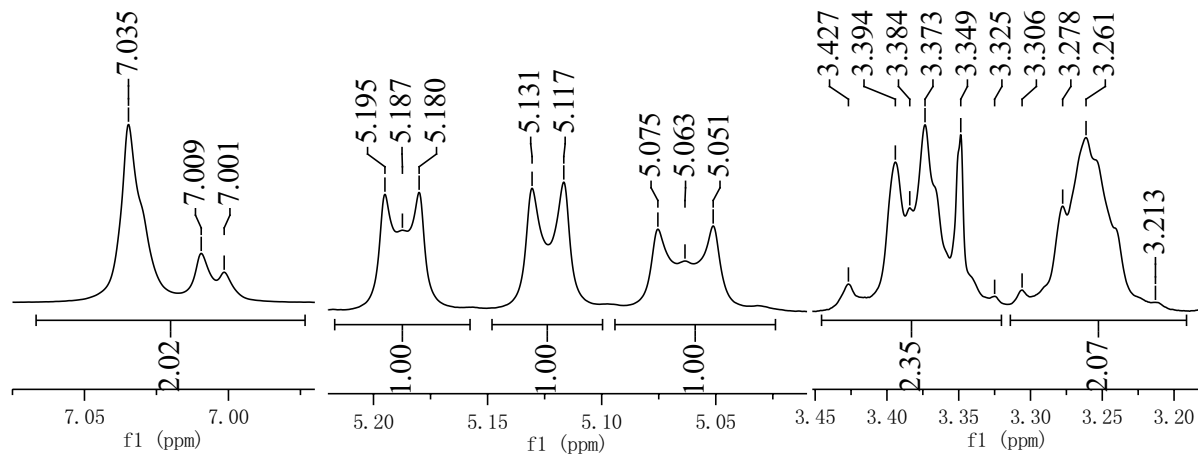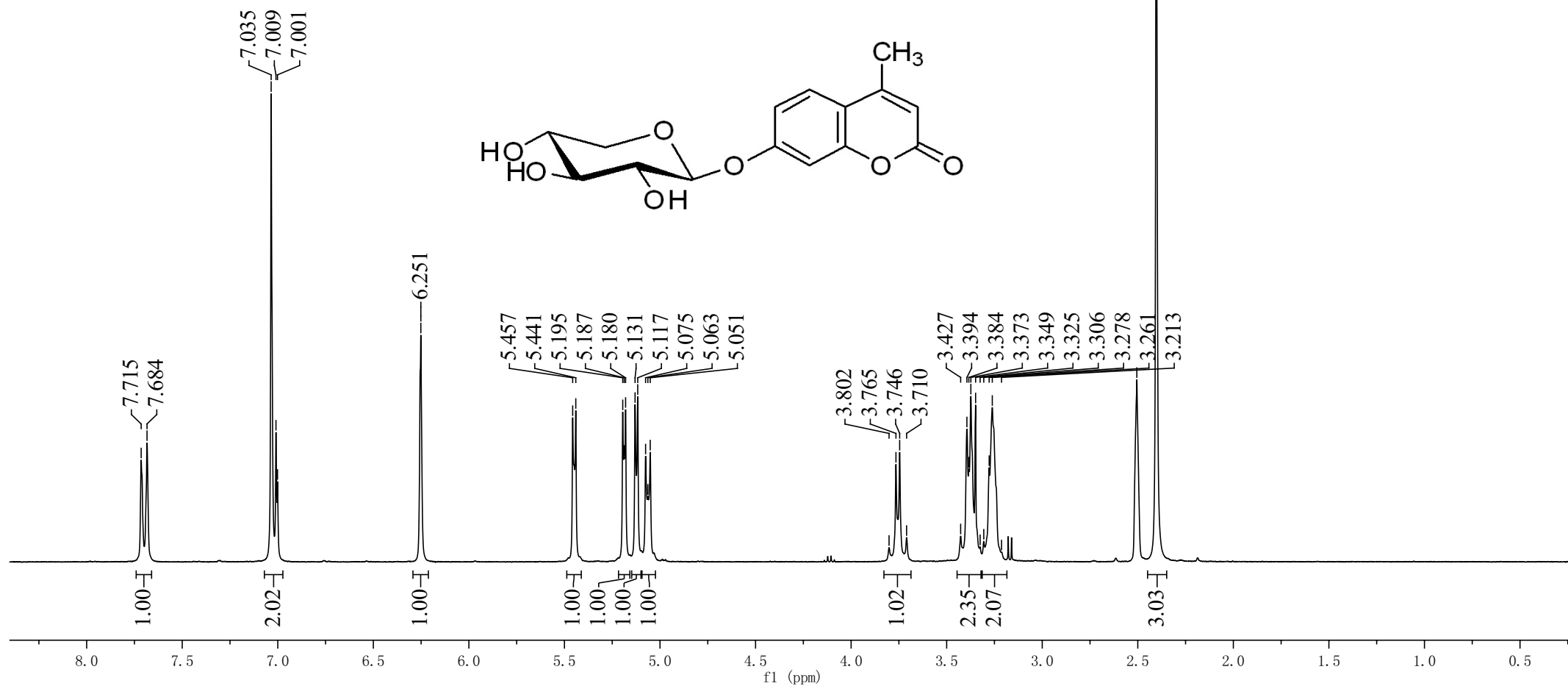

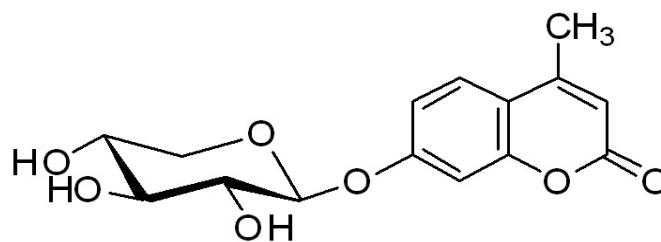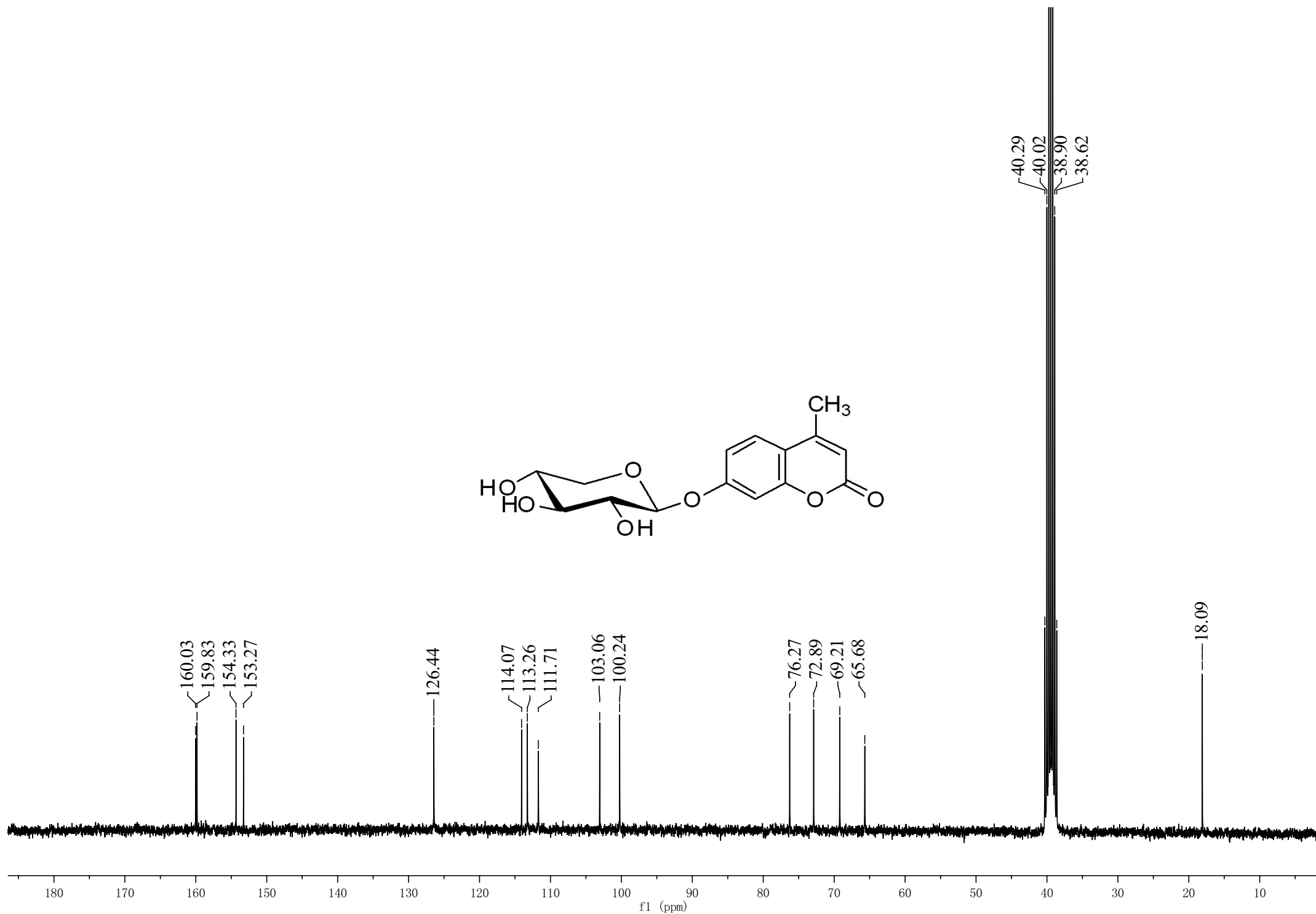

Supplement: Supplementary file 1 [file molecules-20-19789-s001.zip › NMR data.PDF/NMR (4e) the a┬-D-xylopyranoside.pdf]

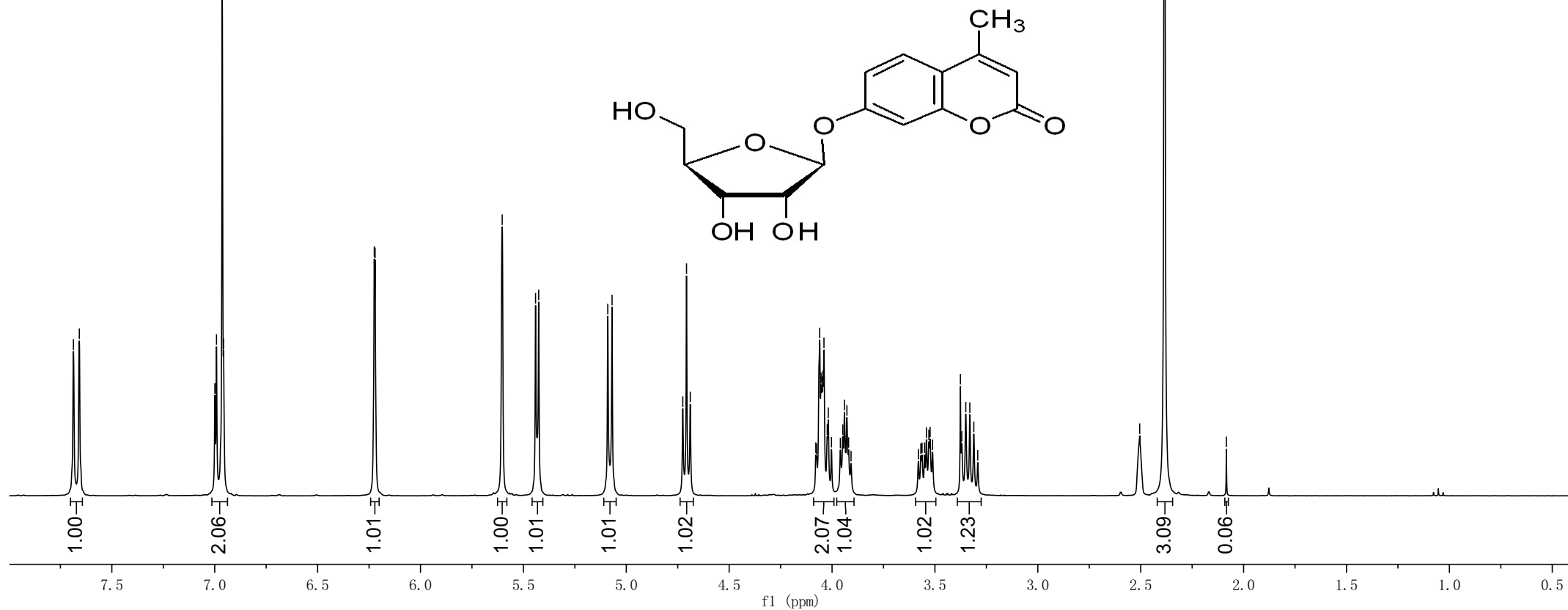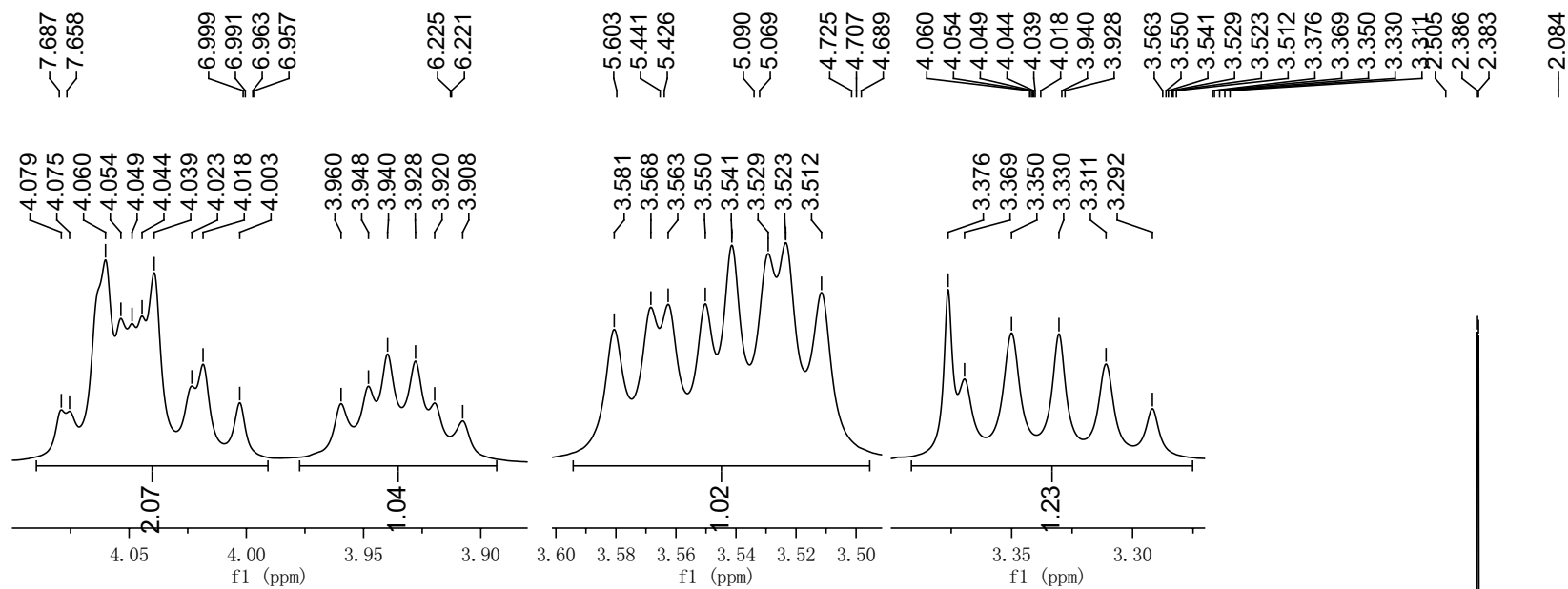

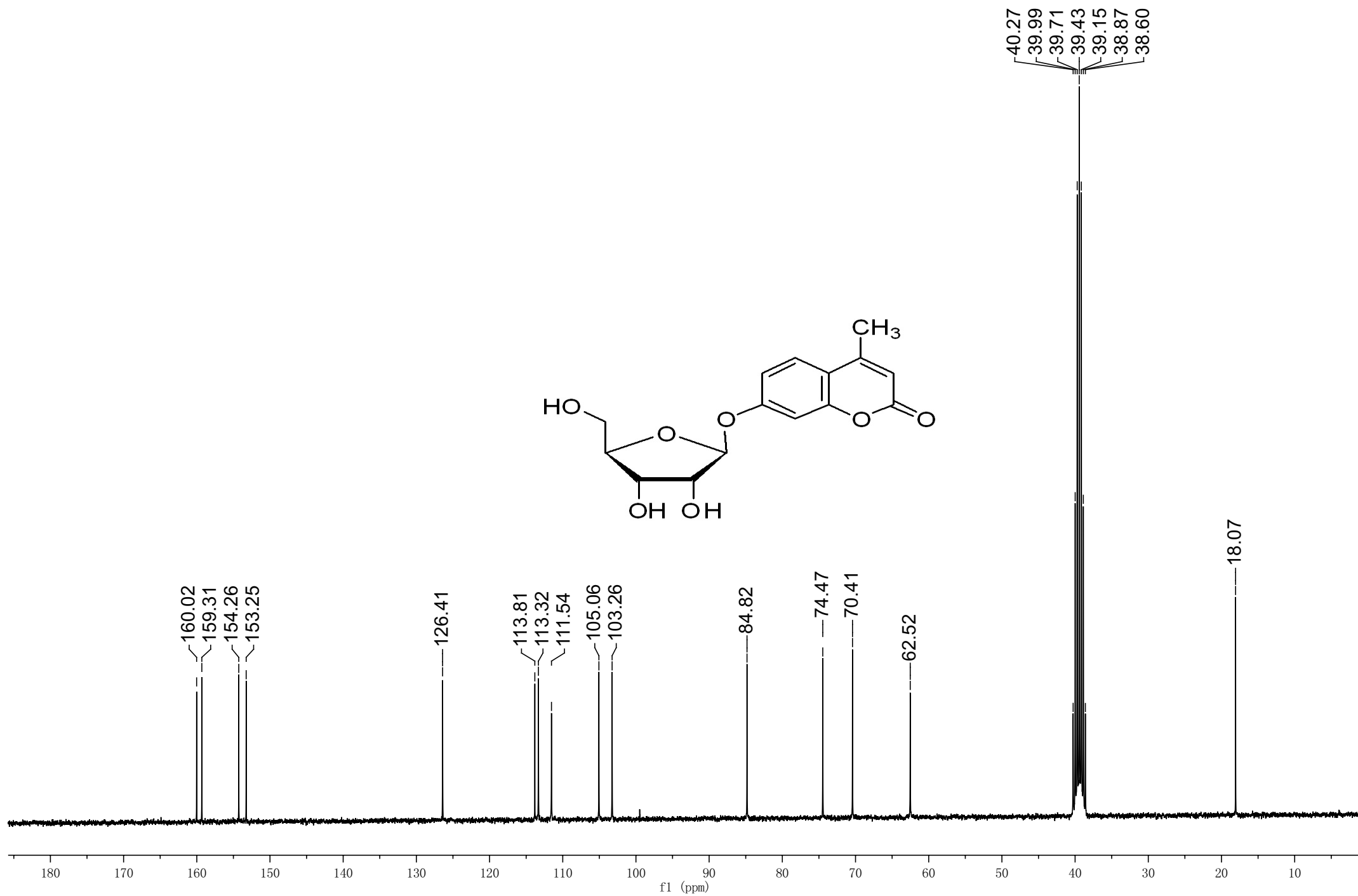

Supplement: Supplementary file 1 [file molecules-20-19789-s001.zip › NMR data.PDF/NMR (4f) the a┬-D-ribofuranoside.pdf]

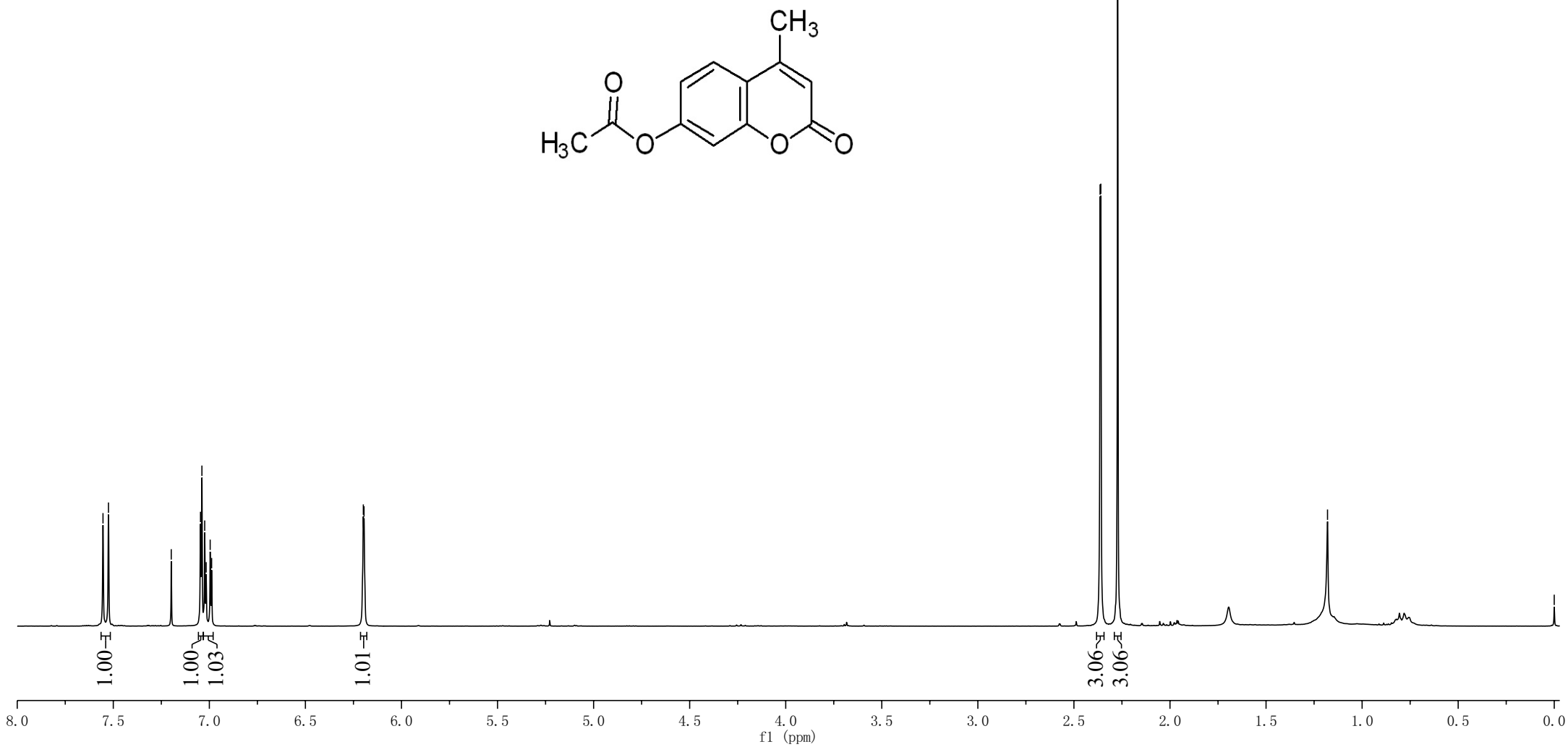

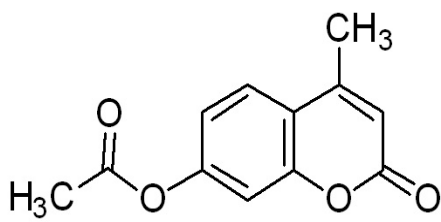

— 168.78  
~ 160.51  
└ 154.16  
└ 153.04  
~ 151.95

— 125.41  
└ 118.12  
└ 117.85  
└ 114.52  
~ 110.47

└ 77.48  
└ 77.26  
└ 77.06  
└ 76.63

— 29.70  
~ 21.13  
~ 18.74

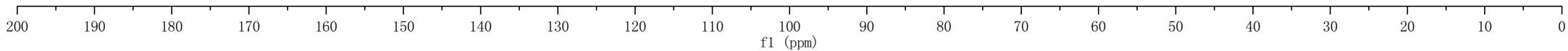

Supplement: Supplementary file 1 [file molecules-20-19789-s001.zip › NMR data.PDF/NMR (5) by-product.pdf]
